# Copper-catalyzed direct transformation of simple alkynes to alkenyl nitriles *via* aerobic oxidative N-incorporation†
†Electronic supplementary information (ESI) available: Characterization data and experimental procedures. See DOI: 10.1039/c5sc02126j


**DOI:** 10.1039/c5sc02126j

**Published:** 2015-07-20

**Authors:** Xiaoqiang Huang, Xinyao Li, Ning Jiao

**Affiliations:** a State Key Laboratory of Natural and Biomimetic Drugs , Peking University , School of Pharmaceutical Sciences , Peking University , Xue Yuan Rd. 38 , Beijing 100191 , China . Email: jiaoning@pku.edu.cn; b State Key Laboratory of Organometallic Chemistry , Chinese Academy of Sciences , Shanghai 200032 , China

## Abstract

A novel direct transformation of aliphatic terminal alkynes to alkenyl nitriles through the incorporation of a nitrogen atom into the simple hydrocarbons has been reported. The usage of inexpensive copper catalyst, O_2_ as the sole oxidant, broad substrate scope as well as feasibility for “late-stage modification” make this protocol very promising. Mechanistic studies including DFT calculation demonstrate a novel 1,2-hydride shift process for this novel nitrogenation reaction.

## Abstract 2

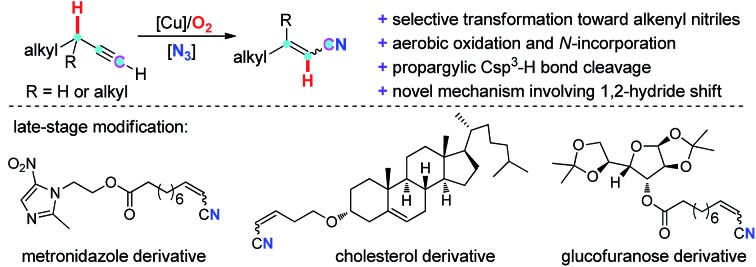
A novel Cu-catalyzed aerobic oxidative N-incorporation into aliphatic terminal alkynes for the synthesis of alkenyl nitriles has been reported. The usage of inexpensive copper catalyst, O_2_ as the sole oxidant, broad substrate scope as well as the feasibility for “the late-stage modification” make this protocol very promising.

## Introduction

Direct transformation of simple and readily available hydrocarbons into complex and high value-added compounds is a long-standing topic in organic synthesis.^1^ Aliphatic alkynes, which are very common and easily accessible building blocks, provide chemists with a fertile testing ground for the construction of complex organic molecules.^2^–^8^ Many useful reactions of these simple hydrocarbons have been disclosed on the basis of the C≡C triple bond transformation, such as coupling,^3^,^4^ addition,^5^ cyclization,^6^ and metathesis reactions.^7^ However, the transformation of simple aliphatic terminal alkynes involving the cleavage of the propargylic C(sp^3^)–H bond is still limited.^8^,^9^

**Scheme 1:**
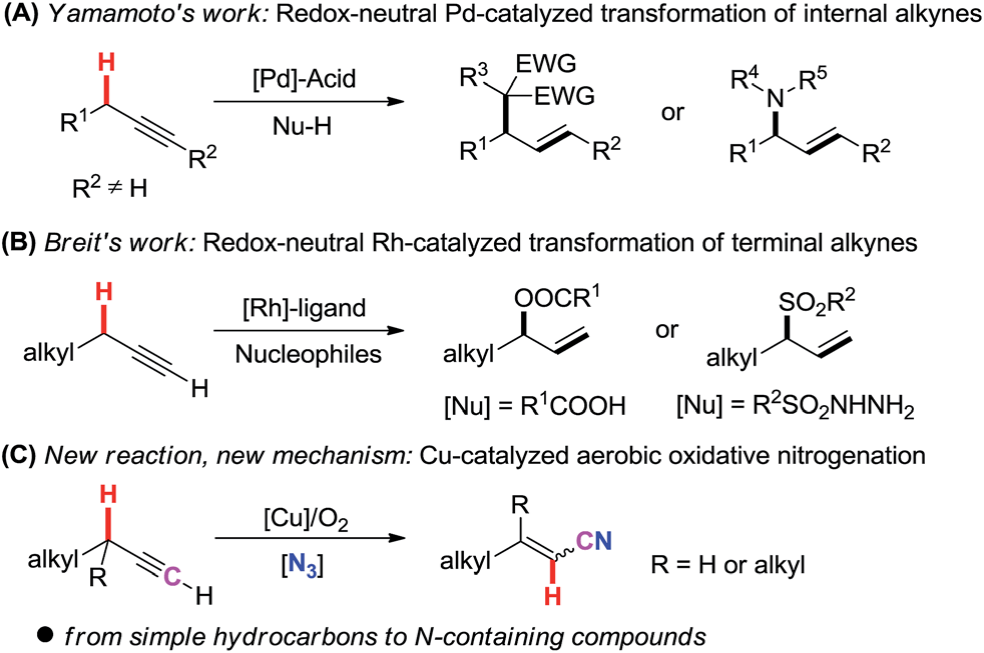
Direct transformation of simple alkynes involving the cleavage of a propargylic C(sp^3^)–H bond.

Recently, novel transformation of simple alkynes has been disclosed through the assistance of transition metals. Yamamoto's group significantly developed palladium/acid catalyzed alkylation and hydroamination reaction of internal alkynes with nucleophiles (Scheme 1, **A**).^10^ By using a Rh(i)/phosphine ligand/benzoic acid catalyst system, Breit and co-workers pioneeringly achieved the intermolecular coupling of aliphatic terminal alkynes with carboxylic acids and sulfonyl hydrazides under redox-neutral conditions (Scheme 1, **B**).^11^ Despite these breakthroughs, new catalytic systems and new strategies are highly desirable to disclose novel transformations of aliphatic terminal alkynes.

Herein, we report a novel Cu-catalyzed aerobic oxidative transformation of simple terminal alkynes to alkenyl nitriles (Scheme 1, **C**). In this present chemistry: (1) a very simple hydrocarbon is successfully converted into an N-containing compound through the incorporation of a nitrogen atom into the substrate; (2) inexpensive Cu-catalyst, the green molecular oxygen oxidant, as well as the broad substrate scope make this protocol very attractive and low-cost; (3) a novel propargylic C(sp^3^)–H bond cleavage through 1,2-H shift mechanism is proved. (4) DFT calculation reasonably explains the mechanism and the stereoselectivity of products.

## Results and discussion

We commenced this research by choosing commercially available 5-phenyl-1-pentyne **1a** as the model substrate. To our delight, the nitrogenation product **2a** was obtained with azidotrimethylsilane (TMSN_3_) as nitrogen source under copper-catalyzed aerobic conditions (Table 1). After extensive screening of different reaction parameters (see ESI† for more information), the direct functionalization of **1a** gave **2a** in 78% yield with a slight *Z*-selectivity (*Z* : *E* = 65 : 35) under the optimized conditions: TMSN_3_ (2.0 equiv.), CuBr (20 mol%), pyridine (2.0 equiv.) and NaOAc (1.0 equiv.), in PhCl at 90 °C under O_2_ (1.0 atm) for 48 h (entry 1, Table 1). As expected, only trace amount of **2a** could be obtained under an argon atmosphere (entry 2). Copper catalyst is essential in this transformation, as no **2a** was formed without copper salt or with other common metal salts (such as [Ag], [Fe], [Co], [Mn], see ESI†). Other copper salts showed lower efficiencies than CuBr (entries 4–5). Further studies indicated that the reaction did not work in the absence of pyridine (entry 7). DMEDA and l-proline led to no reaction (entries 8–9). Catalytic amount of pyridine only delivered **2a** in 26% yield (entry 10). It is noteworthy that NaOAc, a very weak base, is the most effective additive while not indispensable for product formation (entries 11–13).^12^ However, great efforts to improve the *Z* : *E* ratio of the product did not reach a satisfying result (see ESI† for more information).

**Table 1:** Selected optimization of the reaction conditions^*a*^

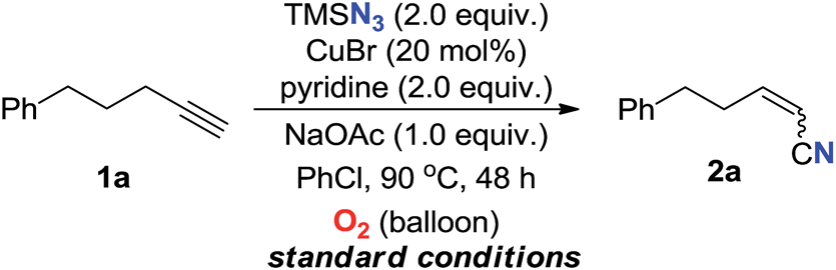			
Entry	Variation from *standard conditions*	Yield^*b*^ (%)	*Z* : *E*^*c*^
**1**	**None**	**78**	**63** **:** **35**
2	Ar instead of O_2_	Trace	—
3	Without CuBr	0	—
4	Cu(OAc)_2_ instead of CuBr	49	69 : 31
5	CuBr_2_ instead of CuBr	62	67 : 33
6	10 mol% CuBr was employed	45	65 : 34
7	Without pyridine	0	—
8	DMEDA instead of pyridine	0	—
9	l-proline instead of pyridine	0	—
10	0.4 equiv. pyridine was employed	26	69 : 31
11	Without NaOAc	48	64 : 36
12	LiOAc instead of NaOAc	48	64 : 36
13	NaOMe instead of NaOAc	47	67 : 33

^*a*^ *Standard conditions*: **1a** (0.40 mmol), TMSN_3_ (0.80 mmol), CuBr (0.08 mmol), pyridine (0.80 mmol) and NaOAc (0.40 mmol) in PhCl (2.0 mL) under O_2_ (balloon) was stirred at 90 °C for 48 h.

^*b*^Isolated yields.

^*c*^Determined by ^1^H NMR measurement of the crude mixture. DMEDA = *N*,*N*′-dimethyl-1,2-ethanediamine.

With the optimal conditions in hand, we next investigated the substrate scope of this transformation. This reaction exhibited a good functional group compatibility (Table 2). Long-chain-alkyl substituted alkynes were successfully transformed to the corresponding alkenyl nitriles in good yields (**2a–2e**). Notably, propargylic 3° C–H of **1f** could be cleaved, giving **2f** in 61% yield. To our satisfaction, terminal alkyne **1g**, bearing a TBDMS protected hydroxyl group, worked well (**2g**, 68%). Remarkably, linkages, including ether bonds (**2h–2n**) and ester bonds (**2o–2q**), did not reduce effectiveness. Several functional groups (trifluoromethyl, chlorine, vinyl and thienyl) were well tolerated in the present catalytic system. Furthermore, reasonable yields were obtained for alkynes containing phthalimide and sulfonamide group, respectively (**2r–2s**). Interestingly, C–H bond adjacent to internal ethynyl group was inactive, which leads to the high regioselectivity.

**Table 2:** The scope of terminal alkynes^*a*^

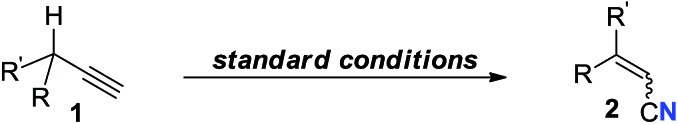				
Entry	**2**		Yield of **2**^*b*^ (%)	*Z* : *E*^*c*^
1	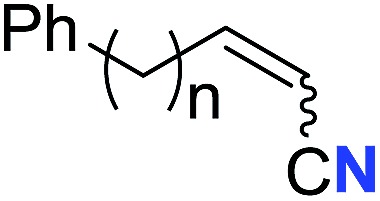	*n* = 3	78 (**2a**)	65 : 35
2		*n* = 2	44 (**2b**)	67 : 33
3	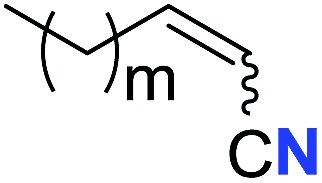	*m* = 7	76 (**2c**)	61 : 39
4		*m* = 6	62 (**2d**)	66 : 34
5	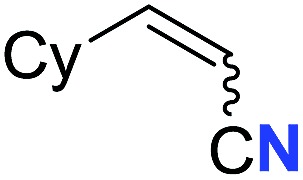		63 (**2e**)	60 : 40
6	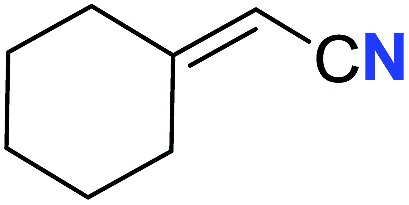		61 (**2f**)	—
7	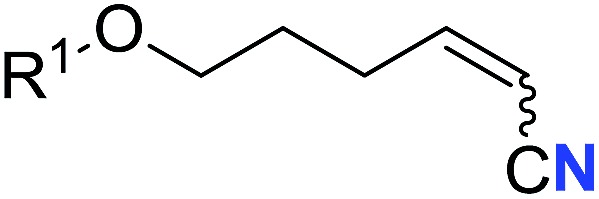	R^1^ = TBDMS	68 (**2g**)	69 : 31
8		R^1^ = *n*-C_9_H_19_	59 (**2h**)	69 : 31
9	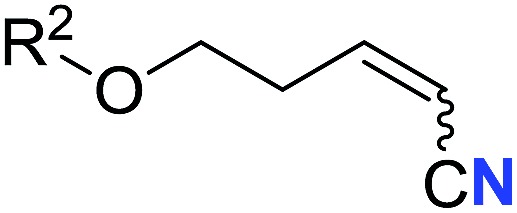	R^2^ = C_6_H_5_	60 (**2i**)	68 : 32
10		R^2^ = 4-MeOC_6_H_4_	50 (**2j**)	69 : 31
11		R^2^ = 4-CF_3_C_6_H_4_	66 (**2k**)	63 : 37
12		R^2^ = 2-ClC_6_H_4_	71 (**2l**)	66 : 34
13		R^2^ = 1-naphth	60 (**2m**)	64 : 36
14		R^2^ = 2-naphth	57 (**2n**)	64 : 36
15	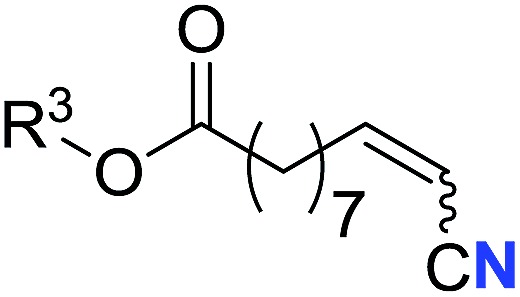	R^3^ = Me	69 (**2o**)	69 : 31
16^*d*^		R^3^ = 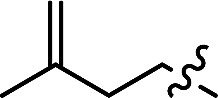	40 (**2p**)	64 : 36
17	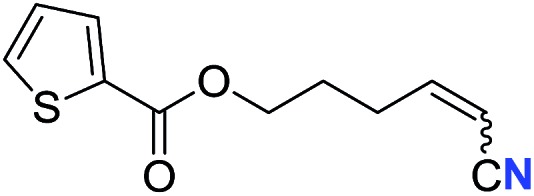		73 (**2q**)	66 : 34
18	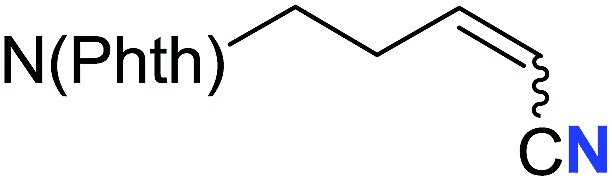		65 (**2r**)	69 : 31
19	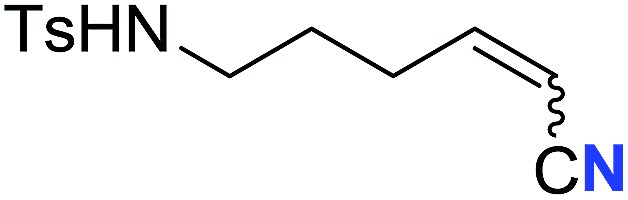		61 (**2s**)	70 : 30
20	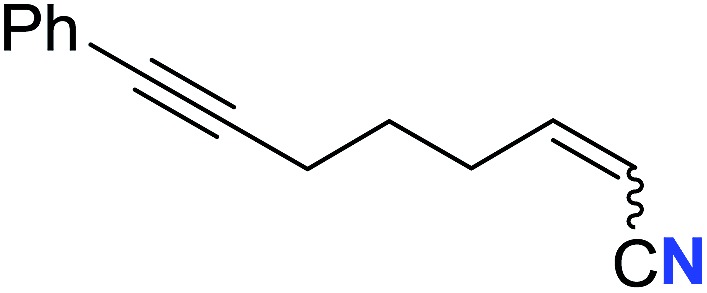		46 (**2t**)	66 : 34

^*a*^ *Standard conditions*: see entry 1, Table 1.

^*b*^Isolated yields.

^*c*^Determined by ^1^H NMR measurement of the crude mixture.

^*d*^Two portions of TMSN_3_ (0.60 mmol) were added every 24 h.

Alkenyl nitriles are not only useful building blocks in synthetic chemistry but also important structure motifs commonly found in drugs.^13^ Moreover, late-stage modification is a highly valuable strategy for medicinal chemistry research.^14^ Therefore, several complex bioactive molecule derivatives were submitted to the optimal conditions (Table 3). Natural alcohol derivatives containing ester or ether linkages, such as menthol, borneol, nopol and cholesterol, worked well in the current transformation, generating the corresponding alkenyl nitriles in 49–73% yield (**4a**, **4d–f**), respectively. Alkyne **3b** that was prepared from antibacterial metronidazole afforded alkyl alkenyl nitrile **4b** in 60% yield. Besides, terminal alkyne with a protected sugar moiety selectively underwent aerobic oxidation, giving nitrogenation product **4c** in 67% yield. These results demonstrate that the present protocol could be applied in late-stage bioactive compound modification.

**Table 3:** The late-stage modification^*a*^

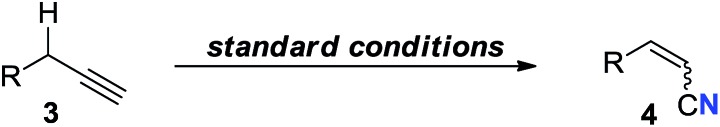
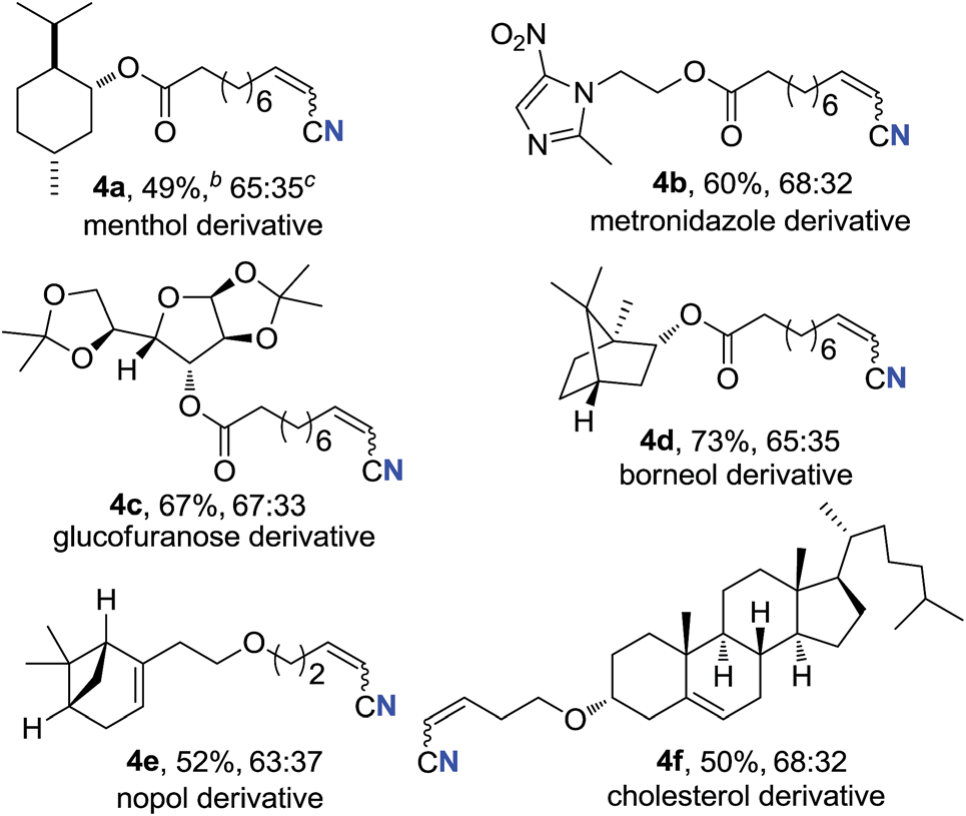

^*a*^ *Standard conditions*: see entry 1, Table 1.

^*b*^Isolated yields.

^*c*^Determined by ^1^H NMR measurement of the crude mixture.

To gain mechanistic insight into this transformation, some control experiments were conducted under the standard conditions. Allene **5**, which could be generated from alkyne **1a**, failed to afford nitriles under the present conditions (eqn (1)), indicating a novel mechanism different from Breit's works.^11^ In addition, propargylic azide **6** or allylic azide **7** could not furnish alkenyl nitriles either (eqn (2) and (3)). These results ruled out the possibility of **6** and **7** as intermediates of the transformation.^15^
1

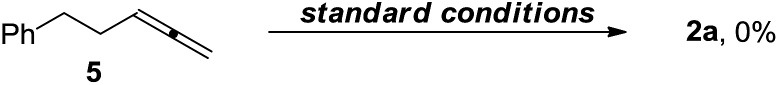



2

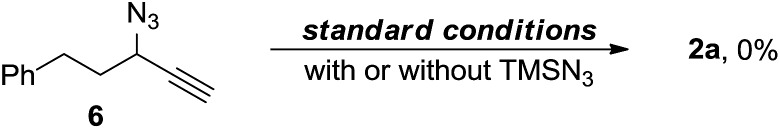



3

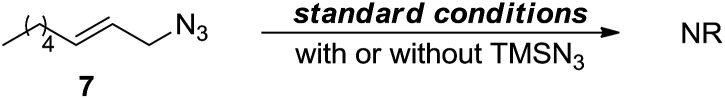




Considering that the current nitrogenation reaction could only be catalyzed by copper salt, and the Glaser–Hay homocoupling product3*a* could be detected in some cases, we postulated that copper acetylide might be an intermediate of this reaction. Although, no product was formed employing copper(i)-acetylide **8** as a substrate (eqn (4)), which might due to the aggregation of **8**,^16^**2c** could be obtained in comparative yield when **8** was used as a catalyst (eqn (5)). When C(sp)–H bond deuterated alkynes **1a-1-*d*_1_** was subjected to the reaction, no deuterium was detected in the product, which is in accordance with the existence of copper acetylide species (eqn (6)).
4

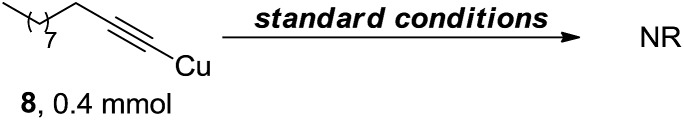



5

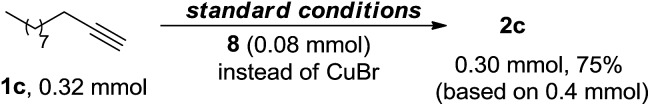




Furthermore, labeling experiment with propargylic C–H bond deuterated **1a-3,3-*d*_2_** was performed. To our surprise, nearly 100% incorporation of deuterium at the both α and β positions of the nitrile was observed (eqn (7)). Hence, the cleavage of propargylic C–H bond might proceed *via* a 1,2-hydride shift.^17^ Then, an intermolecular kinetic isotopic experiment was conducted giving the result of *k*_H_/*k*_D_ = 2.2 (eqn (8)).^18^
6

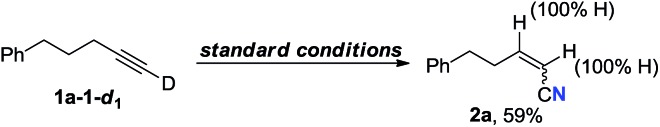



7

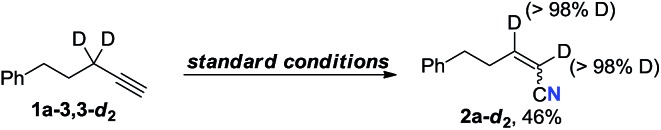



8

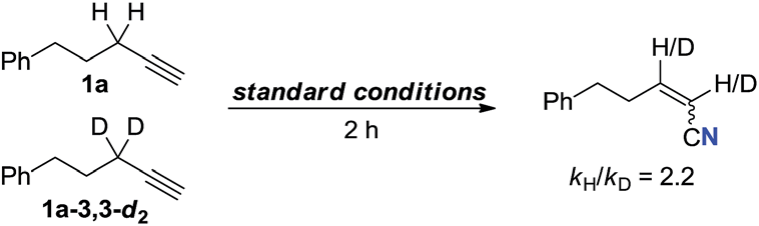




**Scheme 2:**
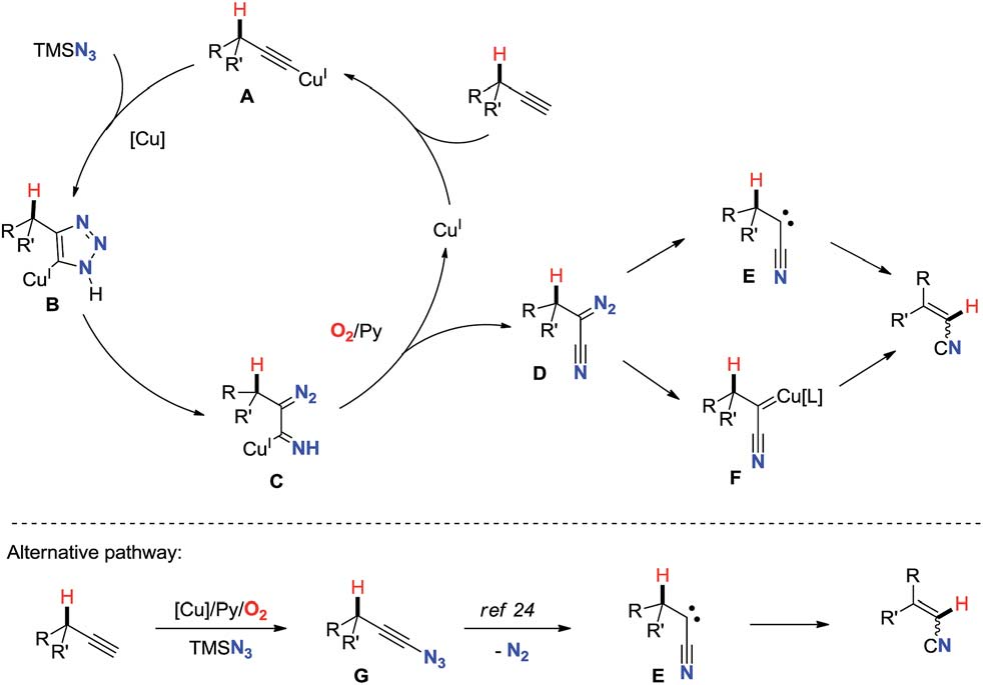
Proposed mechanism.

On the basis of all these results and previous reports, a proposed mechanism is depicted in Scheme 2. The reasonable first step is the formation of copper(i)-acetylide intermediate **A**.^16^ Then, copper triazolide **B** formed *via* Cu-catalyzed azide–alkyne cycloaddition (CuAAC)^19^ undergoes ring-opening reaction affording cuprated diazoimine **C**.^20^,^21^ The oxidation of **C** under aerobic conditions with assistance of pyridine gives α-diazonitrile **D** and regenerates the copper(i) catalyst.^22^ Subsequently, upon loss of dinitrogen **D** would afford carbene **E** or copper carbene **F**.^23^ Finally, 1,2-hydride shift of the carbene species generates the alkenyl nitrile.^17^ Alternatively, a mechanism with ethynyl azide could also be possible. Cu-catalyzed aerobic oxidative cross-coupling of terminal alkynes with TMSN_3_ might generate ethynyl azide **G**,3b,^4^ which is known to liberate dinitrogen leading to the formation of cyanocarbene species **E**.^24^

**Fig. 1:**
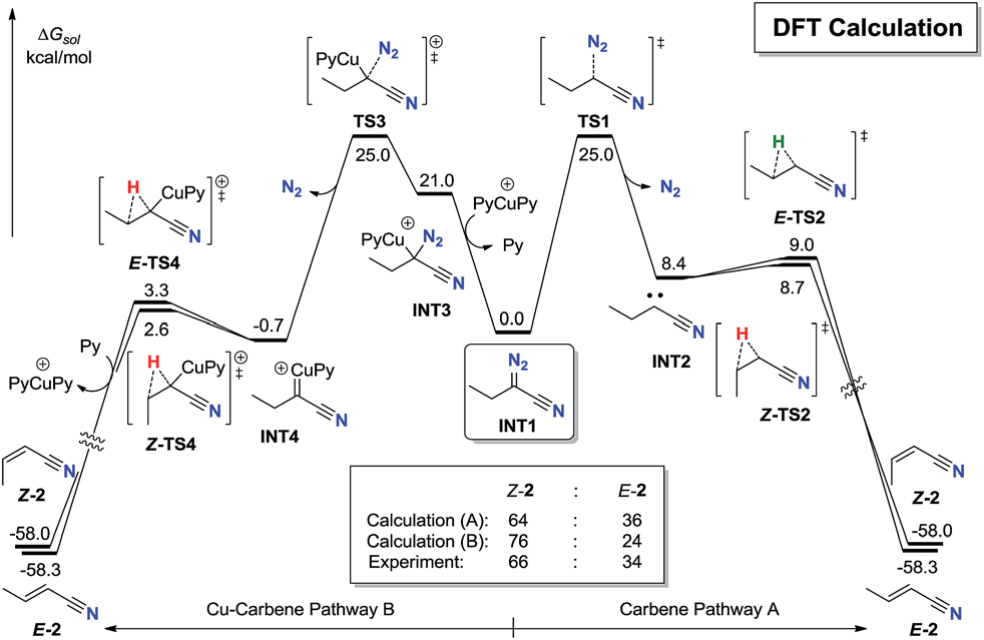
DFT-computed energy profiles.

To further explore the stereoselectivity of the reaction, density functional theory (DFT) calculation investigation was carried out (Fig. 1).^25^ After the sequential CuAAC^19^ and ring-opening process,^20^,^21^ the α-diazonitrile **INT1** is generated (see ESI† for details). The pyrolysis of **INT1** has two pathways. In pathway A, the thermal induced release of N_2_ through **TS1** requires an activation free energy of 22.3 kcal mol^–1^ to give cyanocarbene carbene **INT2**. Alternatively, **INT2** generated from ethynyl azide could not be excluded.^24^ The subsequent 1,2-hydride shift process^17^*via****Z*-TS2** and ***E*-TS2** almost barrierlessly delivers ***Z*-2** and ***E*-2**, respectively. It is noteworthy that the energy barrier gap between ***Z*-TS2** and ***E*-TS2** is insignificant (only 0.3 kcal mol^–1^), which might be due to the similar steric hindrance between hydrogen and cyano group. The calculated *Z* : *E* ratio of **2***via* pathway A is predicted to be 64 : 36, which is qualitatively consistent with the experimentally observed 66 : 34 *Z* : *E* ratio for this reaction.

In alternative pathway B, Cu(i) catalyst can induce the Cu–C bond formation on **INT3** with the release of N_2_ through **TS3** in a stepwise manner, which requires an activation free energy of 22.3 kcal mol^–1^ to form Cu-carbene **INT4**.^23^ The subsequent 1,2-H shift process^17^*via****Z-*TS4** and ***E*-TS4** also barrierlessly furnishes ***Z*-2** and ***E*-2**, respectively. Notably, ***Z-*TS4** is also only 0.7 kcal mol^–1^ lower in energy than ***E-*TS4**, which is corresponding to a 76 : 24 *Z* : *E* ratio of **2**, in good agreement with the experimental observation.

Moreover, ***E*-2** is only 0.3 kcal mol^–1^ lower in energy than ***Z*-2**, indicating that the *Z* to *E* isomerization of alkenyl nitriles **2** is short of driving force thermodynamically. These results could explain why the *E* : *Z* ratio of the products is so difficult to optimize whether by dynamic or thermodynamic means.

## Conclusions

In conclusion, we have developed a novel copper-catalyzed aerobic oxidative nitrogenation of simple alkyl alkynes *via* propargylic C(sp^3^)–H bond cleavage. A variety of simple and easily accessible alkynes selectively undergo the transformation affording alkenyl nitriles. The late-stage modification of bioactive molecule derivatives makes this protocol very attractive. Mechanism studies indicate a 1,2-hydride shift might be the key step of this novel transformation. DFT calculation reasonably explains the stereoselectivity of products. Further studies on the mechanism and the application are ongoing in our group.

## Supplementary Material

Supplementary information
