# Supplementary material for: Copper-catalyzed direct transformation of simple alkynes to alkenyl nitriles via aerobic oxidative N-incorporation
Source: Chem Sci. 2015 Jul 20;6(11):6355–60. doi: 10.1039/c5sc02126j (PMC6054104; doi:10.1039/c5sc02126j)
Supplement: Supplementary file 1 [file SC-006-C5SC02126J-s001.pdf]

Electronic Supplementary Material (ESI) for Chemical Science.
This journal is © The Royal Society of Chemistry 2015

Electronic Supplementary Information for:

Copper-catalyzed direct transformation of simple alkynes to alkenyl nitriles via aerobic oxidative N-incorporation

Xiaoqiang Huang,[a] Xinyao Li[a] and Ning Jiao[ab]*

[a] State Key Laboratory of Natural and Biomimetic Drugs, School of Pharmaceutical Sciences, Peking University, Xue Yuan Rd. 38, Beijing 100191, China

[b] State Key Laboratory of Organometallic Chemistry, Chinese Academy of Sciences, Shanghai 200032, China

E-mail: jiaoning@pku.edu.cn; Fax: (+86)10-82805297

Table of Contents

General Remarks.	S1
Screening of Different Reaction Parameters	S2-S9
Experimental Procedure and Characterization Data for Products	S10-S26
Mechanistic Studies	S27-S31
Computational Methods	S32-S33
Computed Energies of All Stationary Points	S34
Coordinates of All Stationary Points	S35-S38
References	S39-S40
^{1}H NMR and ^{13}C NMR Spectra of Products	S41-S99

General Remarks

All commercially available compounds were purchased from Sigma-Aldrich, Alfa-Aesar, Acros, Beijing Ouhe and Beijing Chemical Works, Ltd. Unless otherwise noted, materials obtained from commercial suppliers were used without further purification. Analysis of crude reaction mixture was done on an Agilent 7890 GC System with an Agilent 5975 Mass Selective Detector. Products were purified by flash chromatography on silica gel. ^{1}H-NMR spectra were recorded on Bruker AVANCE III-400 spectrometers. Chemical shifts (in ppm) were referenced TMS in $CDCl_3$ (0 ppm). ^{13}C-NMR spectra were obtained by using the same NMR spectrometers and were calibrated with $CDCl_3$ (δ = 77.00 ppm). Mass spectra were recorded using a PE SCLEX QSTAR spectrometer. High resolution mass spectra were obtained with a Bruker APEX IV Fourier transform ion cyclotron resonance mass spectrometer. Infrared spectra were recorded on a Nicolet Avatar 330 Fourier transform spectrometer (FT-IR) and are reported in wave numbers (cm^{-1}).

Screening of Different Reaction Parameters

Table S1 Screening of pyridine derivates[a]

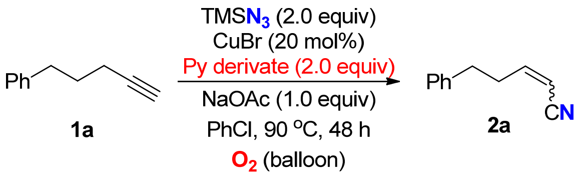

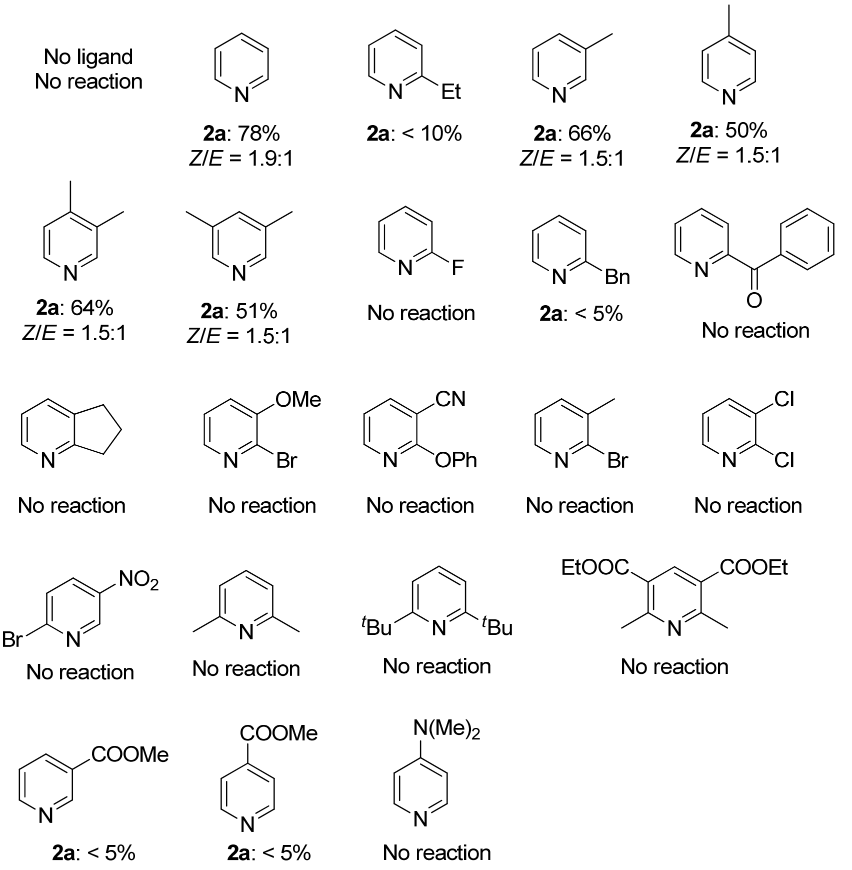

[a]Reaction conditions: **1a** (0.20 mmol), $TMSN_3$ (0.40 mmol), CuBr (0.04 mmol), pyridine derivates (0.40 mmol), and NaOAc (0.20 mmol) in PhCl (1.0 mL) was stirred at 90 °C for 48 h. Yield and *Z/E* ratio were determined by ^{1}H NMR measurement of the crude mixture.

Table S2 Screening of ligands-1[a]

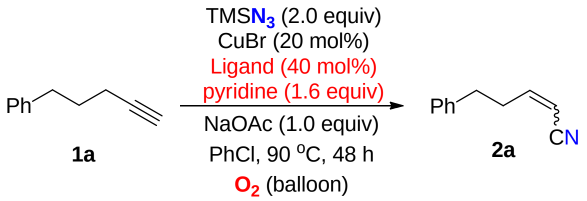

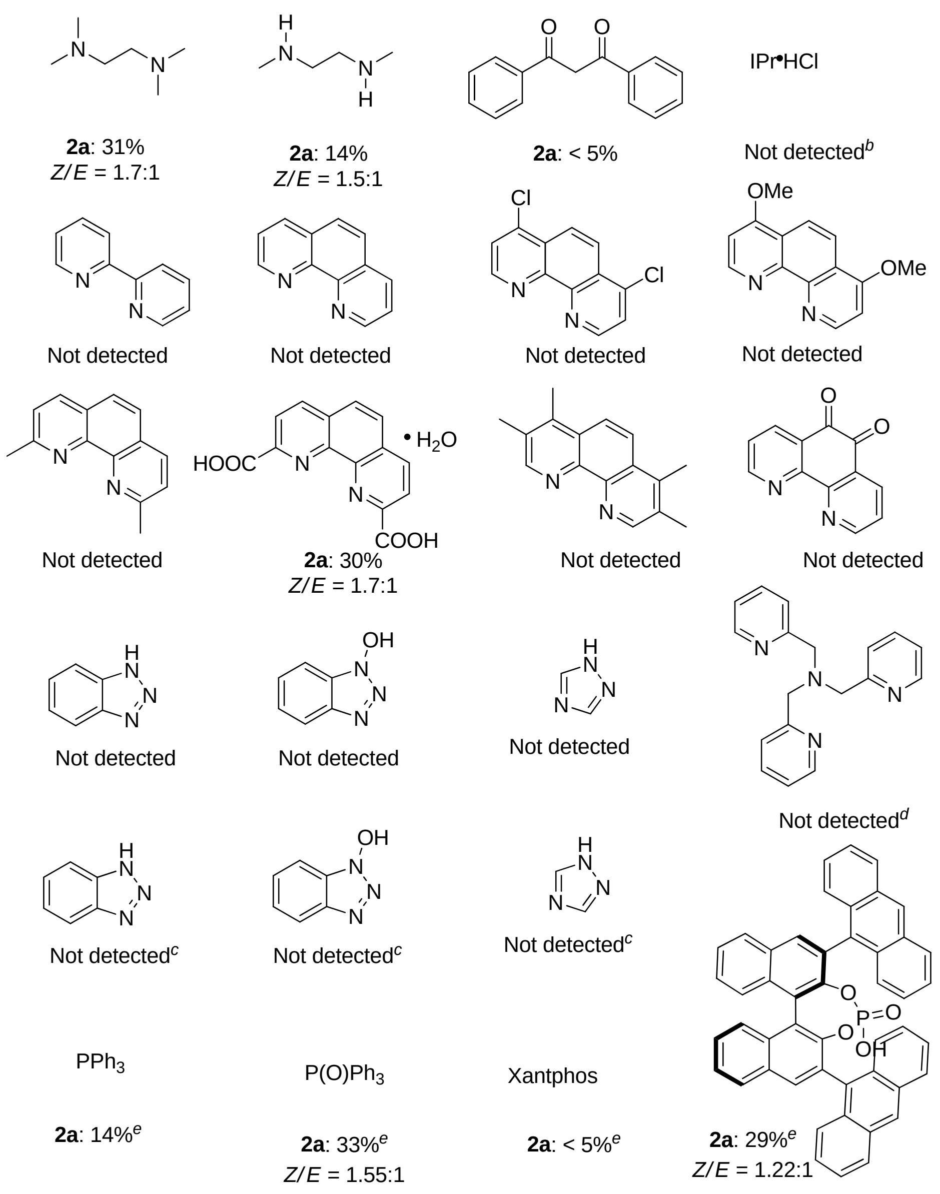

[a]Reaction conditions: **1a** (0.20 mmol), $TMSN_3$ (0.40 mmol), CuBr (0.04 mmol), **Ligand (40 mmol%), Pyridine** (0.32 mmol), and NaOAc (0.20 mmol) in PhCl (1.0 mL) was stirred at 90 °C for 48 h. Yield and *Z/E* ratio were determined by ^{1}H NMR measurement of the crude mixture. [b]**Pyridine** (0.40 mmol) was employed. [c]**Ligand** (0.40 mmol) was employed without **Pyridine**. [d]**Ligand** (0.10 mmol) was employed without **Pyridine**. [e]**Ligand (20 mmol%), Pyridine** (0.40 mmol) was employed without NaOAc.

Table S3 Screening of ligands-2[a]

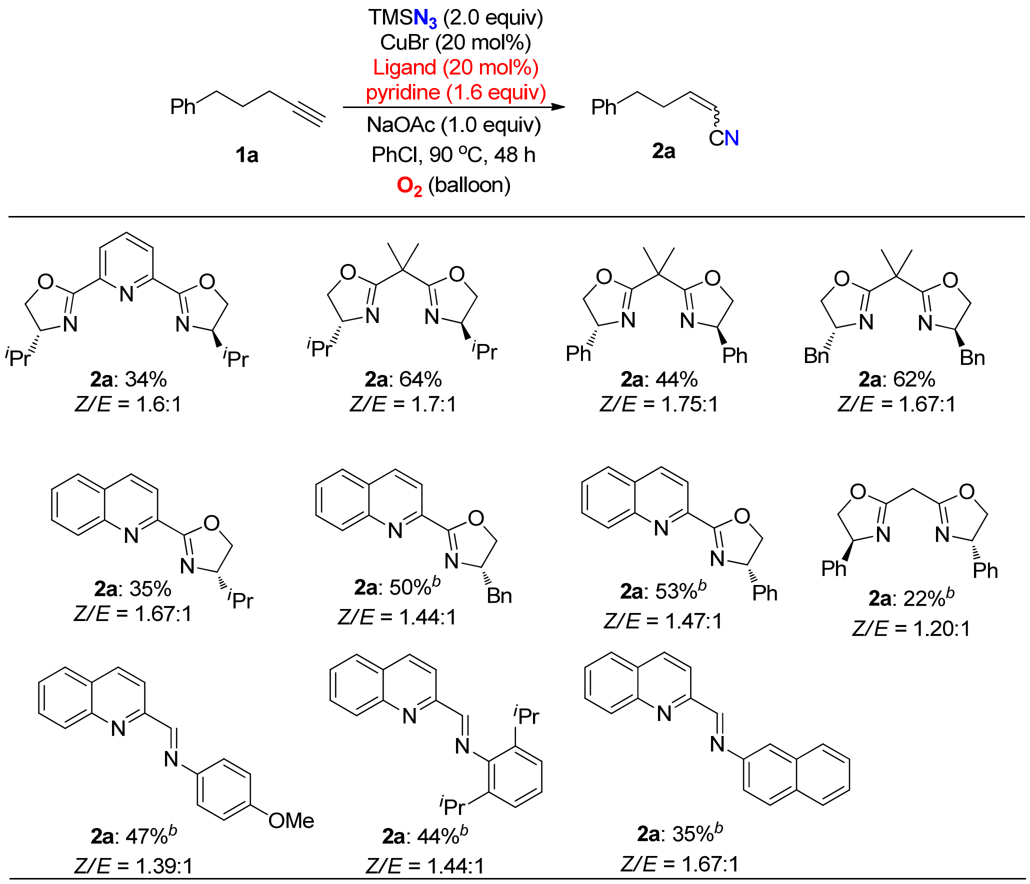

[a]Reaction conditions: **1a** (0.20 mmol), $TMSN_3$ (0.40 mmol), CuBr (0.04 mmol), **Ligand (20 mmol%), Pyridine** (0.32 mmol), and NaOAc (0.20 mmol) in PhCl (1.0 mL) was stirred at 90 °C for 48 h. Yield and *Z/E* ratio were determined by ^{1}H NMR measurement of the crude mixture. [b]without **NaOAc.**

Table S4 Screening of metal[a]

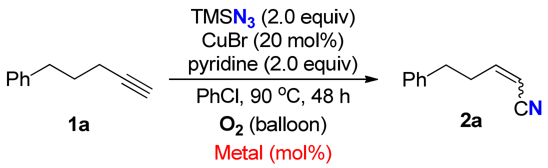

Entry	Metal (mol%)	Yield of **2a**[b]	*E/Z*[c]
1	$Rh_2(OAc)_4$ (2 mol%)	41%	1.47:1
2	$Rh_2(Oct)_4$ (2 mol%)	34%	1.39:1
3	$Rh(PPh_3)_3Cl$ (5 mol%)	37%	~ 2:1
4	$[Cp^*RhCl_2]_2$ (2.5 mol%)	25%	1.5:1
5	$Pd(OAc)_2$ (5 mol%)	< 20%	
6	$Fe(OAc)_2$ (10 mol%)	43%	1.44:1
7	$[Ru(p\text{-cymene})Cl_2]_2$ (2.5 mol%)	34%	1.39:1

[a]Reaction conditions: **1a** (0.20 mmol), $TMSN_3$ (0.40 mmol), CuBr (0.04 mmol), pyridine (0.40 mmol), and **Metal (2-10 mol%)** in PhCl (2.0 mL) was stirred at 90 °C for 48 h. [b]NMR yield. [c]Determined by ^1H NMR measurement of the crude mixture.

Table S5 Screening of the additives[a]

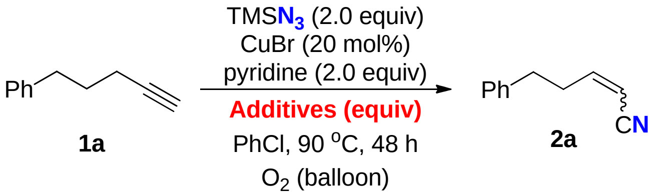

Entry	Additives (equiv)	Yield of **2a** (%)[b]	*Z/E*[c]
1	none	48	64:36
**2**	**NaOAc (1.0)**	**78**	**65:35**
3	LiOAc (1.0)	50	64:36
4	NaOMe (1.0)	47	67:33
5[d]	mCPBA (0.15)	50	63:37
6[d]	$FeCl_2$ (0.1)	43	64:36
7[d]	$Yb(OTf)_3{\bullet}H_2O$ (0.1)	50	63:37
8[d]	CsOPiv (0.1)	41	63:37
9[d]	NHPI (0.1)	46	62:38
10[d]	NH_4F (1.0)	43	63:37
11[e]	PhCOONa (1.0)	48	58:42
12[e]	Na_2CO_3 (1.0)	38	61:39
13[e]	NaOPiv (1.0)	24	63:37
14[e]	NaOTf (1.0)	32	57:43
15[e]	NaOTFA (1.0)	40	63:37
16[e]	NaOPO (1.0)	35	61:39
17[e]	KPF_4 (1.0)	44	60:40
18[e]	dppp (0.2)	< 5	\
19[e]	$P(OEt)_3$ (1.0)	12	\

[a]Reaction conditions: **1a** (0.40 mmol), $TMSN_3$ (0.80 mmol), CuBr (0.08 mmol), pyridine (0.80 mmol), and **Additives** in PhCl (2.0 mL) was stirred at 90 °C for 48 h. [b]Isolated yield. [c]Determined by ^1H NMR measurement of the crude mixture. [d]PhCl : 1,4-dioxane (10 : 1) was used instead of PhCl. [e]0.2 mmol scale, NMR yield.

Table S6 Screening of the additives with addition of NaOAc[a]

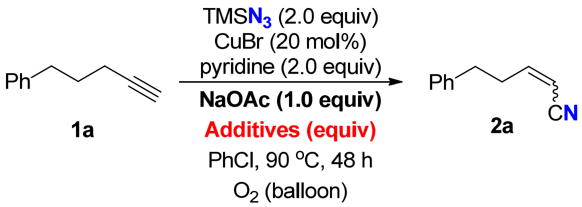

Entry	Additives (equiv)	Yield of **2a** (%)[b]	Z/E[c]
1[d]	NH_4I (1.0) or NaI (1.0)	0	/
2[d]	KBr (1.0)	66	62:38
3[d]	NaBr (1.0)	69	61:39
4	$NaBO_3$ H_2O (0.1)	71	65:35
5	dppf (0.1)	58	63:37
6	$FeCl_2$ (0.1)	56	65:35
7	$NaClO_4$ (1)	50	66:34
8	NH_4F (2)	47	62:38
9	DMF (10)	82	65:35
10	MS 4A (200 mg)	63	66:34
11	$Sc(OTf)_3$ (0.1)	56	65:35
12	$Mg(OTf)_2$ (0.1)	70	67:33
13	$InCl_3$ (0.1)	60	64:36
14	NH_4Br (0.5)	66	63:37
15	H_2O (10)	44	69:31
16	TBAB (0.1)	37	67:33
17	$NaHSO_3$ (0.1)	73	64:36
18	PhCOOK (0.1)	76	64:36
19	KPF_6 (0.1)	77	65:35
20	NH_4SCN (0.1)	23	60:40

[a]Reaction conditions: **1a** (0.40 mmol), $TMSN_3$ (0.80 mmol), CuBr (0.08 mmol), pyridine (0.80 mmol), NaOAc (0.4 mmol) and **Additives** in PhCl (2.0 mL) was stirred at 90 °C for 48 h. [b]Isolated yield. [c]Determined by ^1H NMR measurement of the crude mixture. [d]0.2 mmol scale, NMR yield.

Table S7 Screening of the catalysts[a]

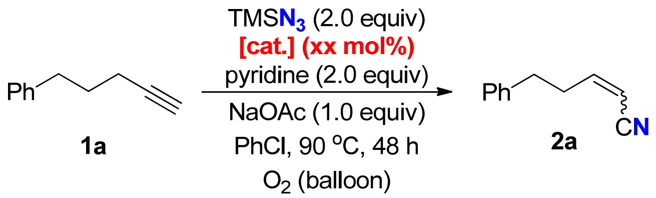

Entry	[Cat.] (xx mol%)	Yield of **2a** (%)[b]	*Z/E*[c]
1	none	0	-
**2**	**CuBr (20 mol%)**	**78**	**65:35**
3	CuOTf (20 mol%)	70	64:36
4	Cu_2O (20 mol%)	51	65:35
5	$Cu(OAc)_2$ (20 mol%)	49	69:31
6	$CuBr_2$ (20 mol%)	62	67:33
7	$Cu(ClO_4)_2•6H_2O$ (20 mol%)	42	71:29
8	CuBr (10 mol%)	41	63:37
9	CuOAc	60	67:33
10[d]	$FeCl_2$ (10 mol%)	0	-
11[d]	$FeCl_3$ (10 mol%)	0	-
12[d]	$CoCl_2$	0	-
13[d]	$Co(acac)_2$	0	-
14[d]	$Mn(OAc)•2H_2O$ (10 mol%)	0	-
15[d]	MnO_2	0	-
16[d]	$AgNO_3$	0	-

[a]Reaction conditions: **1a** (0.40 mmol), $TMSN_3$ (0.80 mmol), **catalyst**, pyridine (0.80 mmol), and NaOAc (0.40 mmol) in PhCl (2.0 mL) was stirred at 90 °C for 48 h. [b]Isolated yield. [c]Determined by ^1H NMR measurement of the crude mixture. [d]without NaOAc.

Table S8 Screening of the reaction temperatures[a]

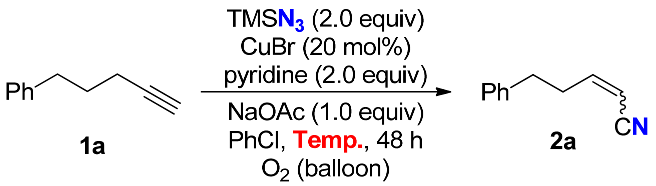

Entry	Temp.	Yield of **2a** (%)[b]	*Z/E*[c]
1	70 °C	67	63:37
**2**	**90 °C**	**78**	**65:35**
3	120 °C	31	69:31

[a]Reaction conditions: **1a** (0.40 mmol), $TMSN_3$ (0.80 mmol), CuBr (0.08 mmol), pyridine (0.80 mmol), and NaOAc (0.40 mmol) in PhCl (2.0 mL) was stirred at **Temp.** °C for 48 h. [b]Isolated yield. [c]Determined by ^1H NMR measurement of the crude mixture.

Table S9 Screening of the solvents[a]

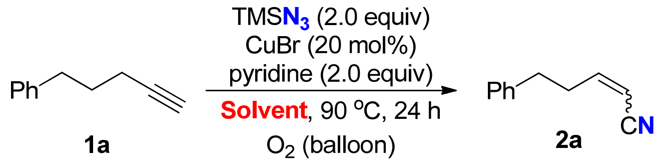

Entry	Solvent	Yield of **2a** (%)[b]	*Z/E*[c]
1	PhCl	45	/
2	$PhCH_3$	30	/
3	1,1,2-trichloroethane	43	/
4	1,2-dichlorobenzene	39	/
5	DMF	40	/
6	PhOMe	40	/
7	DMAc	37	/
8	DMSO	15	/
9	cumene	25	/
10[d]	THF	0	/
11[e]	$PhCH_3$	68	1.73:1
12[e]	1,1,2,2-tetrachloroethane	60	1.63:1
13[e]	PhOMe	65	1.60:1
14[e]	MeCN	< 10	/
15[e]	dioxane	0	/
16[e]	AcOPr	68	1.71:1
17[e]	PhCl	78	1.86:1

[a]Reaction conditions: **1a** (0.40 mmol), $TMSN_3$ (0.80 mmol), CuBr (0.08 mmol), pyridine (0.80 mmol), and NaOAc (0.40 mmol) in solvent (2.0 mL) was stirred at 90 °C for 24 h. [b]Isolated yield. [c]Not Determined. [d]Under reflux conditions. [e]0.2 mmol scale, NMR yield.

Table S10 Evaluation of isomerization reaction-1[a]

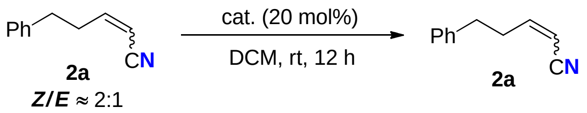

Entry	Cat. (20 mol%)	Recovery of **2a** (%)	*Z/E* Selectivity
1	PPh_3	> 95	NO improvement
2	P^nBu_3	> 95	**from 2.1:1 to 1:1.14**
3	PCy_3	> 95	NO improvement
4[b]	PMe_3 (THF)	> 95	**form 2.1:1 to 1:1.06**
5	DABCO	> 85	NO improvement
6	DBU	> 70	**from 2.1:1 to 1:1.18**
7	P^tBu_3	> 95	NO improvement
8	HMPA	> 99	NO improvement
9	R-(+)-Binap	> 80	NO improvement
10	IPr	> 50	NO improvement
11[b]	$ClRuH(CO)(PPh_3)_3$	> 95	NO improvement
12[c]	$ClRuH(PPh_3)_3$	> 95	NO improvement
13[c]	$Pd(TFA)_2/^iPrOH$	> 95	No improvement

[a]Reaction conditions: 2**a** (0.20 mmol), catalyst (0.04 mmol) in DCM (1.0 mL) was stirred at rt for 12 h. recovery and *Z/E* ratio was determined by NMR measurement. [b]PhCH3, rt, 60 °C or 80 °C [c] MeCN, 80 °C.

Table S11 Evaluation of isomerization reaction-2[a]

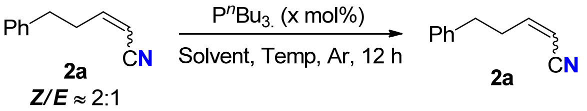

Entry	P^nBu_3 (x mol%)	Temp	Solvent	Additive	Recovery of **2a** (%)	*Z/E* Selectivity
1	20 mol%	rt	DCM	/	> 95	**from 2.1:1 to 1:1.14**
2	20 mol%	80 °C	$PhCH_3$	/	> 95	**from 1.23:1 to 1:19**
3	20 mol%	100 °C	$PhCH_3$	/	> 95	**from 1.23:1 to 1:1.22**
4	20 mol%	140 °C	MeCN	/	> 95	**from 1.23:1 to 1:1.20**
5	50 mol%	80 °C	MeCN	/	> 95	**from 1.09:1 to 1:1.14**
6	100 mol%	80 °C	MeCN	/	> 95	**from 1.09:1 to 1:1.07**
7	20 mol%	rt	DCM	HOAc	> 95	NO improvement
8	20 mol%	rt	DCM	NaOAc	> 99	**from 1.37:1 to 1:1.14**
9	20 mol%	rt	DCM	$BF_3\ Et_2O$	> 95	NO improvement
10	20 mol%	rt	EA	/	> 95	**from 2.10:1 to 1.48:1**
11	20 mol%	rt	MeCN	/	> 95	**from 2.10:1 to 1:1**
12	20 mol%	rt	MeOH	/	> 50	**from 2.10:1 to 1:1**
13	20 mol%	rt	hexane	/	> 95	**from 2.10:1 to 1.43:1**
14	20 mol%	rt	PhCl	/	> 95	**from 2.10:1 to 1.64:1**
15	20 mol%	rt	DMF	/	> 95	**from 2.10:1 to 1.05:1**

[a]Reaction conditions: 2**a** (0.20 mmol), P^nBu_3 (0.04 mmol) in solvent (1.0 mL) was stirred under Ar for 12 h. Recovery and *Z/E* ratio was determined by NMR measurement.

Experimental Procedure and Characterization Data for Products

01) 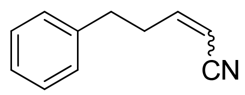

5-Phenylpent-2-enenitrile (2a):[1]

Typical procedure: Mix pent-4-yn-1-ylbenzene **1a** (57.7 mg, 0.40 mmol), $TMSN_3$ (92.2 mg, 0.80 mmol), CuBr (11.5 mg, 0.08 mmol), Py (63.2 mg, 0.80 mmol) and NaOAc (32.8 mg, 0.40 mmol) in PhCl (2.0 mL) under O_2 (balloon). The reaction mixture was stirred at 90 °C for 48 hours. After cooling down to room temperature and concentrating in vacuum, the residue was purified by flash chromatography on a short silica gel (eluent: petroleum ether/ethyl acetate = 50:1) to afford 49.3mg (78%) of **2a**. **2a**: obtained as a 65:35 mixture of *Z/E* isomers. Colorless liquid.

^{1}H NMR ($CDCl_3$, 400 MHz): δ = 7.35-7.25 (m, 2H), 7.24-7.12 (m, 3H), 6.70 (dt, J_1 = 16.4 Hz, J_2 = 7.0 Hz, 1H, *E*-isomer), 6.46 (dt, J_1 = 10.8 Hz, J_2 = 5.4 Hz, 1H, *Z*-isomer), 5.33-5.26 (m, 1H), 2.83-2.70 (m, 2H), 2.83-2.70 (m, 2H, *Z*-isomer), 2.57-2.49 (m, 2H, *E*-isomer); **^{13}C NMR ($CDCl_3$, 100 MHz)**: δ = 154.6, 153.7, 139.8, 139.7, 128.6, 128.5, 128.3, 128.2, 126.40, 126.35, 117.3, 115.7, 100.4, 100.1, 34.8, 34.2, 33.8, 33.2 ppm; **MS (70 eV) m/z (%)**: found 157.1 (M^+)

02) 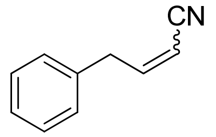

4-Phenylbut-2-enenitrile (2b):[2]

The reaction of but-3-yn-1-ylbenzene **1b** (52.1 mg, 0.40 mmol), $TMSN_3$ (92.2 mg, 0.80 mmol), CuBr (11.5 mg, 0.08 mmol), Py (63.2 mg, 0.80 mmol) and NaOAc (32.8 mg, 0.40 mmol) in PhCl (2.0 mL) under O_2 at 90 °C for 48 hours, afforded 25.0 mg (44%) of **2b**. **2b**: obtained as a 67:33 mixture of *Z/E* isomers. Colorless liquid.

***Z*-isomer: ^{1}H NMR ($CDCl_3$, 400 MHz)**: δ = 7.36-7.30 (m, 2H), 7.29-7.23 (m, 1H), 7.23-7.18 (m, 2H), 6.61 (dt, J_1 = 10.8 Hz, J_2 = 5.4 Hz, 1H), 5.41 (dt, J_1 = 10.8 Hz, J_2 = 1.4 Hz, 1H), 3.75 (d, J = 7.6 Hz, 2H); **^{13}C NMR ($CDCl_3$, 100 MHz)**: δ = 152.8,

136.8, 128.9, 128.5, 127.0, 115.9, 99.9, 38.0 ppm;

^{1}H NMR for the minor *E*-isomer: 7.37-7.30 (m, 2H), 7.29-7.22 (m, 1H), 7.16-7.12 (m, 2H), 6.87 (dt, J_1 = 16.0 Hz, J_2 = 6.6 Hz, 1H), 5.27 (dt, J_1 = 16.4 Hz, J_2 = 1.8 Hz, 1H), 3.53 (dd, J_1 = 6.4 Hz, J_2 = 1.6 Hz, 1H).

MS (70 eV) m/z (%): found 143.1 (M^+).

03) 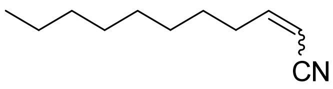

Undec-2-enenitrile (2c):[3]

The reaction of undec-1-yne **1c** (60.9 mg, 0.40 mmol), $TMSN_3$ (92.2 mg, 0.80 mmol), CuBr (11.5 mg, 0.08 mmol), Py (63.2 mg, 0.80 mmol) and NaOAc (32.8 mg, 0.40 mmol) in PhCl (2.0 mL) under O_2 at 90 °C for 48 hours, afforded 50.0 mg (76%) of **2c**. **2c**: obtained as a 61:39 mixture of *Z/E* isomers. Colorless liquid.

^{1}H NMR ($CDCl_3$, 400 MHz): δ = 6.72 (dt, J_1 = 16.4 Hz, J_2 = 7.0 Hz, 1H, *E*-isomer), 6.49 (dt, J_1 = 10.8 Hz, J_2 = 5.6 Hz, 1H, *Z*-isomer), 5.37-5.27 (m, 1H), 2.42 (td, J_1 = 7.5 Hz, J_2 = 7.5 Hz, 2H, *Z*-isomer), 2.26-2.15 (m, 2H, *E*-isomer), 1.53-1.40 (m, 2H), 1.35-1.21 (m, 10H), 0.88 (t, *J* = 6.6 Hz, 3H); **^{13}C NMR ($CDCl_3$, 100 MHz)**: δ = 156.2, 155.3, 117.6, 116.0, 99.5, 99.3, 33.3, 31.8, 31.7, 29.2, 29.08, 29.06, 29.0, 28.9, 28.2, 27.5, 22.6, 14.0 ppm;

MS (70 eV) m/z (%): found 150.1 ($[M-CH_3]^+$).

04)

Dec-2-enenitrile (2d):[4]

The reaction of dec-1-yne **1d** (55.3 mg, 0.40 mmol), $TMSN_3$ (92.2 mg, 0.80 mmol), CuBr (11.5 mg, 0.08 mmol), Py (63.2 mg, 0.80 mmol) and NaOAc (32.8 mg, 0.40 mmol) in PhCl (2.0 mL) under O_2 at 90 °C for 48 hours, afforded 37.7 mg (62%) of **2d**. **2d**: obtained as a 66:34 mixture of *Z/E* isomers. Colorless liquid.

^{1}H NMR ($CDCl_3$, 400 MHz): δ = 6.72 (dt, J_1 = 16.4 Hz, J_2 = 7.0 Hz, 1H, *E*-isomer),

6.48 (dt, J_1 = 10.8 Hz, J_2 = 5.4 Hz, 1H, *Z*-isomer), 5.35-5.28 (m, 1H), 2.46-2.39 (m, 2H, *Z*-isomer), 2.26-2.19 (m, 2H, *E*-isomer), 1.52-1.40 (m, 2H), 1.35-1.23 (m, 8H), 0.89 (t, J = 6.8 Hz, 3H); **^{13}C NMR ($CDCl_3$, 100 MHz)**: δ = 156.2, 155.3, 117.6, 116.1, 99.6, 99.4, 33.3, 31.9, 31.7, 31.6, 29.0, 28.92, 28.88, 28.2, 27.6, 22.6, 14.0 ppm;
MS (70 eV) m/z (%): found 136.1 ($[M-CH_3]^+$).

05) 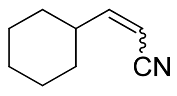

3-Cyclohexylacrylonitrile (2e):[5]
The reaction of prop-2-yn-1-ylcyclohexane **1e** (48.9 mg, 0.40 mmol), $TMSN_3$ (92.2 mg, 0.80 mmol), CuBr (11.5 mg, 0.08 mmol), Py (63.2 mg, 0.80 mmol) and NaOAc (32.8 mg, 0.40 mmol) in PhCl (2.0 mL) under O_2 at 90 °C for 48 hours, afforded 34.3 mg (63%) of **2e**. **2e**: obtained as a 60:40 mixture of *Z/E* isomers. Colorless liquid.
^{1}H NMR ($CDCl_3$, 400 MHz): δ = 6.67 (dd, J_1 = 16.4 Hz, J_2 = 6.4 Hz, 1H, *E*-isomer), 6.32 (dd, J_1 = 10.8 Hz, J_2 = 10.4 Hz, 1H, *Z*-isomer), 5.27 (dd, J_1 = 16.4 Hz, J_2 = 1.6 Hz, 1H, *E*-isomer), 5.21 (dd, J_1 = 10.8 Hz, J_2 = 0.4 Hz, 1H, *Z*-isomer), 2.68-2.55 (m, 1H, *Z*-isomer), 2.20-2.09 (m, 1H, *E*-isomer), 1.80-1.65 (m, 5H), 1.42-1.08 (m, 5H); **^{13}C NMR ($CDCl_3$, 100 MHz)**: δ = 160.8, 160.1, 117.9, 116.1, 97.5, 97.1, 41.4, 41.0, 31.7, 31.1, 25.6, 25.5, 25.4, 25.1 ppm;
MS (70 eV) m/z (%): found 135.1 (M^+)

06) 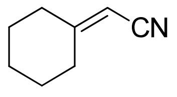

2-Cyclohexylideneacetonitrile (2f):[6]
The reaction of ethynylcyclohexane **1f** (43.3 mg, 0.40 mmol), $TMSN_3$ (92.2 mg, 0.80 mmol), CuBr (11.5 mg, 0.08 mmol), Py (63.2 mg, 0.80 mmol) and NaOAc (32.8 mg, 0.40 mmol) in PhCl (2.0 mL) under O_2 at 90 °C for 48 hours, afforded 29.8 mg (61%) of **2f**. Colorless liquid.
^{1}H NMR ($CDCl_3$, 400 MHz): δ = 5.04 (s, 1H), 2.49 (t, J = 6.0 Hz, 2H), 2.25 (t, J =

5.6 Hz, 2H), 1.71-1.58 (m, 6H); **^{13}C NMR ($CDCl_3$, 100 MHz)**: δ = 168.7, 117.0, 91.9, 35.9, 33.1, 27.9, 27.5, 25.6 ppm;

MS (70 eV) m/z (%): found 121.1 (M^+)

07) 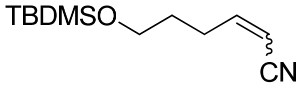

6-((*tert*-Butyldimethylsilyl)oxy)hex-2-enenitrile (2g):

The reaction of *tert*-butyl(hex-5-yn-1-yloxy)dimethylsilane **1g** (85.0 mg, 0.40 mmol), $TMSN_3$ (92.2 mg, 0.80 mmol), CuBr (11.5 mg, 0.08 mmol), Py (63.2 mg, 0.80 mmol) and NaOAc (32.8 mg, 0.40 mmol) in PhCl (2.0 mL) under O_2 at 90 °C for 48 hours, afforded 61.0 mg (68%) of **2g**. **2g**: obtained as a 69:31 mixture of *Z/E* isomers. Colorless liquid.

^{1}H NMR ($CDCl_3$, 400 MHz): δ = 6.74 (dt, J_1 = 16.4 Hz, J_2 = 7.0 Hz, 1H, *E*-isomer), 6.52 (dt, J_1 = 10.8 Hz, J_2 = 5.6 Hz, 1H, *Z*-isomer), 5.33 (dt, J_1 = 16.4 Hz, J_2 = 1.6 Hz, 1H, *E*-isomer), 5.31 (dt, J_1 = 10.8 Hz, J_2 = 1.4 Hz, 1H, *Z*-isomer), 3.66-3.58 (m, 2H), 2.49 (tdd, J_1 = 7.6 Hz, J_2 = 7.6 Hz, J_3 = 1.2 Hz, 2H, *Z*-isomer), 2.30 (tdd, J_1 = 7.2 Hz, J_2 = 7.2 Hz, J_3 = 1.2 Hz, 2H, *E*-isomer), 1.72-1.61 (m, 2H), 0.879 (s, 9H, *Z*-isomer), 0.875 (s, 9H, *E*-isomer), 0.04 (s, 6H, *Z*-isomer), 0.03 (m, 6H, *E*-isomer); **^{13}C NMR ($CDCl_3$, 100 MHz)**: δ = 155.8, 155.0, 117.4, 115.9, 99.8, 99.4, 62.0, 61.7, 31.3, 30.6, 29.9, 28.6, 25.83, 25.80, 18.2, -5.5 ppm;

HRMS *m/z* (ESI) calcd for $C_{12}H_{23}NNaOSi$ $(M + Na)^+$ 248.1441, found 248.1440.

IR (neat): ν_{max} = 2953, 2933, 2859, 2222, 1628, 1105, 839.

08) 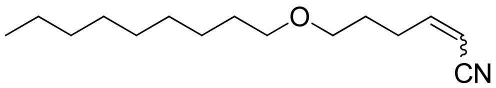

6-(Nonyloxy)hex-2-enenitrile (2h):

The reaction of 1-(hex-5-yn-1-yloxy)nonane **1h** (89.8 mg, 0.40 mmol), $TMSN_3$ (92.2 mg, 0.80 mmol), CuBr (11.5 mg, 0.08 mmol), Py (63.2 mg, 0.80 mmol) and NaOAc

(32.8 mg, 0.40 mmol) in PhCl (2.0 mL) under O_2 at 90 °C for 48 hours, afforded 55.9 mg (59%) of **2h**. **2h**: obtained as a 69:31 mixture of *Z/E* isomers. Colorless liquid.

^{1}H NMR ($CDCl_3$, 400 MHz): δ = 6.74 (dt, J_1 = 16.4 Hz, J_2 = 7.0 Hz, 1H, *E*-isomer), 6.53 (dt, J_1 = 10.8 Hz, J_2 = 5.6 Hz, 1H, *Z*-isomer), 5.35 (dt, J_1 = 16.4 Hz, J_2 = 1.8 Hz, 1H, *E*-isomer), 5.32 (dt, J_1 = 10.8 Hz, J_2 = 1.4 Hz, 1H, *Z*-isomer), 3.46-3.56 (m, 4H), 2.49 (tdd, J_1 = 7.4 Hz, J_2 = 7.4 Hz, J_3 = 1.2 Hz, 2H, *Z*-isomer), 2.30 (tdd, J_1 = 7.2 Hz, J_2 = 7.2 Hz, J_3 = 1.2 Hz, 2H, *E*-isomer), 1.80-1.70 (m, 2H), 1.60-1.50 (m, 2H), 1.38-1.21 (m, 12H), 0.88 (t, *J* = 6.8 Hz, 3H); **^{13}C NMR ($CDCl_3$, 100 MHz)**: δ = 155.5, 154.8, 117.4, 115.9, 99.9, 99.6, 71.13, 71.08, 69.6, 69.2, 31.8, 30.2, 29.6, 29.5, 29.4, 29.2, 28.9, 28.3, 27.8, 26.1, 22.6, 14.0 ppm;

HRMS *m/z* (ESI) calcd for $C_{15}H_{27}NNaO$ $(M + Na)^+$ 260.1985, found 260.1982.

IR (neat): ν_{max} = 2927, 2856, 2222, 1632, 1465, 1117, 739.

09) 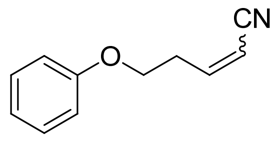

Methyl 10-cyanodec-9-enoate (2i):

The reaction of (pent-4-yn-1-yloxy)benzene **1i** (64.1 mg, 0.40 mmol), $TMSN_3$ (92.2 mg, 0.80 mmol), CuBr (11.5 mg, 0.08 mmol), Py (63.2 mg, 0.80 mmol) and NaOAc (32.8 mg, 0.40 mmol) in PhCl (2.0 mL) under O_2 at 90 °C for 48 hours, afforded 41.6 mg (60%) of **2i**. **2i**: obtained as a 68:32 mixture of *Z/E* isomers. Colorless liquid.

^{1}H NMR ($CDCl_3$, 400 MHz): δ = 7.33-7.25 (m, 2H), 7.00-6.91 (m, 1H), 6.91-6.85 (m, 2H), 6.79 (dt, J_1 = 16.4 Hz, J_2 = 6.8 Hz, 1H, *E*-isomer), 6.66 (dt, J_1 = 11.2 Hz, J_2 = 5.6 Hz, 1H, *Z*-isomer), 5.47 (dt, J_1 = 16.4 Hz, J_2 = 1.6 Hz, 1H, *E*-isomer), 5.43 (dt, J_1 = 11.2 Hz, J_2 = 1.4 Hz, 1H, *Z*-isomer), 4.10-4.02 (m, 2H), 2.93-2.85 (m, 2H, *Z*-isomer), 2.67 (tdd, J_1 = 6.4 Hz, J_2 = 6.4 Hz, J_3 = 1.6 Hz, 2H, *E*-isomer); **^{13}C NMR ($CDCl_3$, 100 MHz)**: δ = 158.5, 158.3, 151.9, 151.1, 129.62, 129.59, 121.3, 121.2, 117.2, 115.8, 114.6, 102.0, 101.6, 65.6, 65.2, 33.1, 31.8 ppm;

HRMS *m/z* (EI) calcd for $C_{12}H_{13}NNaO_2$ $(M + Na)^+$ 226.0839, found 226.0835.

IR (neat): ν_{max} = 3070, 2925, 2879, 2222, 1634, 1600, 1496, 1244, 1042, 755.

10) 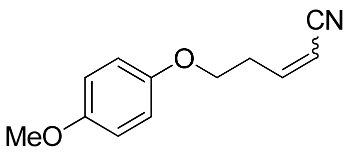

5-(4-Methoxyphenoxy)pent-2-enenitrile (2j):

The reaction of 1-methoxy-4-(pent-4-yn-1-yloxy)benzene **1j** (76.1 mg, 0.40 mmol), $TMSN_3$ (92.2 mg, 0.80 mmol), CuBr (11.5 mg, 0.08 mmol), Py (63.2 mg, 0.80 mmol) and NaOAc (32.8 mg, 0.40 mmol) in PhCl (2.0 mL) under O_2 at 90 °C for 48 hours, afforded 41.0 mg (50%) of **2j**. **2j**: obtained as a 69:31 mixture of *Z/E* isomers. Colorless liquid.

***Z*-isomer: ^{1}H NMR ($CDCl_3$, 400 MHz)**: δ = 6.85-6.82 (m, 4H), 6.67 (dt, J_1 = 10.8 Hz, J_2 = 5.6 Hz, 1H), 5.45 (dt, J_1 = 11.2 Hz, J_2 = 1.4 Hz, 1H), 4.04 (t, J = 6.0 Hz, 2H), 3.77 (s, 3H), 2.91-2.84 (m, 2H); **^{13}C NMR ($CDCl_3$, 100 MHz)**: δ = 154.1, 152.5, 151.2, 115.7, 115.5, 114.7, 101.5, 66.3, 55.7, 31.9 ppm;

^{1}H NMR for the minor *E*-isomer: 6.85-6.76 (m, 5H), 5.48 (dt, J_1 = 16.4 Hz, J_2 = 1.6 Hz, 1H), 4.05-3.89 (m, 2H), 3.77 (s, 3H), 2.70-2.62 (m, 2H).

HRMS *m/z* (ESI) calcd for $C_{12}H_{13}NNaO_2$ $(M + Na)^+$ 226.0839, found 226.0835.

IR (neat): ν_{max} = 3070, 2953, 2220, 1624, 1509, 1232, 1042, 828.

11) 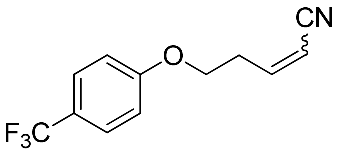

6-(4-(Trifluoromethyl)phenoxy)hex-2-enenitrile (2k):

The reaction of 1-(hex-5-yn-1-yloxy)-4-(trifluoromethyl)benzene **1k** (91.3 mg, 0.40 mmol), $TMSN_3$ (92.2 mg, 0.80 mmol), CuBr (11.5 mg, 0.08 mmol), Py (63.2 mg, 0.80 mmol) and NaOAc (32.8 mg, 0.40 mmol) in PhCl (2.0 mL) under O_2 at 90 °C for 48 hours, afforded 63.8 mg (66%) of **2k**. **2k**: obtained as a 63:37 mixture of *Z/E* isomers. Colorless liquid.

***Z*-isomer: ^{1}H NMR ($CDCl_3$, 400 MHz)**: δ = 7.55 (d, J = 8.8 Hz, 2H), 6.96 (d, J = 8.4 Hz, 2H), 6.66 (dt, J_1 = 10.8 Hz, J_2 = 5.6 Hz, 1H), 5.49 (dt, J_1 = 10.8 Hz, J_2 = 1.2 Hz, 1H), 4.14 (t, J = 6.0 Hz, 2H), 2.93 (tdd, J_1 = 6.0 Hz, J_2 = 6.0 Hz, J_3 = 1.2 Hz, 2H); **^{13}C NMR ($CDCl_3$, 100 MHz)**: δ = 160.8, 150.4, 126.9 (q, J = 3.7 Hz), 124.3 (q, J = 269.5

Hz), 123.3 (q, J = 32.4 Hz), 115.6, 114.4, 102.1, 65.8, 31.5 ppm;

^{1}H NMR for the minor E-isomer: 7.58-7.54 (m, 2H), 6.98-6.92 (m, 2H), 6.81 (dt, J_1 = 16.4 Hz, J_2 = 6.8 Hz, 1H). 5.54-5.46 (m, 1H), 4.17-4.07 (m, 2H), 2.77-2.70 (m, 2H).

HRMS *m/z* (ESI) calcd for $C_{12}H_{10}F_3NNaO$ $(M + Na)^+$ 264.0607, found 264.0601.

IR (neat): ν_{max} = 3075, 2938, 2225, 1617, 1331, 1258, 1113, 839.

12) 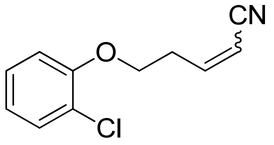

5-(2-Chlorophenoxy)pent-2-enenitrile (2l):

The reaction of 1-chloro-2-(pent-4-yn-1-yloxy)benzene **1l** (77.9 mg, 0.40 mmol), $TMSN_3$ (92.2 mg, 0.80 mmol), CuBr (11.5 mg, 0.08 mmol), Py (63.2 mg, 0.80 mmol) and NaOAc (32.8 mg, 0.40 mmol) in PhCl (2.0 mL) under O_2 at 90 °C for 48 hours, afforded 58.6 mg (71%) of **2l**. **2l**: obtained as a 36:34 mixture of *Z/E* isomers. Colorless liquid.

***Z*-isomer: ^{1}H NMR ($CDCl_3$, 400 MHz)**: δ = 7.39-7.35 (m, 1H), 7.21 (td, J_1 = 8.0 Hz, J_2 = 1.6 Hz, 1H), 6.96-6.88 (m, 2H), 6.76 (dt, J_1 = 11.2 Hz, J_2 = 5.4 Hz, 1H), 5.48 (d, J = 11.2 Hz, 1H), 4.15 (t, J = 5.8 Hz, 2H), 2.99-2.92 (tdd, J_1 = 6.4 Hz, J_2 = 6.4 Hz, J_3 = 1.2 Hz, 2H); **^{13}C NMR ($CDCl_3$, 100 MHz)**: δ = 153.8, 150.7, 130.3, 127.7, 123.1, 121.9, 115.6, 113.6, 101.7, 66.8, 31.6 ppm;

^{1}H NMR for the minor E-isomer: 7.37 (dd, J_1 = 8.0 Hz, J_1 = 1.2 Hz, 1H), 7.22 (td, J_1 = 8.0 Hz, J_2 = 1.6 Hz, 1H), 6.96-6.86 (m, 2H), 6.86 (dt, J_1 = 11.2 Hz, J_2 = 7.0 Hz, 1H), 5.55 (d, J = 16.4 Hz, 1H), 4.13 (t, J = 6.0 Hz, 2H), 2.75 (tdd, J_1 = 7.6 Hz, J_2 = 7.6 Hz, J_3 = 1.6 Hz, 2H).

HRMS *m/z* (ESI) calcd for $C_{11}H_{10}ClNNaO$ $(M + Na)^+$ 230.0343, found 230.0339.

IR (neat): ν_{max} = 3066, 2934, 2225, 1636, 1250, 1063, 751.

13) 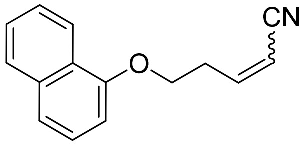

5-(Naphthalen-2-yloxy)pent-2-enenitrile (2m):

The reaction of 1-(pent-4-yn-1-yloxy)naphthalene **1m** (84.1 mg, 0.40 mmol), $TMSN_3$ (92.2 mg, 0.80 mmol), CuBr (11.5 mg, 0.08 mmol), Py (63.2 mg, 0.80 mmol) and NaOAc (32.8 mg, 0.40 mmol) in PhCl (2.0 mL) under O_2 at 90 °C for 48 hours, afforded 54.0 mg (60%) of **2m**. **2m**: obtained as a 64:36 mixture of *Z/E* isomers. Colorless liquid. ***Z*-isomer: ^{1}H NMR ($CDCl_3$, 400 MHz)**: δ = 8.27-8.21 (m, 1H), 7.82-7.76 (m, 1H), 7.53-7.42 (m, 3H), 7.36 (t, *J* = 8.0 Hz, 1H), 6.77 (d, *J* = 7.6 Hz, 1H), 6.74 (dt, J_1 = 10.8 Hz, J_2 = 5.4 Hz, 1H), 5.49 (dt, J_1 = 10.8 Hz, J_2 = 1.2 Hz, 1H), 4.25 (t, *J* = 6.0 Hz, 2H), 3.04 (tdd, J_1 = 6.4 Hz, J_2 = 6.4 Hz, J_3 = 1.2 Hz, 2H); **^{13}C NMR ($CDCl_3$, 100 MHz)**: δ = 154.1, 151.1, 134.4, 127.5, 126.5, 125.7, 125.40, 125.35, 121.8, 120.7, 115.8, 104.6, 101.8, 65.7, 31.9 ppm;

^{1}H NMR for the minor *E*-isomer: 8.20-8.15 (m, 1H), 7.82-7.76 (m, 1H), 7.52-7.41 (m, 3H), 7.35 (t, *J* = 7.8 Hz, 1H), 6.87 (dt, J_1 = 16.4 Hz, J_2 = 6.8 Hz, 1H), 6.78-6.72 (m, 1H), 5.56-5.44 (m, 1H), 4.25-4.17 (m, 2H), 2.79 (tdd, J_1 = 7.2 Hz, J_2 = 7.2 Hz, J_3 = 1.6 Hz, 2H);

HRMS *m/z* (ESI) calcd for $C_{15}H_{13}NNaO$ $(M + Na)^+$ 246.0889, found 246.0885.

IR (neat): ν_{max} = 3056, 2931, 2880, 2220, 1626, 1508, 1270, 1101, 773.

14) 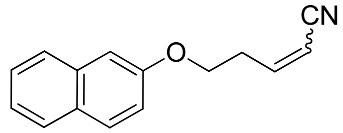

5-(Naphthalen-2-yloxy)pent-2-enenitrile (2n):

The reaction of 2-(pent-4-yn-1-yloxy)naphthalene **1n** (84.1 mg, 0.40 mmol), $TMSN_3$ (92.2 mg, 0.80 mmol), CuBr (11.5 mg, 0.08 mmol), Py (63.2 mg, 0.80 mmol) and NaOAc (32.8 mg, 0.40 mmol) in PhCl (2.0 mL) under O_2 at 90 °C for 48 hours, afforded 51.3 mg (57%) of **2n**. **2n**: obtained as a 64:36 mixture of *Z/E* isomers. Colorless liquid. ***Z*-isomer: ^{1}H NMR ($CDCl_3$, 400 MHz)**: δ = 7.80-7.70 (m, 3H), 7.48-7.41 (m, 1H), 7.38-7.31 (m, 1H), 7.17-7.11 (m, 2H), 6.71 (dt, J_1 = 10.8 Hz, J_2 = 5.4 Hz, 1H), 5.47 (dt, J_1 = 10.8 Hz, J_2 = 1.4 Hz, 1H), 4.20 (t, *J* = 6.2 Hz, 2H), 2.99-2.93 (m, 2H); **^{13}C NMR ($CDCl_3$, 100 MHz)**: δ = 156.4, 151.0, 134.4, 129.5, 129.1, 127.7, 126.7, 126.5, 123.8, 118.7, 115.7, 106.7, 101.7, 65.6, 31.7 ppm;

^{1}H NMR for the minor *E*-isomer: 7.80-7.70 (m, 3H), 7.48-7.41 (m, 1H), 7.38-7.31 (m, 1H), 7.17-7.08 (m, 2H), 6.82 (dt, J_1 = 16.4 Hz, J_2 = 6.8 Hz, 1H), 5.52 (dt, J_1 = 16.4 Hz, J_2 = 1.6 Hz, 1H), 4.20-4.12 (m, 2H), 2.75-2.68 (m, 2H).

HRMS *m/z* (ESI) calcd for $C_{15}H_{13}NNaO$ $(M + Na)^+$ 246.0889, found 246.0884.

IR (neat): ν_{max} = 3059, 2945, 2220, 1628, 1508, 1250, 1144, 837, 753.

15) 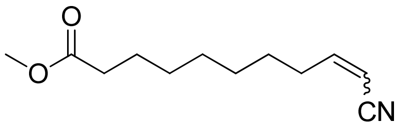

Methyl 10-cyanodec-9-enoate (2o):

The reaction of methyl undec-10-ynoate **1o** (78.5 mg, 0.40 mmol), $TMSN_3$ (92.2 mg, 0.80 mmol), CuBr (11.5 mg, 0.08 mmol), Py (63.2 mg, 0.80 mmol) and NaOAc (32.8 mg, 0.40 mmol) in PhCl (2.0 mL) under O_2 at 90 °C for 48 hours, afforded 58.0 mg (69%) of **2o**. **2o**: obtained as a 69:31 mixture of *Z/E* isomers. Colorless liquid.

^{1}H NMR ($CDCl_3$, 400 MHz): δ = 6.72 (dt, J_1 = 16.4 Hz, J_2 = 7.0 Hz, 1H, *E*-isomer), 6.49 (dt, J_1 = 11.2 Hz, J_2 = 5.4 Hz, 1H, *Z*-isomer), 5.37-5.29 (m, 1H), 3.67 (s, 3H), 2.42 (td, J_1 = 7.4 Hz, J_2 = 7.4 Hz, 2H, *Z*-isomer), 2.31 (t, *J* = 7.4 Hz, 2H), 2.26-2.18 (m, 2H, *E*-isomer), 1.66-1.58 (m, 2H), 1.52-1.42 (m, 2H), 1.37-1.28 (m, 6H); **^{13}C NMR ($CDCl_3$, 100 MHz)**: δ = 174.1, 174.0, 155.9, 155.0, 117.4, 115.9, 99.6, 99.4, 51.3, 33.9, 33.8, 33.1, 31.7, 28.79, 18.78, 28.75, 28.63, 28.60, 28.0, 27.4, 24.69, 24.67, 24.67 ppm;

HRMS *m/z* (ESI) calcd for $C_{12}H_{19}NNaO_2$ $(M + Na)^+$ 232.1308, found 232.1305.

IR (neat): ν_{max} = 2933, 2858, 2221, 1738, 1631, 1249, 1173, 743.

16) 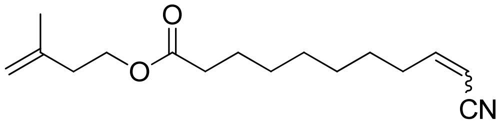

3-Methylbut-3-en-1-yl 10-cyanodec-9-enoate (2p):

Mix 3-methylbut-3-en-1-yl undec-10-ynoate **1p** (100.2 mg, 0.40 mmol), $TMSN_3$ (69.2 mg, 0.60 mmol), CuBr (11.5 mg, 0.08 mmol), Py (63.2 mg, 0.80 mmol) and NaOAc (32.8 mg, 0.40 mmol) in PhCl (2.0 mL) under O_2. After stirring at 90 °C for 24 hours, another portion of $TMSN_3$ (69.2 mg, 0.60 mmol) was added. Then the mixture was

stirred for another 24 hours. After cooling down to room temperature and concentrating in vacuum, the residue was purified by flash chromatography on a short silica gel to afford 41.7 mg (40%) of **2p**. **2p**: obtained as a 64:36 mixture of *Z/E* isomers. Colorless liquid.

^{1}H NMR ($CDCl_3$, 400 MHz): δ = 6.72 (dt, J_1 = 16.4 Hz, J_2 = 6.8 Hz, 1H, *E*-isomer), 6.48 (dt, J_1 = 11.2 Hz, J_2 = 5.4 Hz, 1H, *Z*-isomer), 5.38-5.29 (m, 1H), 4.80 (s, 1H), 4.73 (s, 1H), 4.19 (t, *J* = 6.8 Hz, 2H), 2.46-2.39 (m, 2H, *Z*-isomer), 2.34 (t, *J* = 6.8 Hz, 2H), 2.29 (t, *J* = 7.4 Hz, 2H), 2.25-2.18 (m, 2H, *E*-isomer), 1.76 (s, 3H), 1.65-1.58 (m, 2H), 1.52-1.42 (m, 2H), 1.38-1.28 (m, 6H); **^{13}C NMR ($CDCl_3$, 100 MHz)**: δ = 173.73, 173.68, 156.0, 155.1, 141.7, 117.5, 116.0, 112.2, 99.7, 99.5, 62.4, 36.7, 34.21, 34.18, 33.2, 31.8, 28.87, 28.86, 28.8, 28.7, 28.1, 27.5, 24.81, 24.79, 22.4 ppm;

HRMS *m/z* (ESI) calcd for $C_{16}H_{25}NNaO_2$ $(M + Na)^+$ 286.1778, found 286.1778.

IR (neat): ν_{max} = 3077, 2933, 2858, 2221, 1735, 1651, 1632, 1457, 1176, 836.

17) 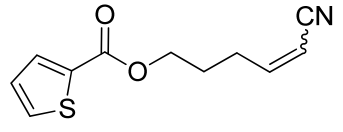

5-Cyanopent-4-en-1-yl thiophene-2-carboxylate (2q):

The reaction of hex-5-yn-1-yl thiophene-2-carboxylate **1q** (83.3 mg, 0.40 mmol), $TMSN_3$ (92.2 mg, 0.80 mmol), CuBr (11.5 mg, 0.08 mmol), Py (63.2 mg, 0.80 mmol) and NaOAc (32.8 mg, 0.40 mmol) in PhCl (2.0 mL) under O_2 (ballon) at 90 °C for 60 hours, afforded 65.0 mg (73%) of **2q**. **2q**: obtained as a 34:66 mixture of *Z/E* isomers. Colorless liquid.

^{1}H NMR ($CDCl_3$, 400 MHz): δ = 7.76-7.71 (m, 1H), 7.52-7.47 (m, 1H), 7.06-7.01 (m, 1H), 6.68 (dt, J_1 = 16.4 Hz, J_2 = 6.8 Hz, 1H, *E*-isomer), 6.46 (dt, J_1 = 10.8 Hz, J_2 = 5.4 Hz, 1H, *Z*-isomer), 5.36-5.28 (m, 1H), 4.29-4.22 (m, 2H), 2.56-2.48 (m, 2H, *Z*-isomer), 2.36-2.28 (m, 2H, *E*-isomer), 192-1.80 (m, 2H); **^{13}C NMR ($CDCl_3$, 100 MHz)**: δ = 162.0, 161.9, 154.3, 153.4, 133.5, 133.4, 133.3, 132.53, 133.47, 127.8, 127.7, 117.1, 115.6, 100.6, 100.5, 63.72, 63.67, 29.9, 28.4, 27.3, 26.8 ppm;

HRMS *m/z* (ESI) calcd for $C_{11}H_{11}NNaO_2S$ $(M + Na)^+$ 244.0403, found 244.0398.

IR (neat): ν_{max} = 3104, 2958, 2222, 1709, 1633, 1262, 1098, 751.

18) 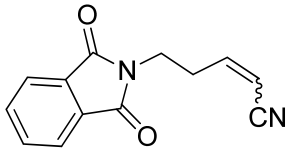

5-(1,3-Dioxoisoindolin-2-yl)pent-2-enenitrile (2r):

The reaction of 2-(pent-4-yn-1-yl)isoindoline-1,3-dione **1r** (87.9 mg, 0.40 mmol), $TMSN_3$ (92.2 mg, 0.80 mmol), CuBr (11.5 mg, 0.08 mmol), Py (63.2 mg, 0.80 mmol) and NaOAc (32.8 mg, 0.40 mmol) in PhCl (2.0 mL) under O_2 (ballon) at 90 °C for 72 hours, afforded 58.9 mg (65%) of **2r**. **2r**: obtained as a 69:31 mixture of *Z/E* isomers. Colorless liquid.

^{1}H NMR ($CDCl_3$, 400 MHz): δ = 7.89-7.84 (m, 2H), 7.78-7.72 (m, 2H), 6.70 (dt, J_1 = 16.4 Hz, J_2 = 7.2 Hz, 1H, *E*-isomer), 6.53 (dt, J_1 = 11.2 Hz, J_2 = 5.6 Hz, 1H, *Z*-isomer), 5.46-5.36 (m, 1H), 3.92-3.81 (m, 2H), 2.88-2.80 (m, 2H, *Z*-isomer), 2.69-2.61 (m, 2H, *E*-isomer); **^{13}C NMR ($CDCl_3$, 100 MHz)**: δ = 168.0, 167.9, 151.1, 150.4, 134.14, 134.08, 131.72, 131.65, 123.32, 123.30, 116.7, 115.2, 102.3, 102.0, 35.8, 35.7, 32.2, 31.1 ppm;

HRMS *m/z* (ESI) calcd for $C_{13}H_{10}N_2NaO_2$ $(M + Na)^+$ 249.0635, found 249.0630.

IR (neat): ν_{max} = 3064, 2939, 2220, 1773, 1708, 1619, 1397, 722.

19) 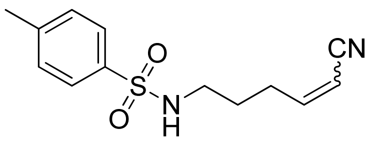

***N*-(5-Cyanopent-4-en-1-yl)-4-methylbenzenesulfonamide (2s):**

The reaction of N-(hex-5-yn-1-yl)-4-methylbenzenesulfonamide **1s** (100.5 mg, 0.40 mmol), $TMSN_3$ (92.2 mg, 0.80 mmol), CuBr (11.5 mg, 0.08 mmol), Py (63.2 mg, 0.80 mmol) and NaOAc (32.8 mg, 0.40 mmol) in PhCl (2.0 mL) under O_2 (ballon) at 90 °C for 48 hours, afforded 64.5 mg (61%) of **2s**. **2s**: obtained as a 70:30 mixture of *Z/E* isomers. Colorless liquid.

^{1}H NMR ($CDCl_3$, 400 MHz): δ = 7.77-7.73 (m, 2H), 7.34-7.30 (m, 2H), 6.60 (dt, J_1 = 16.0 Hz, J_2 = 7.0 Hz, 1H, *E*-isomer), 6.44 (dt, J_1 = 10.8 Hz, J_2 = 5.4 Hz, 1H,

Z-isomer), 5.36-5.26 (m, 1H), 5.12-4.96 (m, 1H), 3.00-2.90 (m, 2H), 2.46-2.39 (s, 2H, *Z*-isomer), 2.44 (s, 3H), 2.30-2.22 (m, 2H, *E*-isomer), 1.72-1.58 (m, 2H); **^{13}C NMR ($CDCl_3$, 100 MHz)**: δ = 154.2, 153.5, 143.7, 143.6, 136.7, 129.8, 127.0, 117.2, 115.7, 100.7, 100.5, 42.3, 42.0, 29.9, 28.8, 28.2, 27.6, 21.5 ppm;

HRMS *m/z* (ESI) calcd for $C_{13}H_{16}N_2NaO_2S$ $(M + Na)^+$, 287.0825, found 287.0818.

IR (neat): ν_{max} = 3280, 3068, 2929, 2872, 2222, 1624, 1326, 1159.

20) 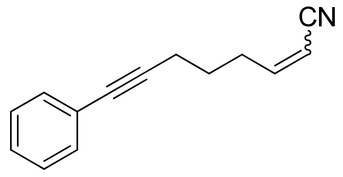

8-Phenyloct-2-en-7-ynenitrile (2t):

The reaction of octa-1,7-diyn-1-ylbenzene **1t** (72.9 mg, 0.40 mmol), $TMSN_3$ (92.2 mg, 0.80 mmol), CuBr (11.5 mg, 0.08 mmol), Py (63.2 mg, 0.80 mmol) and NaOAc (32.8 mg, 0.40 mmol) in PhCl (2.0 mL) under O_2 at 90 °C for 72 hours, afforded 35.6 mg (46%) of **2t**. **2t**: obtained as a 66:34 mixture of *Z/E* isomers. Colorless liquid.

^{1}H NMR ($CDCl_3$, 400 MHz): δ = 7.44-7.35 (m, 2H), 7.31-7.26 (m, 3H), 6.75 (dt, J_1 = 16.0 Hz, J_2 = 7.0 Hz, 1H, *E*-isomer), 6.53 (dt, J_1 = 11.2 Hz, J_2 = 5.6 Hz, 1H, *Z*-isomer), 5.39 (dt, J_1 = 16.0 Hz, J_2 = 1.6 Hz, 1H, *E*-isomer), 5.36 (dt, J_1 = 11.2 Hz, J_2 = 1.2 Hz, 1H, *Z*-isomer), 2.62 (tdd, J_1 = 7.6 Hz, J_2 = 7.6 Hz, J_3 = 1.2 Hz, 2H, *Z*-isomer), 2.50-2.43 (m, 2H), 2.45-2.38 (m, 2H, *E*-isomer), 1.84-1.71 (m, 2H); **^{13}C NMR ($CDCl_3$, 100 MHz)**: δ = 154.8, 153.9, 131.52, 131.49, 128.23, 128.19, 127.8, 127.7, 123.6, 123.5, 117.3, 115.8, 100.5, 100.3, 88.6, 88.4, 81.7, 81.6, 32.2, 30.9, 27.3, 26.5, 18.9, 18.7 ppm;

HRMS *m/z* (ESI) calcd for $C_{14}H_{13}KN$ $(M + K)^+$, 234.0680, found 234.0679.

IR (neat): ν_{max} = 3057, 2931, 2863, 2237, 2222, 1626, 1490, 758, 693.

21) 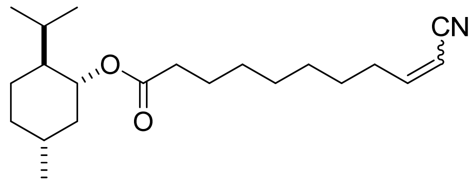

(1*R*,2*S*,5*R*)-2-Isopropyl-5-methylcyclohexyl 10-cyanodec-9-enoate (4a):

The reaction of (1*R*,2*S*,5*R*)-2-isopropyl-5-methylcyclohexyl undec-10-ynoate **3a** (128.2 mg, 0.40 mmol), $TMSN_3$ (92.2 mg, 0.80 mmol), CuBr (11.5 mg, 0.08 mmol), Py (63.2 mg, 0.80 mmol) and NaOAc (32.8 mg, 0.40 mmol) in PhCl (2.0 mL) under O_2 at 90 °C for 48 hours, afforded 65.3 mg (49%) of **4a**. **4a**: obtained as a 65:35 mixture of *Z/E* isomers. Colorless liquid.

^{1}H NMR ($CDCl_3$, 400 MHz): δ = 6.71 (dt, J_1 = 16.0 Hz, J_2 = 7.0 Hz, 1H, *E*-isomer), 6.48 (dt, J_1 = 11.2 Hz, J_2 = 5.4 Hz, 1H, *Z*-isomer), 5.35-5.28 (m, 1H), 4.68 (td, J_1 = 11.0 Hz, J_2 = 4.4 Hz, 1H), 2.46-2.38 (m, 2H, *Z*-isomer), 2.28 (t, *J* = 7.4 Hz, 2H), 2.25-2.18 (m, 2H, *E*-isomer), 2.01-1.94 (m, 1H), 1.91-1.82 (m, 1H), 1.72-1.57 (m, 4H), 1.52-1.28 (m, 10H), 1.12-0.83 (m, 3H), 0.90 (d, *J* = 6.4 Hz, 3H), 0.89 (d, *J* = 7.2 Hz, 3H), 0.76 (d, *J* = 7.2 Hz, 3H); **^{13}C NMR ($CDCl_3$, 100 MHz)**: δ = 173.31, 173.27, 156.0, 155.1, 117.5, 116.0, 99.7, 99.5, 73.91, 73.89, 47.0, 40.9, 34.9, 34.63, 34.59, 34.2, 33.2, 31.8, 31.3, 28.89, 28.88, 28.86, 28.8, 28.7, 28.1, 27.5, 26.2, 25.0, 24.9, 23.4, 22.0, 20.7, 16.3 ppm;

HRMS *m/z* (ESI) calcd for $C_{21}H_{35}NNaO_2$ $(M + Na)^+$, 356.2560, found 356.2557.
IR (neat): ν_{max} = 2930, 2861, 2222, 1729, 1629, 1459, 1181.

22) 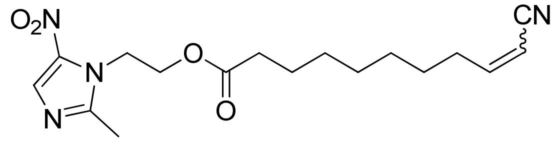

2-(2-Methyl-5-nitro-1*H*-imidazol-1-yl)ethyl 10-cyanodec-9-enoate (4b):

The reaction of 2-(2-methyl-5-nitro-1*H*-imidazol-1-yl)ethyl undec-10-ynoate **3b** (134.2 mg, 0.40 mmol), $TMSN_3$ (92.2 mg, 0.80 mmol), CuBr (11.5 mg, 0.08 mmol), Py (63.2 mg, 0.80 mmol) and NaOAc (32.8 mg, 0.40 mmol) in PhCl (2.0 mL) under O_2 at 90 °C for 48 hours, afforded 84.1 mg (60%) of **4b**. **4b**: obtained as a 68:32 mixture of *Z/E* isomers. Colorless liquid.

^{1}H NMR ($CDCl_3$, 400 MHz): δ = 7.95 (s, 1H), 6.72 (dt, J_1 = 16.4 Hz, J_2 = 7.0 Hz, 1H, *E*-isomer), 6.48 (dt, J_1 = 11.2 Hz, J_2 = 5.4 Hz, 1H, *Z*-isomer), 5.36-5.29 (m, 1H), 4.60 (t, *J* = 5.2 Hz, 2H), 4.41 (t, *J* = 5.2 Hz, 2H), 2.52 (s, 3H), 2.46-2.38 (m, 2H, *Z*-isomer), 2.29-2.24 (m, 2H), 2.24-2.18 (m, 2H, *E*-isomer), 1.60-1.51 (m, 2H), 1.50-1.40 (m, 2H), 1.36-1.23 (m, 6H); **^{13}C NMR ($CDCl_3$, 100 MHz)**: δ = 172.93, 172.89, 155.9,

155.0, 150.8, 150.7, 138.5, 133.03, 133.01, 117.5, 115.0, 99.7, 99.5, 62.2, 45.0, 44.9, 33.7, 33.2, 31.7, 28.8, 28.73, 28.71, 28.6, 28.0, 27.4, 24.5, 14.3 ppm;

HRMS *m/z* (ESI) calcd for $C_{17}H_{25}N_4O_4$ $(M + H)^+$ 349.1870, found 349.1868.

IR (neat): ν_{max} = 2932, 2858, 2220, 1740, 1630, 1468, 1190.

23) 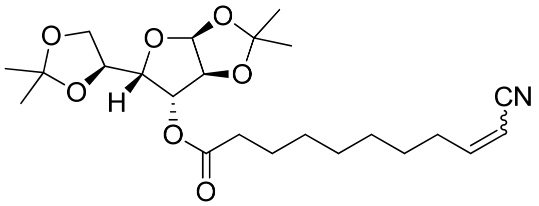

(3*aS*,5*S*,6*R*,6*aS*)-5-((*S*)-2,2-Dimethyl-1,3-dioxolan-4-yl)-2,2-dimethyltetrahydrofuro[2,3-*d*][1,3]dioxol-6-yl 10-cyanodec-9-enoate (4c):

The reaction of (3*aS*,5*S*,6*R*,6*aS*)-5-((*S*)-2,2-dimethyl-1,3-dioxolan-4-yl)-2,2-dimethyltetrahydrofuro[2,3-*d*][1,3]dioxol-6-yl undec-10-ynoate **3c** (169.8 mg, 0.40 mmol), $TMSN_3$ (92.2 mg, 0.80 mmol), CuBr (11.5 mg, 0.08 mmol), Py (63.2 mg, 0.80 mmol) and NaOAc (32.8 mg, 0.40 mmol) in PhCl (2.0 mL) under O_2 at 90 °C for 48 hours, afforded 116.9 mg (67%) of **4c**. **4c**: obtained as a 67:33 mixture of *Z/E* isomers. Colorless liquid.

^{1}H NMR ($CDCl_3$, 400 MHz): δ = 6.71 (dt, J_1 = 16.4 Hz, J_2 = 7.0 Hz, 1H, *E*-isomer), 6.48 (dt, J_1 = 10.8 Hz, J_2 = 5.4 Hz, 1H, *Z*-isomer), 5.89-5.86 (m, 1H), 5.36-5.30 (m, 1H), 5.27 (d, J_1 = 1.6 Hz, 1H), 4.49-4.46 (m, 1H), 4.21-4.19 (m, 2H), 4.11-3.98 (m, 2H), 2.46-2.38 (m, 2H, *Z*-isomer), 2.35 (td, J_1 = 7.8 Hz, J_2 = 2.0 Hz, 2H), 2.25-2.18 (m, 2H, *E*-isomer), 1.67-1.59 (m, 2H), 1.52 (s, 3H), 1.50-1.41 (m, 2H), 1.41 (s, 3H), 1.36-1.28 (m, 12H); **^{13}C NMR ($CDCl_3$, 100 MHz)**: δ = 172.19, 172.16, 155.9, 155.0, 117.5, 116.0, 112.22, 112.20, 109.2, 105.0, 99.7, 99.6, 83.3, 79.8, 75.80, 75.78, 72.4, 67.2, 34.1, 33.2, 31.7, 28.84, 28.80, 28.76, 28.7, 28.0, 27.5, 26.74, 26.66, 26.1, 25.2, 24.7 ppm;

HRMS *m/z* (ESI) calcd for $C_{23}H_{35}NNaO_7$ $(M + Na)^+$, 460.2306, found 460.2295.

IR (neat): ν_{max} = 2988, 2935, 2859, 2221, 1745, 1631, 1377, 1077, 847.

24) 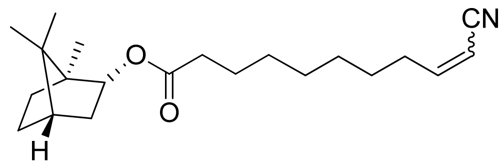

(1*S*,2*R*,4*S*)-1,7,7-Trimethylbicyclo[2.2.1]heptan-2-yl 10-cyanodec-9-enoate (4d):

The reaction of (1*S*,2*R*,4*S*)-1,7,7-trimethylbicyclo[2.2.1]heptan-2-yl undec-10-ynoate **3d** (127.4 mg, 0.40 mmol), $TMSN_3$ (92.2 mg, 0.80 mmol), CuBr (11.5 mg, 0.08 mmol), Py (63.2 mg, 0.80 mmol) and NaOAc (32.8 mg, 0.40 mmol) in PhCl (2.0 mL) under O_2 at 90 °C for 48 hours, afforded 97.3 mg (73%) of **4d**. **4d**: obtained as a 65:35 mixture of *Z/E* isomers. Colorless liquid.

^{1}H NMR ($CDCl_3$, 400 MHz): δ = 6.71 (dt, J_1 = 16.4 Hz, J_2 = 7.0 Hz, 1H, *E*-isomer), 6.48 (dt, J_1 = 10.8 Hz, J_2 = 5.4 Hz, 1H, *Z*-isomer), 5.36-5.28 (m, 1H), 4.92-4.85 (m, 1H), 2.46-2.39 (m, 2H, *Z*-isomer), 2.38-2.28 (m, 3H), 2.25-2.18 (m, 2H, *E*-isomer), 1.98-1.90 (m, 1H), 1.80-1.70 (m, 1H), 1.69-1.60 (m, 3H), 1.52-1.41 (m, 2H), 1.38-1.18 (m, 8H), 0.95 (dd, J_1 = 13.6 Hz, J_2 = 3.6 Hz, 1H), 0.91 (s, 3H), 0.87 (s, 3H), 0.83 (s, 3H); **^{13}C NMR ($CDCl_3$, 100 MHz)**: δ = 174.1, 174.0, 156.0, 155.1, 117.5, 116.0, 99.7, 99.5, 79.60, 79.57, 48.7, 47.7, 44.8, 36.8, 34.60, 34.56, 33.2, 31.8, 28.90, 28.88, 28.80, 28.7, 28.1, 28.0, 27.5, 27.1, 25.0, 19.7, 18.8, 13.5 ppm;

HRMS *m/z* (ESI) calcd for $C_{21}H_{33}NNaO_2$ $(M + Na)^+$ 354.2404, found 354.2403.

IR (neat): ν_{max} = 2933, 2860, 2221, 1731, 1631, 1455, 1186.

25) 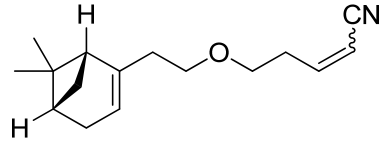

5-(2-((1*R*,5*S*)-6,6-dimethylbicyclo[3.1.1]hept-2-en-2-yl)ethoxy)pent-2-enenitrile (4e):

The reaction of (1*R*,5*S*)-6,6-dimethyl-2-(2-(pent-4-yn-1-yloxy)ethyl)bicyclo[3.1.1]hept-2-ene **3e** (92.9 mg, 0.40 mmol), $TMSN_3$ (92.2 mg, 0.80 mmol), CuBr (11.5 mg, 0.08 mmol), Py (63.2 mg, 0.80 mmol) and NaOAc (32.8 mg, 0.40 mmol) in PhCl (2.0 mL) under O_2 at 90 °C for 48 hours, afforded 50.2 mg (52%) of **4e**. **4e**: obtained as a 63:37 mixture of

Z/E isomers. Colorless liquid.

***Z*-isomer: ^{1}H NMR (CDCl$_3$, 400 MHz)**: δ = 6.59 (dt, J_1 = 10.8 Hz, J_2 = 5.6 Hz, 1H), 5.38 (dt, J_1 = 10.8 Hz, J_2 = 1.4 Hz, 1H), 5.28-5.23 (m, 1H), 3.54 (t, J = 6.2 Hz, 2H), 3.47-3.40 (m, 2H), 2.72-2.64 (m, 2H), 2.35 (dt, J_1 = 8.4 Hz, J_2 = 4.2 Hz, 1H), 2.29-2.13 (m, 4H), 2.10-2.05 (m, 1H), 2.03 (td, J_1 = 5.6 Hz, J_2 = 1.6 Hz, 1H), 1.27 (s, 3H), 1.14 (d, J = 8.4 Hz, 1H), 0.82 (s, 3H); **^{13}C NMR (CDCl$_3$, 100 MHz)**: δ = 152.2, 144.9, 118.0, 115.9, 100.8, 69.4, 68.3, 45.8, 40.7, 38.0, 37.0, 32.3, 31.6, 31.3, 26.3, 21.1 ppm;

^{1}H NMR for the minor *E*-isomer: 6.75 (dt, J_1 = 16.4 Hz, J_2 = 6.8 Hz, 1H), 5.46-5.38 (m, 1H), 5.28-5.23 (m, 1H), 3.56-3.48 (m, 2H), 3.47-3.38 (m, 2H), 2.50-2.48 (m, 2H), 2.40-2.32 (m, 1H), 2.29-2.13 (m, 4H), 2.10-2.00 (m, 2H), 1.27 (s, 3H), 1.14 (d, J = 8.4 Hz, 1H), 0.82 (s, 3H).

HRMS *m/z* (ESI) calcd for $C_{16}H_{24}NO$ $(M + H)^+$ 246.1852, found 246.1845.

IR (neat): ν_{max} = 2918, 2873, 2223, 1726, 1632, 1367, 1114.

26) 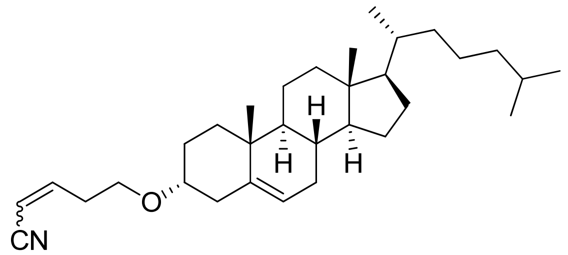

5-(((3*R*,8*S*,9*S*,10*R*,13*R*,14*S*,17*R*)-10,13-Dimethyl-17-((*R*)-6-methylheptan-2-yl)-2,3,4,7,8,9,10,11,12,13,14,15,16,17-tetradecahydro-1*H*-cyclopenta[*a*]phenanthren-3-yl)oxy)pent-2-enenitrile (4f):

The reaction of (3*R*,8*S*,9*S*,10*R*,13*R*,14*S*,17*R*)-10,13-dimethyl-17-((*R*)-6-methylheptan-2-yl)-3-(pent-4-yn-1-yloxy)-2,3,4,7,8,9,10,11,12,13,14,15,16,17-tetradecahydro-1*H*-cyclopenta[*a*]phenanthrene **3f** (90.6 mg, 0.20 mmol), TMSN$_3$ (46.1 mg, 0.40 mmol), CuBr (5.8 mg, 0.04 mmol), Py (31.6 mg, 0.40 mmol) and NaOAc (16.4 mg, 0.20 mmol) in PhCl (1.0 mL) under O_2 at 90 °C for 48 hours, afforded 46.7 mg (50%) of **4f**. **4f**: obtained as a 68:32 mixture of *Z/E* isomers. White solid.

^{1}H NMR (CDCl$_3$, 400 MHz): δ = 6.75 (dt, J_1 = 16.4 Hz, J_2 = 6.8 Hz, 1H, *E*-isomer), 6.48 (dt, J_1 = 11.2 Hz, J_2 = 5.4 Hz, 1H, *Z*-isomer), 5.46-5.36 (m, 1H), 5.36-5.32 (m, 1H), 3.61-3.54 (m, 2H), 3.20-3.08 (m, 1H), 2.70-2.63 (m, 2H, *Z*-isomer), 2.50-2.43 (m, 2H, *E*-isomer), 2.36-2.29 (m, 1H), 2.22-2.13 (m, 1H), 2.04-1.92 (m, 2H), 1.91-1.77 (m, 3H), 1.61-1.04 (m, 20H), 1.04-0.96 (m, 1H), 0.99 (s, 3H), 0.91 (d, *J* = 6.8 Hz, 3H), 0.860 (d, *J* = 6.4 Hz, 3H), 0.855 (d, J_1 = 6.8 Hz, 3H), 0.67 (s, 3H); **^{13}C NMR (CDCl$_3$, 100 MHz)**: δ = 153.0, 152.3, 140.7, 140.6, 121.8, 121.7, 117.4, 115.8, 101.2, 100.7, 79.4, 79.2, 65.7, 65.3, 56.7, 56.1, 50.2, 42.3, 39.7, 39.5, 39.02, 38.99, 37.2, 36.8, 36.2, 35.7, 34.0, 32.7, 31.91, 31.86, 28.35, 28.31, 28.2, 28.0, 24.2, 23.8, 22.8, 22.5, 21.0, 19.3, 18.7, 11.8 ppm;

HRMS *m/z* (ESI) calcd for $C_{32}H_{51}NNaO$ $(M + Na)^+$ 488.3863, found 488.3858.

IR (neat): ν_{max} = 2936, 2868, 2222, 1733, 1672, 1632, 1466, 1380, 1107.

Mechanistic Studies

(1) Control experiment with allene

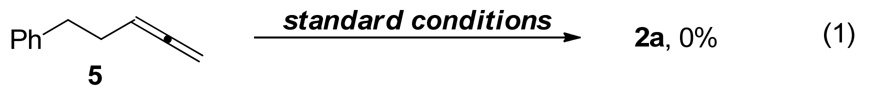

Allene **5** was prepared according to Ma's report.[7] Mix **5** (57.7 mg, 0.40 mmol), $TMSN_3$ (92.2 mg, 0.80 mmol), CuBr (11.5 mg, 0.08 mmol), Py (63.2 mg, 0.80 mmol) and NaOAc (32.8 mg, 0.40 mmol) in PhCl (2.0 mL) under O_2 (balloon). The reaction mixture was stirred at 90 °C for 48 hours. After cooling down to room temperature, the residue was analyzed by TLC and GC-MS. **NO 2a** was detected.

(2) Control experiment with propargylic azide

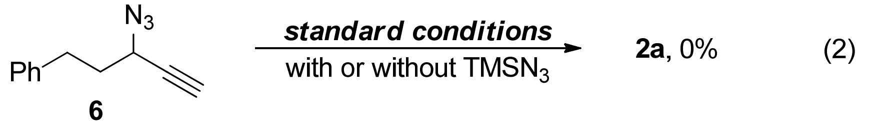

Propargylic azide **6** was prepared according to literature report.[8] Mix **6** (74.1 mg, 0.40 mmol), $TMSN_3$ (**0.80 mmol or 0 mmol**, **for two independent reactions**), CuBr (11.5 mg, 0.08 mmol), Py (63.2 mg, 0.80 mmol) and NaOAc (32.8 mg, 0.40 mmol) in PhCl (2.0 mL) under O_2 (balloon). The reaction mixture was stirred at 90 °C for 48 hours. After cooling down to room temperature, the residue was analyzed by TLC and GC-MS. **NO 2a** was detected.

(3) Control experiment with allyl azide

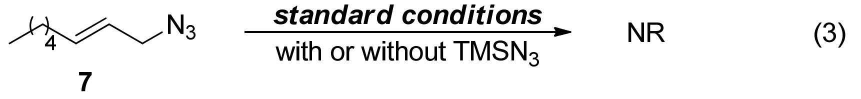

Allyl azide **7** was prepared according to literature report.[9] Mix **7** (61.3 mg, 0.40 mmol), $TMSN_3$ (**0.80 mmol or 0 mmol**, **for two independent reactions**), CuBr (11.5 mg, 0.08 mmol), Py (63.2 mg, 0.80 mmol) and NaOAc (32.8 mg, 0.40 mmol) in PhCl (2.0 mL) under O_2 (balloon). The reaction mixture was stirred at 90 °C for 48 hours. After cooling down to room temperature, the residue was analyzed by TLC and GC-MS. **NO 2a** was detected. And **7** remained.

(4) Preparation of copper(I)-acetylide

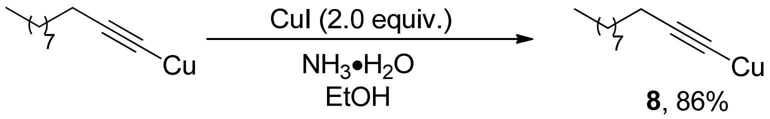

Copper(I)-acetylide **8** was prepared according to the reported procedure.[10] Add **undec-1-yne** (0.46 g, 3 mmol) to a solution of copper iodide (1.15 g, 6.0 mmol) in a mixture of ammonium hydroxide (28% NH_3 solution, 28 mL) and ethanol (9 mL) by dropwise under Ar atmosphere. The deep blue reaction mixture was stirred overnight at room temperature and the yellow precipitate was collected by filtration and successively washed with ammonium hydroxide (10% NH_3 solution, 3 x 15 mL), water (3 x 15 mL), ethanol (3 x 15 mL), and diethyl ether (3 x 15 mL). The bright yellow solid was then dried under high vacuum overnight to afford the desired Copper(I)-acetylide **8** (0.56 g, 86%).

(5) Control experiment with copper(I)-acetylide

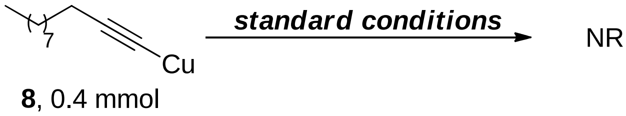

(4)

Mix **8** (85.9 mg, 0.40 mmol), $TMSN_3$ (92.2 mg, 0.80 mmol), CuBr (11.5 mg, 0.08 mmol), Py (63.2 mg, 0.80 mmol) and NaOAc (32.8 mg, 0.40 mmol) in PhCl (2.0 mL) under O_2 (balloon). The reaction mixture was stirred at 90 °C for 48 hours. After cooling down to room temperature, the residue was analyzed by TLC and GC-MS. **NO 2a** was detected.

(6) Reaction catalyzed by copper(I)-acetylide

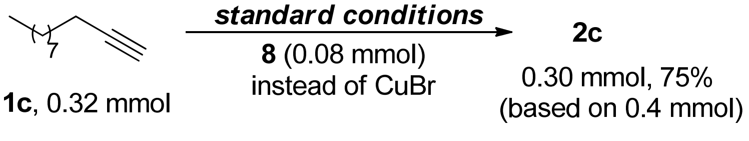

(5)

Mix **1c** (48.7 mg, 0.32 mmol), copper(I)-acetylide **8** (used as a **catalyst**, 17.2 mg, 0.08 mmol), $TMSN_3$ (92.2 mg, 0.80 mmol), **NO** CuBr (0 mmol), Py (63.2 mg, 0.80 mmol) and NaOAc (32.8 mg, 0.40 mmol) in PhCl (2.0 mL) under O_2 (balloon). The reaction mixture was stirred at 90 °C for 48 hours. After cooling down to room

temperature and concentrating in vacuum, the residue was purified by flash chromatography on a short silica gel (eluent: petroleum ether/ethyl acetate = 50:1) to afford 49.4 mg (75%, *Z/E* = 63:37) of **2c**. The yield is competent with the reaction employing CuBr (20 mol%) as a catalyst (entry 3 of Table 2 in Text).

(7) Preparation of **1a-1-*d*₁**

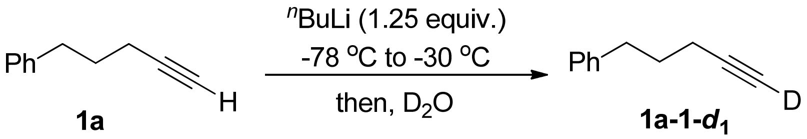

According to the literature[11], **1a** (0.58 g, 4 mmol) and dry THF (5 mL) was added to a sealed flask via a syringe under argon. ^{n}BuLi (2.5 M hexane solution; 2.0 mL, 1.25 equiv.) was added dropwise at -78 ˚C, and the reaction mixture was stirred at for 1 h at -30 ˚C. D_2O (99.9%-d; 10 mL) was carefully added to the lithium acetylide solution at -78 ˚C, and the reaction mixture was stirred for 30 min at the same temperature. After dilution with diethyl ether (15 mL), the mixture was washed with HCl (2 M, 10 mL) and extracted with diethyl ether (10 mL x 3). The combined organic phases were dried ($MgSO_4$) and concentrated in vacuo. **1a-1-d_1** was obtained by flash chromatography on a short silica gel (0.47g, 81%, > 99% D).

(8) Deuterium labelling experiments with **1a-1-d_1**

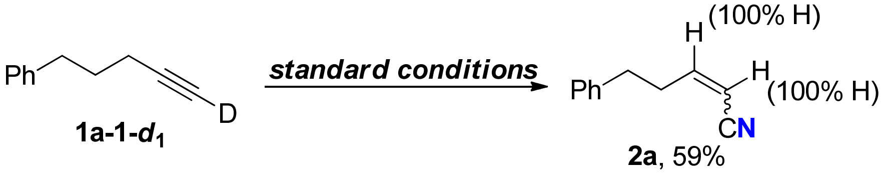

(6)

Mix **1a-1-d_1** (58.1 mg, 0.40 mmol), $TMSN_3$ (92.2 mg, 0.80 mmol), CuBr (11.5 mg, 0.08 mmol), Py (63.2 mg, 0.80 mmol) and NaOAc (32.8 mg, 0.40 mmol) in PhCl (2.0 mL) under O_2 (balloon). The reaction mixture was stirred at 90 °C for 48 hours. After cooling down to room temperature and concentrating in vacuum, the residue was purified by flash chromatography on a short silica gel (eluent: petroleum ether/ethyl acetate = 50:1) to afford 43.2 mg (69%, *Z/E* = 65:35) of **2a**. **NO** deuterium was detected in the product.

(9) Preparation of **1a-3,3-d_2**

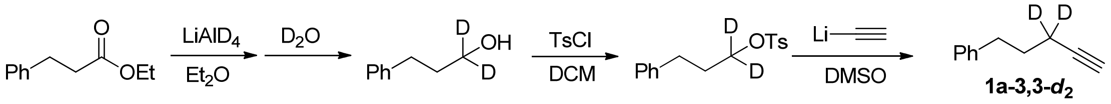

According to the reported procedure,[12] $LiAlD_4$ (0.42 g, 10 mmol) was added to a solution of **ethyl 3-phenylpropanoate** (1.78 g, 10 mmol, in 12 mL dry Et_2O) by dropwise under argon at room temperature. The mixture was stirred until the reaction was completed (TLC monitoring). After careful addition of D_2O to the reaction mixture, the white solid was filtered off and the solvent carefully evaporated to dryness and then the residue obtained was purified by flash chromatography on silica gel to give 1.32 g of the pure dideuterated alcohol (96% for the first step).

Next, pyridine (1.58 g, 20 mmol) was added dropwise to the dideuterated alcohol (1.32 g, 9.6 mmol) and tosyl chloride (2.86 g, 15 mmol) in CH_2Cl_2 at room temperature. After being stirred for 24 h at room temperature, the reaction mixture was poured into saturated NH_4Cl. Usual work up and column purification afforded 1,1-dideuterio hydrocinnamyl tosylate (2.39 g, 85% yield for the second step).

Then, a solution of 1,1-dideuterio hydrocinnamyl tosylate (1.96 g, 6.7 mmol) in DMSO (6 mL) was added dropwise to a solution of lithium acetylide ethylenediamine complex (0.78 g, 8.7 mmol) in DMSO (6 mL) at room temperature. The resulting dark brown solution was stirred for 3 h and then 30 mL of ice-water was added. After adjusting pH to 1 by HCl (1 M) and extracting with diethyl ether (10 mL x 3), The combined organic phases were dried ($MgSO_4$) and concentrated in vacuo. **1a-3,3-d_2** was obtained by flash chromatography on a short silica gel (0.10 g, 7% for three steps, > 99% D).

(10) Deuterium labelling experiments with **1a-3,3-d_2**

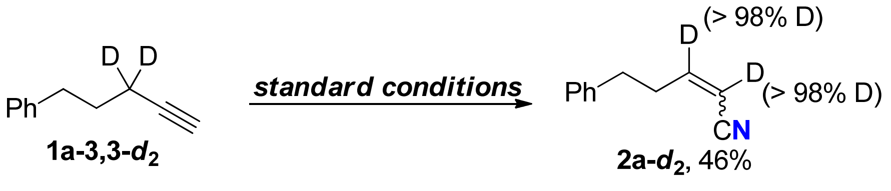

(7)

Mix **1a-3,3-d_2** (14.6 mg, 0.10 mmol), $TMSN_3$ (23.1 mg, 0.20 mmol), CuBr (2.9

mg, 0.02 mmol), Py (15.8 mg, 0.20 mmol) and NaOAc (8.2 mg, 0.10 mmol) in PhCl (0.5 mL) under O_2 (balloon). The reaction mixture was stirred at 90 °C for 48 hours. After cooling down to room temperature and concentrating in vacuum, the residue was purified by flash chromatography on a short silica gel (eluent: petroleum ether/ethyl acetate = 50:1) to afford 7.3 mg (46%, *Z/E* = 64:36) of **2a-*d*₂**. To our surprise, nearly 100% incorporation of deuterium at the both α and β positions of the nitrile was observed.

(11) Intermolecular kinetic isotopic experiment

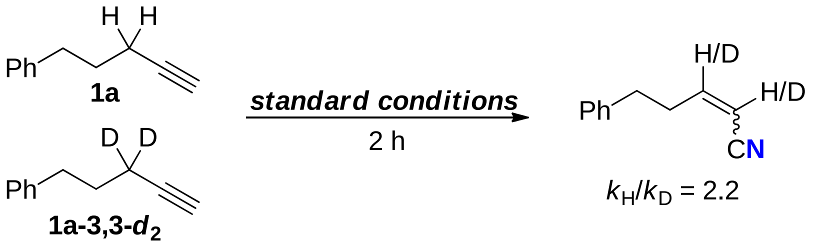

(8)

Mix **1a** (28.8 mg, 0.20 mmol), **1a-3,3-d_2** (29.2 mg, 0.20 mmol), $TMSN_3$ (92.2 mg, 0.80 mmol), CuBr (11.5 mg, 0.08 mmol), Py (63.2 mg, 0.80 mmol) and NaOAc (32.8 mg, 0.40 mmol) in PhCl (2.0 mL) under O_2 (balloon). The reaction mixture was stirred at 90 °C for **2 hours**. After cooling down to room temperature and concentrating in vacuum, the residue was analyzed by ^{1}H-NMR. The calulated k_H/k_D is 2.2.

Computational Methods

All the DFT calculations were carried out with the GAUSSIAN 09 series of programs.[13] Density functional theory B3LYP[14] with a standard 6-31G(d) basis set (SDD basis set for Cu atom)[15] was used for geometry optimizations (keyword 5D was used in the calculations). The vibrational frequencies were computed at the same level to check whether each optimized structure is an energy minimum or a transition state and to evaluate its zero-point vibrational energy (ZPVE) and thermal corrections at 298 K. IRC calculations[16] were used to confirm that the transition states found from the optimization calculations connect the related reactants and products. Single-point energies were obtained at the B3LYP level using 6-311+G(d,p) basic set (SDD basis set for Cu atom) based on the structures obtained on the gas-phase. Solvation energies were evaluated by a self-consistent reaction field (SCRF) using the CPCM model,[17] where UFF radii were used. The reported energies are Gibbs free energies in PhCl solution (ΔG_{sol}).

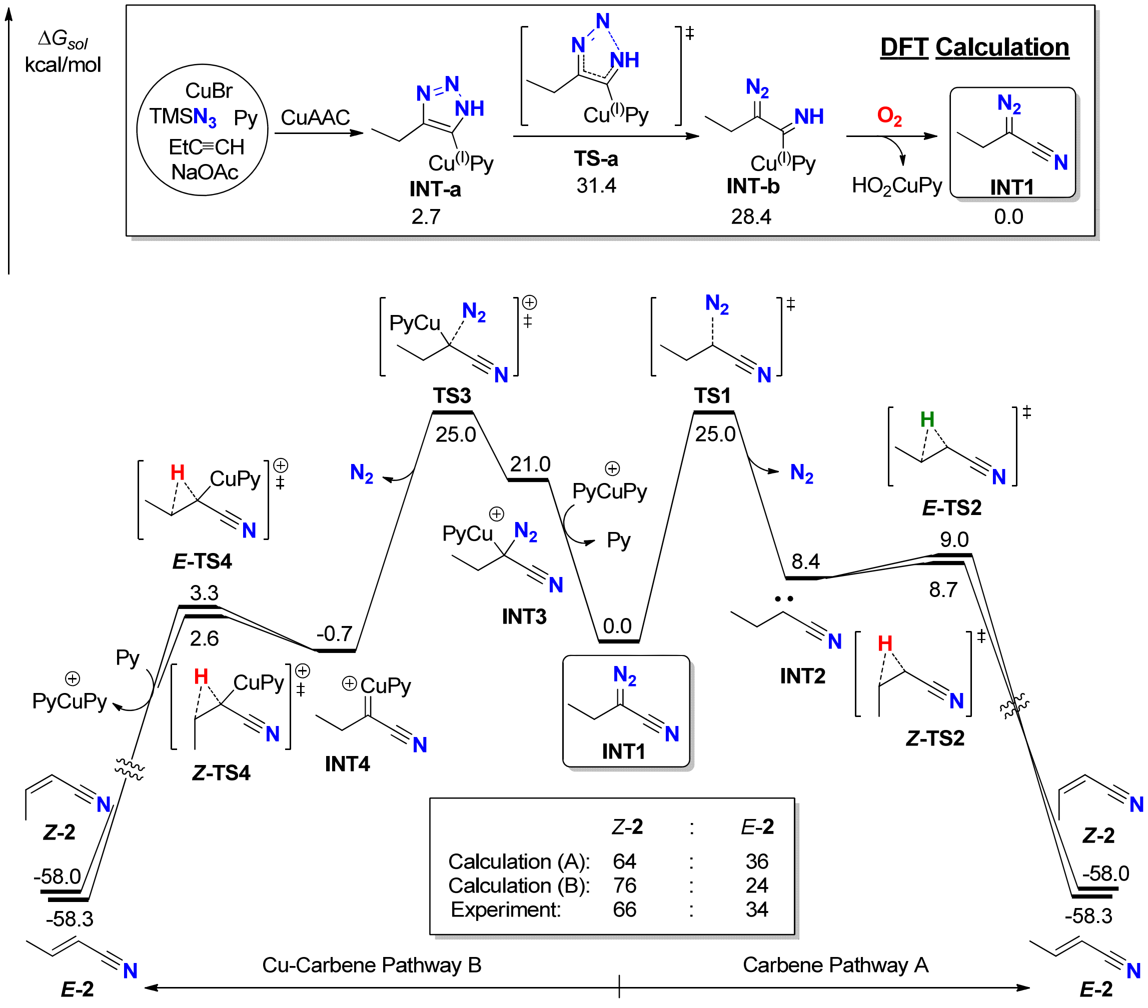

Figure S1 DFT-computed energy profiles.

To further unravel the mechanism, the density functional theory (DFT) calculation investigation was carried out (Figure S1). After the reasonable Cu-catalyzed azide-alkyne cycloaddition (CuAAC), the formed triazole intermediate **INT-a** undergoes ring-opening reaction through transition state **TS-a** with an activation free energy of 28.7 kcal/mol to afford **INT-b**. Although this is an endergonic process by 25.7 kcal/mol, the following oxidation of imine motif by molecular oxygen exergonic by 28.4 kcal/mol, delivering the stable α-diazonitrile **INT1**. See text for the details of the following processes.

Computed Energies of All Stationary Points

Table S12 Sum of electronic and zero-point energies (E, in a.u.), sum of electronic and thermal enthalpies (H, in a.u.), sum of electronic and thermal free energies (G, in a.u.), thermal correction to Gibbs free energy (TCGFE, in Hartree), and total free energy in solution (E_S, in Hartree, solvent = PhCl).

Structure	*E*[a]	*H*[a]	*G*[a]	TCGFE[a]	E_S[b]
**INT-a**	-765.733472	-765.718742	-765.778674	0.151388	-766.112663
**TS-a**	-765.685777	-765.670555	-765.731488	0.146553	-766.062095
**INT-b**	-765.688477	-765.672390	-765.735482	0.145851	-766.066212
**O_2**	-150.312830	-150.309523	-150.332809	-0.016204	-150.370488
**HO_2CuPy**	-596.517148	-596.506550	-596.554771	0.069743	-596.771284
**INT1**	-319.515380	-319.507790	-319.546183	0.058613	-319.709329
**TS1**	-319.469962	-319.460992	-319.502777	0.051775	-319.662595
**INT2**	-209.962268	-209.955363	-209.991022	0.047277	-210.111887
**N_2**	-109.515118	-109.511814	-109.533568	-0.012849	-109.559840
***E*-TS2**	-209.955495	-209.949091	-209.983590	0.045818	-210.109496
***Z*-TS2**	-209.956470	-209.950060	-209.984948	0.045645	-210.109881
***E*-2**	-210.069473	-210.062946	-210.097708	0.051125	-210.222077
***Z*-2**	-210.069383	-210.062979	-210.097777	0.051181	-210.221684
**Cu^+Py_2**	-693.614497	-693.601906	-693.655731	0.141739	-693.981660
**Py**	-248.190580	-248.185368	-248.217980	0.061690	-248.355795
**INT3**	-764.894836	-764.878742	-764.940606	0.135644	-765.298793
**TS3**	-764.889112	-764.872889	-764.934696	0.133422	-765.290183
**INT4**	-655.395055	-655.380968	-655.437847	0.127563	-655.752457
***E*-TS4**	-655.383954	-655.370368	-655.425750	0.125755	-655.744288
***Z*-TS4**	-655.386037	-655.372337	-655.428405	0.125175	-655.744857

[a] Computed at the B3LYP(gas)/SDD-6-31G(d) level.

[b] Computed at the B3LYP (CPCM)/SDD-6-311+G(d,p)// B3LYP(gas)/SDD-6-31G(d) level.

Coordinates of All Stationary Points

INT-a

C	-1.70946600	-0.62748500	-0.02954500
C	-2.76594200	0.22225700	0.31060100
N	-2.39372800	-1.75904500	-0.38873700
H	-2.01441200	-2.64170400	-0.69896300
N	-3.73630800	-1.63922200	-0.28445800
N	-3.95950000	-0.42545500	0.14161000
C	-2.73335600	1.65265500	0.76842800
H	-3.37384800	1.76119300	1.65373000
H	-1.71270700	1.90652600	1.08309600
Cu	0.15134200	-0.39446700	-0.03328600
C	2.72106300	0.33309500	-1.12028000
C	2.79984800	-0.31588100	1.10250400
C	4.09223300	0.56552000	-1.14456600
H	2.10529300	0.49498200	-1.99866200
C	4.17383100	-0.10661400	1.15850600
H	2.24595800	-0.66357200	1.96811900
C	4.83448200	0.34219000	0.01501700
H	4.56145800	0.91527100	-2.05803500
H	4.70819600	-0.29362600	2.08392600
H	5.90662200	0.51487400	0.02740400
N	2.07722900	-0.10174900	-0.01688400
C	-3.20467200	2.64319500	-0.31028400
H	-4.22056700	2.39559800	-0.63501500
H	-3.20447000	3.67319700	0.06784600
H	-2.55240200	2.60150800	-1.19114100

TS-a

C	-1.78703700	-0.77929200	-0.05207100
C	-2.73453900	0.27298500	0.29536500
N	-2.27525700	-1.93588300	-0.37889100
H	-1.56894000	-2.63918700	-0.59063900
N	-4.58698500	-1.15754700	-0.05246400
N	-3.97417700	-0.20591300	0.19648800
C	-2.46685800	1.70667000	0.64859100
H	-3.21126200	2.05272000	1.37812900
H	-1.49526200	1.75378900	1.15970000
Cu	0.08332100	-0.42134600	-0.01135000
C	2.70596600	0.03216400	-1.14766700
C	2.71052500	-0.13528800	1.16259600
C	4.08195000	0.23444300	-1.17803400
H	2.11778100	0.01247900	-2.05932700
C	4.08660500	0.06094400	1.21631900
H	2.12586900	-0.28661600	2.06411400
C	4.78738500	0.24977300	0.02516000
H	4.58332000	0.37635900	-2.12962600
H	4.59183500	0.06430100	2.17641300
H	5.86214700	0.40567000	0.03438600
N	2.02388600	-0.15044800	0.00163500
C	-2.44981300	2.67205000	-0.55201900
H	-3.41796400	2.67116800	-1.06579100
H	-2.23301500	3.70023900	-0.23412100
H	-1.68817300	2.36994200	-1.28088900

INT-b

C	-1.75234300	-0.85510200	-0.20414800
C	-2.74721600	0.12723000	0.27136000
N	-2.18587100	-1.97939800	-0.68506600
H	-1.40277900	-2.57410100	-0.96062600
N	-5.05883900	-0.65986300	0.08803600
N	-4.00296300	-0.23006500	0.19322500
C	-2.43276700	1.51329700	0.78401300
H	-3.15994800	1.80475300	1.55348400
H	-1.45708100	1.45883500	1.28604300
Cu	0.10660500	-0.43832600	-0.08045600
C	2.71151100	0.36741600	-1.06997700
C	2.75443200	-0.43546800	1.10163500
C	4.08379900	0.59557100	-1.05809200
H	2.10882800	0.59073600	-1.94439200
C	4.12835500	-0.23690500	1.19326400
H	2.18530300	-0.84218400	1.93135500
C	4.80761400	0.28847800	0.09405700
H	4.56841800	1.00559300	-1.93796200
H	4.64853600	-0.49163900	2.11068600
H	5.88007600	0.45511900	0.13440800
N	2.05041500	-0.14007000	-0.00988700
C	-2.38227500	2.59599700	-0.30843300
H	-3.35239000	2.69434400	-0.80899400
H	-2.11730600	3.57318300	0.11536300
H	-1.63905000	2.33951400	-1.07237600

O_2

O	0.00000000	0.00000000	0.60710800

O	0.00000000	0.00000000	-0.60710800

HO_2CuPy

C	1.27678400	1.15775000	-0.01231400
C	1.54845400	-1.14479300	0.01477300
C	2.65212300	1.36063600	-0.00728000
H	0.58233800	1.99070800	-0.02716100
C	2.93317900	-1.02162900	0.01983200
H	1.06711700	-2.11642100	0.02320400
C	3.49992000	0.25302200	0.00929100
H	3.04327100	2.37255300	-0.01711400
H	3.54945900	-1.91447200	0.03193500
H	4.57838700	0.38030100	0.01341100
N	0.72322500	-0.07515700	-0.00072600
Cu	-1.16887400	-0.27971800	-0.00914100
O	-2.95660100	-0.48316700	-0.03711200
O	-3.47375100	0.91047000	-0.04618800
H	-4.00575900	0.87692300	0.76649100

INT1

C	1.66355500	-0.30549800	-0.00006100
N	2.80555900	-0.54435900	0.00007700
C	0.26566700	-0.08241600	-0.00007100
C	-0.74323300	-1.22485800	-0.00002100
H	-0.54756800	-1.85215000	0.87910400
H	-0.54764600	-1.85216100	-0.87915600
C	-2.20587600	-0.77105100	0.00004100
H	-2.44590800	-0.17523900	-0.88746200
H	-2.86150200	-1.64689600	0.00007600
H	-2.44582900	-0.17522700	0.88755600
N	-0.14127300	1.16337500	-0.00002200
N	-0.52603400	2.23878500	0.00002400

TS1

C	1.55943200	-0.57067900	-0.08364500
N	2.70869600	-0.74142400	0.08037100
C	0.21857400	-0.36717400	-0.46546500
C	-0.81840900	-1.18697000	0.22602100
H	-0.65052400	-1.34259000	1.30186300
H	-0.61348300	-2.17324100	-0.23631400
C	-2.26482400	-0.78270900	-0.06680400
H	-2.42993800	-0.68250600	-1.14384900
H	-2.96290600	-1.52733300	0.32921700
H	-2.50226700	0.17827000	0.40359200
N	-0.06549600	1.47820100	0.21016200
N	-0.21598900	2.54787900	-0.04984000

INT2

C	-1.49388700	-0.10524200	-0.00306500
N	-2.64841400	0.10883800	0.11820000
C	-0.19081100	-0.54788700	-0.16561700
C	0.89612000	0.44041400	-0.09059300
H	0.69720000	1.31717100	0.54703300
H	0.82147200	0.84254800	-1.12970300
C	2.29975300	-0.13394500	0.12564600
H	2.48859900	-0.95908400	-0.56714900
H	3.06319600	0.63715900	-0.02114800
H	2.40138300	-0.51970000	1.14534200

N_2

N	0.00000000	0.00000000	0.55265300
N	0.00000000	0.00000000	-0.55265300

***E*-TS2**

C	1.49259500	-0.09256700	-0.01481300
N	2.63208000	0.17288200	-0.04602400
C	0.16405300	-0.58798200	-0.03380100
C	-0.86947100	0.36136200	0.04603800
H	-0.67511800	1.44339900	0.01640400
H	-0.46478700	-0.19585900	1.10468700
C	-2.32357300	-0.03019000	-0.06890400
H	-2.44523500	-1.10795100	0.06684900
H	-2.95388300	0.51620000	0.64107200
H	-2.66715700	0.23029200	-1.07795700

***Z*-TS2**

C	1.24533000	0.09475800	-0.01759700
N	2.20015800	-0.58248400	0.02442200
C	0.20661800	1.05212100	-0.12793200
C	-1.11607900	0.58312900	-0.01098800
H	-1.89548100	1.34050000	-0.13870800
H	-0.58237400	1.05024800	1.01658300
C	-1.61445200	-0.84648900	-0.00528500
H	-0.82147100	-1.55025700	0.26075700
H	-2.46229300	-0.97199400	0.67613000
H	-1.96799000	-1.09221900	-1.01490500

***E*-2**

C	1.50211500	0.09189200	-0.00006900
N	2.62510300	-0.21441700	0.00022300
C	0.13044100	0.49228400	-0.00025700
C	-0.88530900	-0.38650300	-0.00033400
H	-0.05638500	1.56433000	0.00021600
H	-0.65529200	-1.45097100	-0.00003400
C	-2.33242700	-0.00621800	0.00020500
H	-2.84226700	-0.42034500	-0.87969100

H	-2.84170800	-0.42038000	0.88039300
H	-2.46899100	1.07955900	0.00028100

***Z*-2**

C	-1.24825900	0.07645800	-0.00028200
N	-2.16216000	-0.64471500	0.00016000
C	-0.13899200	0.97891200	0.00001800
C	1.14351100	0.57528400	-0.00011300
H	-0.38809600	2.03676300	0.00040300
H	1.90663500	1.35223300	0.00044600
C	1.62754800	-0.84039000	0.00000600
H	2.25621000	-1.03022200	-0.87991200
H	2.25560700	-1.03033400	0.88037500
H	0.80191000	-1.55701900	-0.00020600

Cu^+Py_2

C	-2.59374500	-0.58111600	1.00790700
C	-2.59377700	0.58110700	-1.00791100
C	-3.98224900	-0.60033900	1.04087100
H	-2.00707900	-1.03685700	1.79805500
C	-3.98228500	0.60036400	-1.04081200
H	-2.00713900	1.03683300	-1.79808900
C	-4.69176600	0.00002200	0.00004500
H	-4.49102200	-1.07921900	1.87032200
H	-4.49108000	1.07925500	-1.87024200
H	-5.77741900	0.00003300	0.00007200
N	-1.90166200	-0.00001000	-0.00002000
Cu	-0.00026700	-0.00003000	-0.00003800
C	2.59379800	1.00805500	0.58098900
C	2.59387800	-1.00804300	-0.58100400
C	3.98242500	1.04116200	0.60032200
H	2.00693700	1.79827600	1.03651000
C	3.98253600	-1.04109500	-0.60025300
H	2.00711800	-1.79829300	-1.03661200
C	4.69181400	0.00003800	0.00006400
H	4.49123600	1.87063300	1.07901800
H	4.49137500	-1.87054200	-1.07896000
H	5.77748600	0.00009000	0.00011300
N	1.90217200	-0.00002800	-0.00003700

Py

C	-1.14277700	-0.72193400	-0.00021700
C	-1.19895300	0.67332800	-0.00012000
C	0.00024400	1.38569300	0.00009400
C	1.19919000	0.67292200	0.00021600
C	1.14252300	-0.72232100	0.00012500
N	-0.00024900	-1.42075700	-0.00009900
H	0.00040800	2.47254800	0.00016200
H	-2.06016500	-1.30893500	-0.00037300
H	-2.15797400	1.18298700	-0.00023100
H	2.15840900	1.18221300	0.00035900
H	2.05970200	-1.30964500	0.00018600

INT3

C	-2.20077600	1.66158300	-0.32628300
N	-2.17206000	2.79843000	-0.57200700
C	-2.83005500	-0.66507700	-1.17435500
H	-2.36912200	-0.36017800	-2.11910200
H	-3.89086100	-0.39404800	-1.23639500
C	-2.66174800	-2.16863600	-0.95269100
H	-3.11717700	-2.50549100	-0.01440100
H	-3.15775800	-2.71074400	-1.76293300
H	-1.60586700	-2.46297900	-0.94989400
N	-2.66035800	-0.05355200	1.18670000
N	-3.03291400	-0.35158900	2.19697200
C	-2.14822700	0.22849300	-0.09316200
Cu	-0.16148800	0.01303900	0.02646700
C	2.42795700	-1.21928700	0.13908300
C	2.46330300	1.09791600	-0.08760300
C	3.81540000	-1.27912400	0.13504100
H	1.82815500	-2.11820200	0.23151200
C	3.85224100	1.11323200	-0.09898800
H	1.89110400	2.01543700	-0.17386200
C	4.54277000	-0.09406700	0.01394300
H	4.31019000	-2.23994900	0.22567800
H	4.37559900	2.05820100	-0.19543300
H	5.62825200	-0.11146200	0.00774100
N	1.75468100	-0.05027300	0.02971300

TS3

C	-2.40254100	1.58660800	-0.45348300
N	-2.59718900	2.71609300	-0.66035900
C	-2.87545200	-0.78814000	-1.09037900
H	-2.55748800	-0.55367300	-2.11878100
H	-3.94411500	-0.54776500	-1.04639700
C	-2.61865000	-2.26529800	-0.79000500
H	-2.93790500	-2.53519500	0.22327300
H	-3.18724300	-2.88793200	-1.48614200
H	-1.55830800	-2.52021100	-0.89765400
N	-2.62104800	-0.05338600	1.37528400
N	-2.78697500	-0.23411300	2.45497000
C	-2.05555400	0.19854200	-0.27018100
Cu	-0.14033300	0.03276200	-0.07926700

C	2.45819900	-1.18606800	0.04583300
C	2.48500500	1.13991300	-0.03411900
C	3.84542300	-1.23926800	0.08469500
H	1.86176900	-2.09198100	0.06072000
C	3.87335800	1.16329100	0.00183300
H	1.90950800	2.05822600	-0.08270000
C	4.56814200	-0.04542200	0.06230100
H	4.34373500	-2.20141400	0.13118600
H	4.39331400	2.11482100	-0.01817100
H	5.65334800	-0.05707000	0.09116300
N	1.78027900	-0.01614400	-0.01228400

INT4

C	-2.85469600	-1.47185100	-0.03179600
N	-3.20591500	-2.58850000	-0.09450400
C	-3.32211800	0.93146900	0.13382400
H	-3.64221500	0.83904900	1.19934600
H	-4.24441500	0.68941600	-0.41719500
C	-2.81929500	2.34880900	-0.14045000
H	-2.52515700	2.46039800	-1.18897100
H	-3.61170900	3.07362400	0.06444100
H	-1.96196800	2.59750200	0.49465500
C	-2.33819500	-0.16456900	0.03018400
Cu	-0.47513100	-0.05298500	0.02410300
C	2.15509600	-1.16903600	0.00195100
C	2.13151700	1.16067300	0.00355100
C	3.54323100	-1.19287000	-0.00981200
H	1.57802900	-2.08772900	0.00606600
C	3.51871200	1.21273200	-0.00827300
H	1.53600200	2.06739800	0.00862900
C	4.23937900	0.01703400	-0.01497600
H	4.06270100	-2.14481100	-0.01492800
H	4.01922800	2.17479100	-0.01221900
H	5.32495000	0.02811000	-0.02419500
N	1.45185300	-0.01086000	0.00881100

***E*-TS4**

C	-2.79112200	1.53780200	-0.02608200
N	-3.02733400	2.67427200	-0.11319500
C	-3.37630800	-0.81573300	-0.04925400
H	-4.41446000	-0.48372300	-0.14157900
H	-2.83544200	-0.34986800	1.11292900
C	-3.13691400	-2.28698000	-0.09471100
H	-2.10130200	-2.55005300	0.13575900
H	-3.82639300	-2.82951400	0.56006400
H	-3.35568300	-2.61136400	-1.12274600
C	-2.36540900	0.16309400	0.03602200
Cu	-0.45439000	-0.01432100	0.05885400
C	2.16972900	-1.19446500	-0.00016100
C	2.15041100	1.13199400	0.00560900
C	3.55780500	-1.22181400	-0.03531300
H	1.59169100	-2.11219400	0.01196900
C	3.53813800	1.18188200	-0.02986900
H	1.55715400	2.04012400	0.02237500
C	4.25690800	-0.01392800	-0.05064200
H	4.07505100	-2.17492000	-0.05060400
H	4.03893400	2.14376700	-0.04100800
H	5.34216600	-0.00498400	-0.07845400
N	1.46821600	-0.03756600	0.02047100

***Z*-TS4**

C	2.74228400	1.26662000	-0.02817000
N	3.06003100	2.38161500	-0.13831400
C	3.14968900	-1.13209700	0.04456200
H	2.72574400	-2.13523000	0.10372900
H	2.64954700	-0.63221300	1.17150600
C	4.62672200	-1.03424500	-0.16391600
H	4.99823400	-0.00951200	-0.09424600
H	5.16808200	-1.68329100	0.53153300
H	4.83431900	-1.41125100	-1.17599800
C	2.21922400	-0.07048500	0.06254900
Cu	0.30448700	-0.13272700	0.07563400
C	-2.35201200	-1.21715000	-0.05714800
C	-2.26174300	1.10553200	0.04967500
C	-3.73977400	-1.19972800	-0.10892700
H	-1.80203700	-2.15182100	-0.07871400
C	-3.64669300	1.19987200	0.00103300
H	-1.64119200	1.99317100	0.11219400
C	-4.40129100	0.02887200	-0.07968600
H	-4.28561900	-2.13476200	-0.17156900
H	-4.11777800	2.17639400	0.02611200
H	-5.48533400	0.07281300	-0.11932200
N	-1.61610400	-	

References

(1) P. J. Black, M. G. Edwards and J. M. J. Williams, *Eur. J. Org. Chem.,* 2006, 4367.

(2) (a) C. Hayashi, T. Hayashi, S. Kikuchi and T. Yamada, *Chem. Lett.,* 2014, **43**, 565; (b) Z. Zhang and L. S. Liebeskind, *Org. Lett.,* 2006, **8**, 4331.

(3) S. T. Kemme, T. Šmejkal and B. Breit, *Chem. Eur. J.,* 2010, **16**, 3423.

(4) T. Kochi, S. Noda, K. Yoshimura and K. Nozaki, *J. Am. Chem. Soc.,* 2007, **129**, 8948.

(5) C. M. McMahon and E. J. Alexanian, *Angew. Chem., Int. Ed.,* 2014, **53**, 5974.

(6) M. Tobisu, H. Kinuta, Y. Kita, E. Rémond and N. Chatani, *J. Am. Chem. Soc.,* 2011, **134**, 115.

(7) H. Luo and S. Ma, *Eur. J. Org. Chem.*, 2013, 3041.

(8) P. Wipf, Y. Aoyama and T. E. Benedum, *Org. Lett.*, 2004, **6**, 3593.

(9) A. Jaganathan, A. Garzan, D. C. Whitehead, R. J. Staples and B. Borhan, *Angew. Chem., Int. Ed.*, 2011, **50**, 2593.

(10) K. Jouvin, R. Veillard, C. Theunissen, C. Alayrac, A.-C. Gaumont and G. Evano, *Org. Lett.*, 2013, **15**, 4592.

(11) M. Fukushima, D. Takushima, H. Satomura, G. Onodera and M. Kimura, *Chem.-Eur. J.*, 2012, **18**, 8019.

(12) (a) K. Fujita, H. Yorimitsu, H. Shinokubo and K. Oshima, *J. Am. Chem. Soc.*, 2004, **126**, 6776; (b) S. E. Tully and B. F. Cravatt, *J. Am. Chem. Soc.*, 2010, **132**, 3264.

(13) M. J. Frisch, G. W. Trucks, H. B. Schlegel, G. E. Scuseria, M. A. Robb, J. R. Cheeseman, G. Scalmani, V. Barone, B. Mennucci, G. A. Petersson, H. Nakatsuji, M. Caricato, X. Li, H. P. Hratchian, A. F. Izmaylov, J. Bloino, G. Zheng, J. L. Sonnenberg, M. Hada, M. Ehara, K. Toyota, R. Fukuda, J. Hasegawa, M. Ishida, T. Nakajima, Y. Honda, O. Kitao, H. Nakai, T. Vreven, J. A. Montgomery, Jr., J. E. Peralta, F. Ogliaro, M. Bearpark, J. J. Heyd, E. Brothers, K. N. Kudin, V. N. Staroverov, R. Kobayashi, J. Normand, K. Raghavachari, A. Rendell, J. C. Burant, S. S. Iyengar, J. Tomasi, M. Cossi, N. Rega, J. M. Millam, M. Klene, J. E. Knox, J. B. Cross, V. Bakken, C. Adamo, J. Jaramillo, R. Gomperts, R. E. Stratmann, O. Yazyev, A. J. Austin, R. Cammi, C. Pomelli, J. W. Ochterski, R. L. Martin, K. Morokuma, V. G. Zakrzewski, G. A. Voth, P. Salvador, J. J. Dannenberg, S. Dapprich, A. D. Daniels, Ö. Farkas, J. B. Foresman, J. V. Ortiz, J. Cioslowski and D. J. Fox, *Gaussian 09*, Revision A.01,

Gaussian, Inc., Wallingford CT, 2009.

(14) (a) A. D. Becke, *J. Chem. Phys.,* 1993, **98**, 5648; (b) C. Lee, W. Yang and R. G. Parr, *Phys. Rev. B,* 1988, **37**, 785.

(15) W. J. Hehre, L. Radom, P. v. R. Schleyer and J. A. Pople, *Ab Initio Molecular Orbital Theory*; Wiley: New York, 1986.

(16) (a) K. Fukui, *J. Phys. Chem.,* 1970, **74**, 4161; (b) C. Gonzalez and H. B. Schlegel, *J. Chem. Phys.,* 1989, **90**, 2154; (c) C. Gonzalez and H. B. Schlegel, *J. Phys. Chem.,* 1990, **94**, 5523.

(17) (a) V. Barone and M. Cossi, *J. Phys. Chem. A,* 1998, **102**, 1995; (b) M. Cossi, N. Rega, G. Scalmani and V. Barone, *J. Comput. Chem.,* 2003, **24**, 669.

^{1}H NMR and ^{13}C NMR Spectra of Products

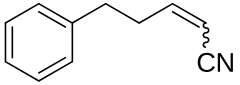

2a

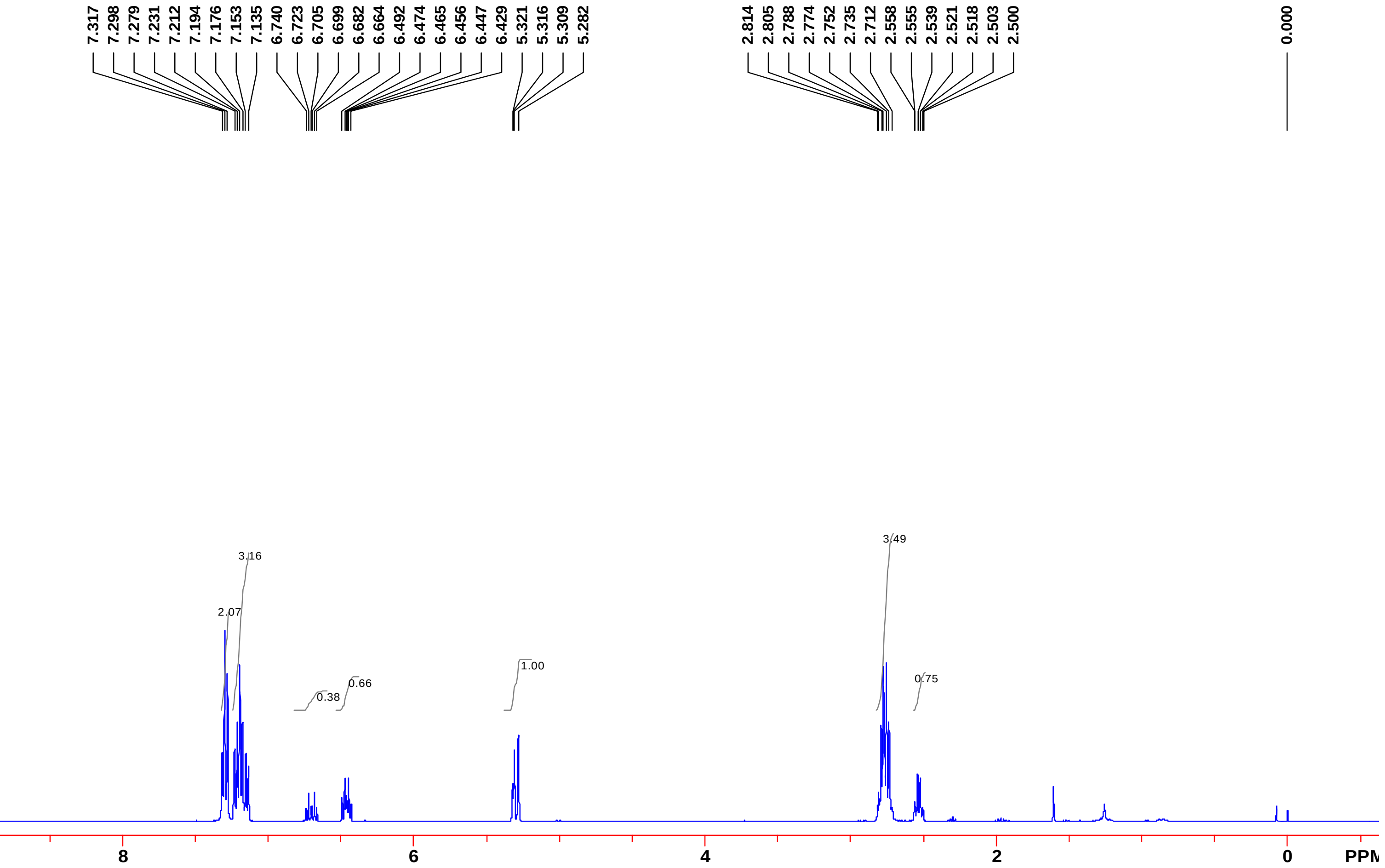

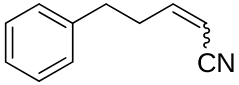
CN

2a

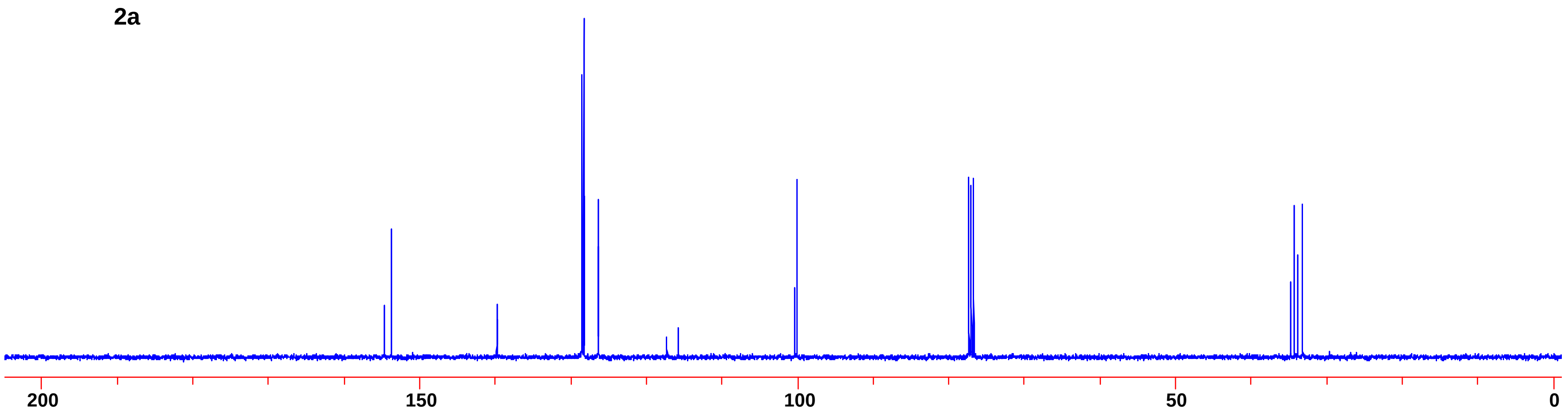
154.618
153.728
139.792
139.740
128.554
128.510
128.310
128.226
126.397
126.353
117.283
115.744
100.371
100.115
77.319
77.000
76.679
34.759
34.230
33.816
33.158
200
150
100
50
0

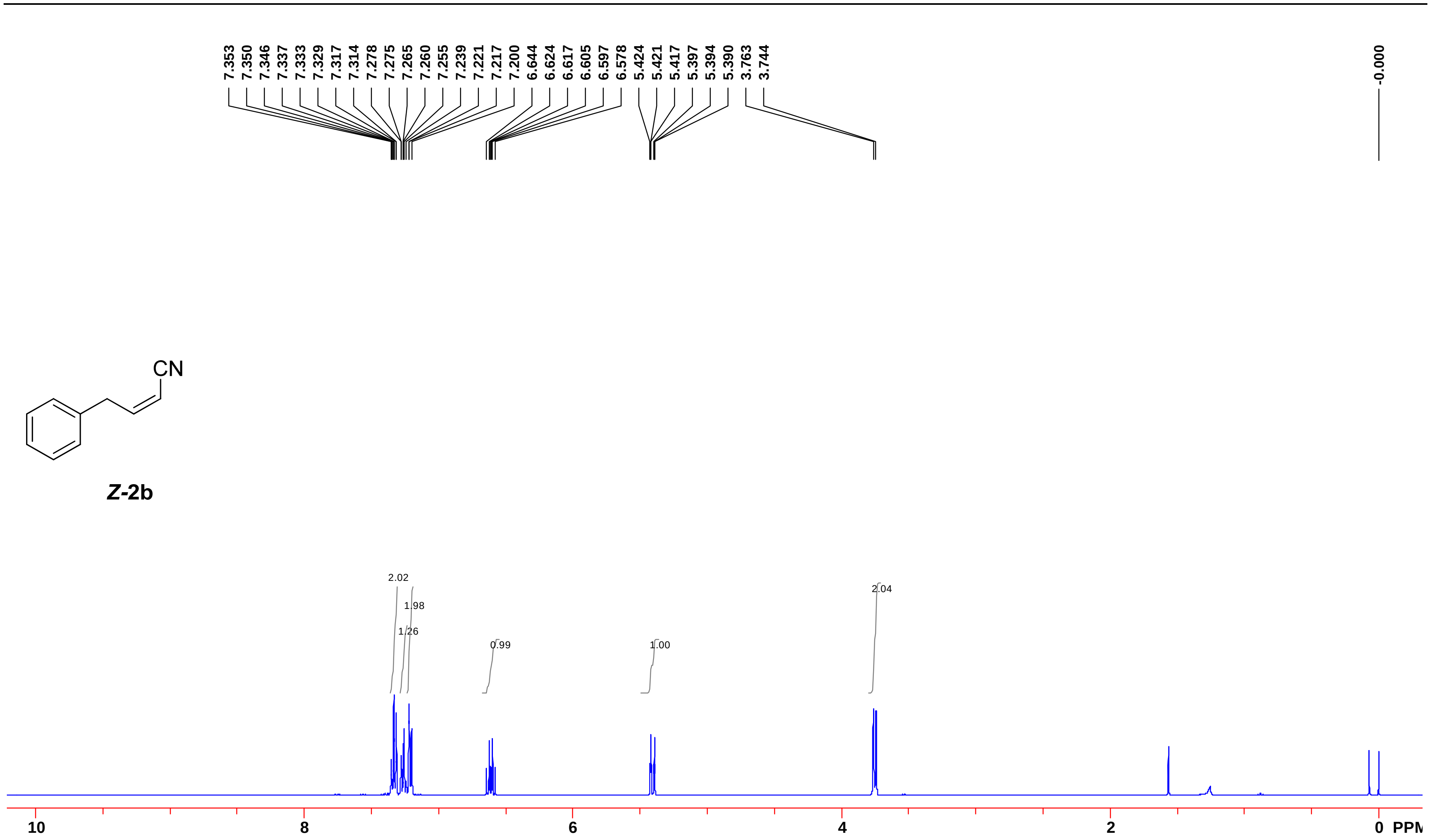
7.353
7.350
7.346
7.337
7.333
7.329
7.317
7.314
7.278
7.275
7.265
7.260
7.255
7.239
7.221
7.217
7.200
6.644
6.624
6.617
6.605
6.597
6.578
5.424
5.421
5.417
5.397
5.394
5.390
3.763
3.744
-0.000
CN
Z-2b
2.02
1.98
1.26
0.99
1.00
2.04
10
8
6
4
2
0 PPM

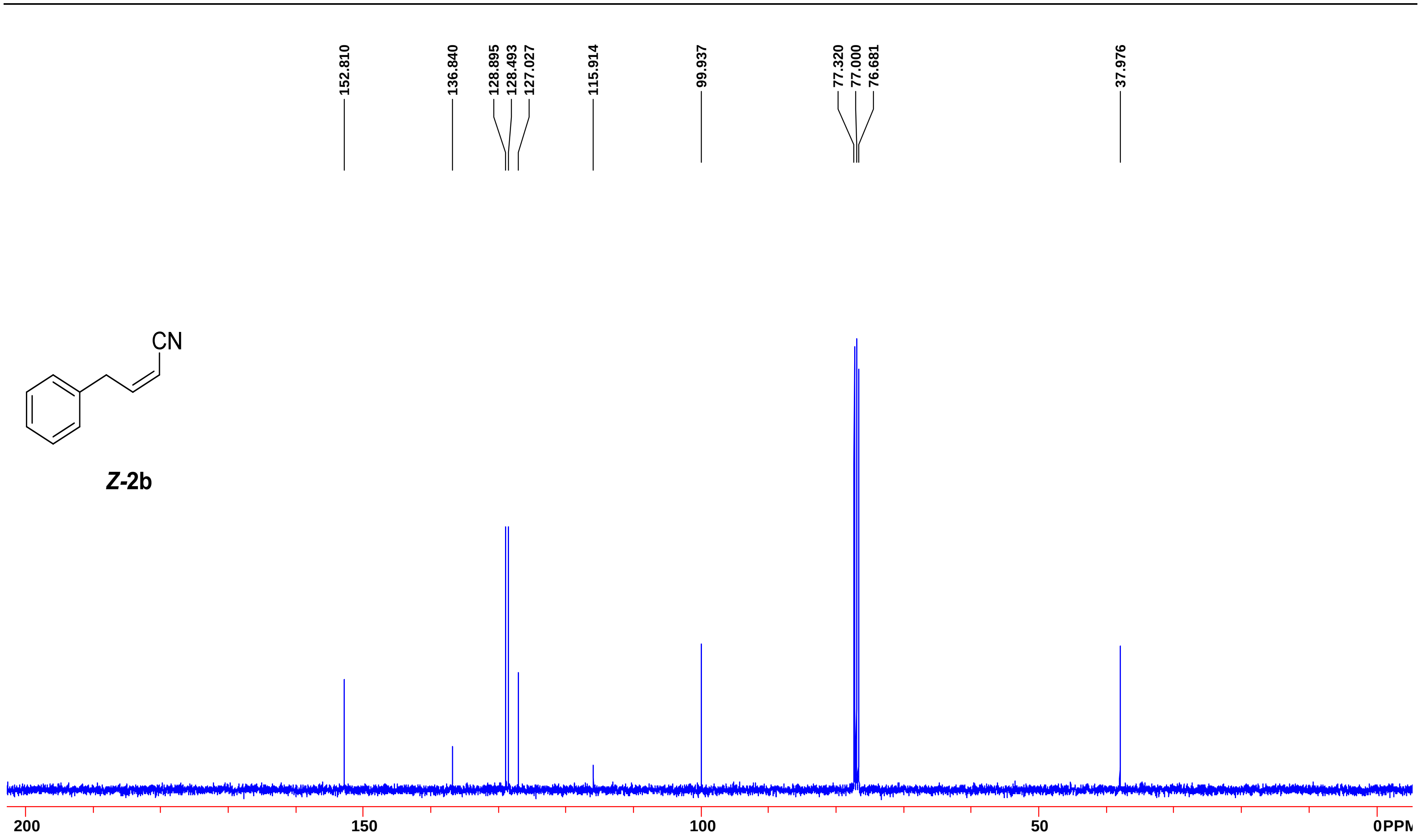
152.810
136.840
128.895
128.493
127.027
115.914
99.937
77.320
77.000
76.681
37.976
200
150
100
50
0PPM
CN

***Z*-2b**

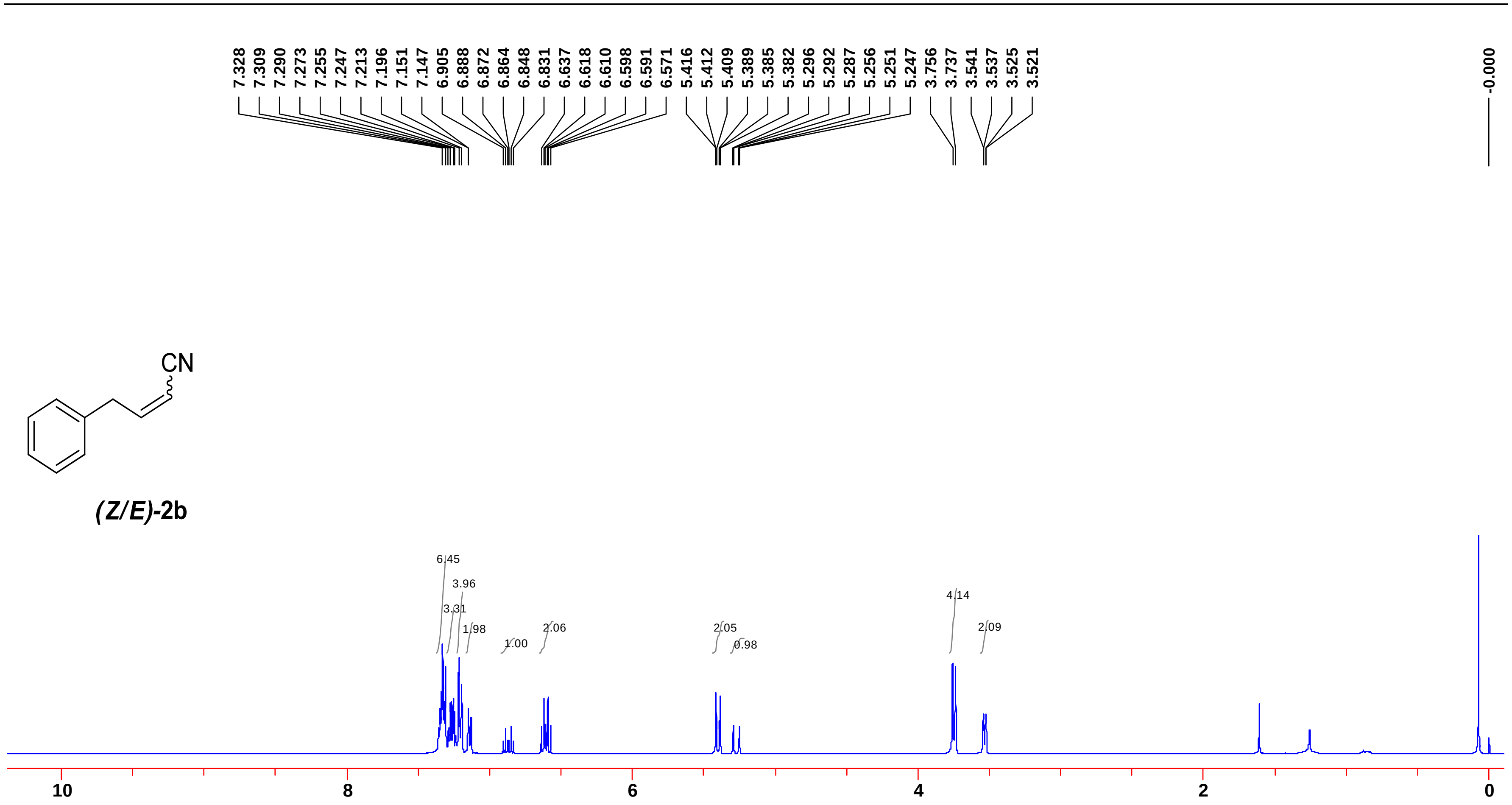

CN
(Z/E)-2b
7.328 7.309 7.290 7.273 7.255 7.247 7.213 7.196 7.151 7.147 6.905 6.888 6.872 6.864 6.848 6.831 6.637 6.618 6.610 6.598 6.591 6.571 5.416 5.412 5.409 5.389 5.385 5.382 5.296 5.292 5.287 5.256 5.251 5.247 3.756 3.737 3.541 3.537 3.525 3.521
-0.000
6.45
3.96
3.31
1.98
1.00
2.06
2.05
0.98
4.14
2.09
10
8
6
4
2
0

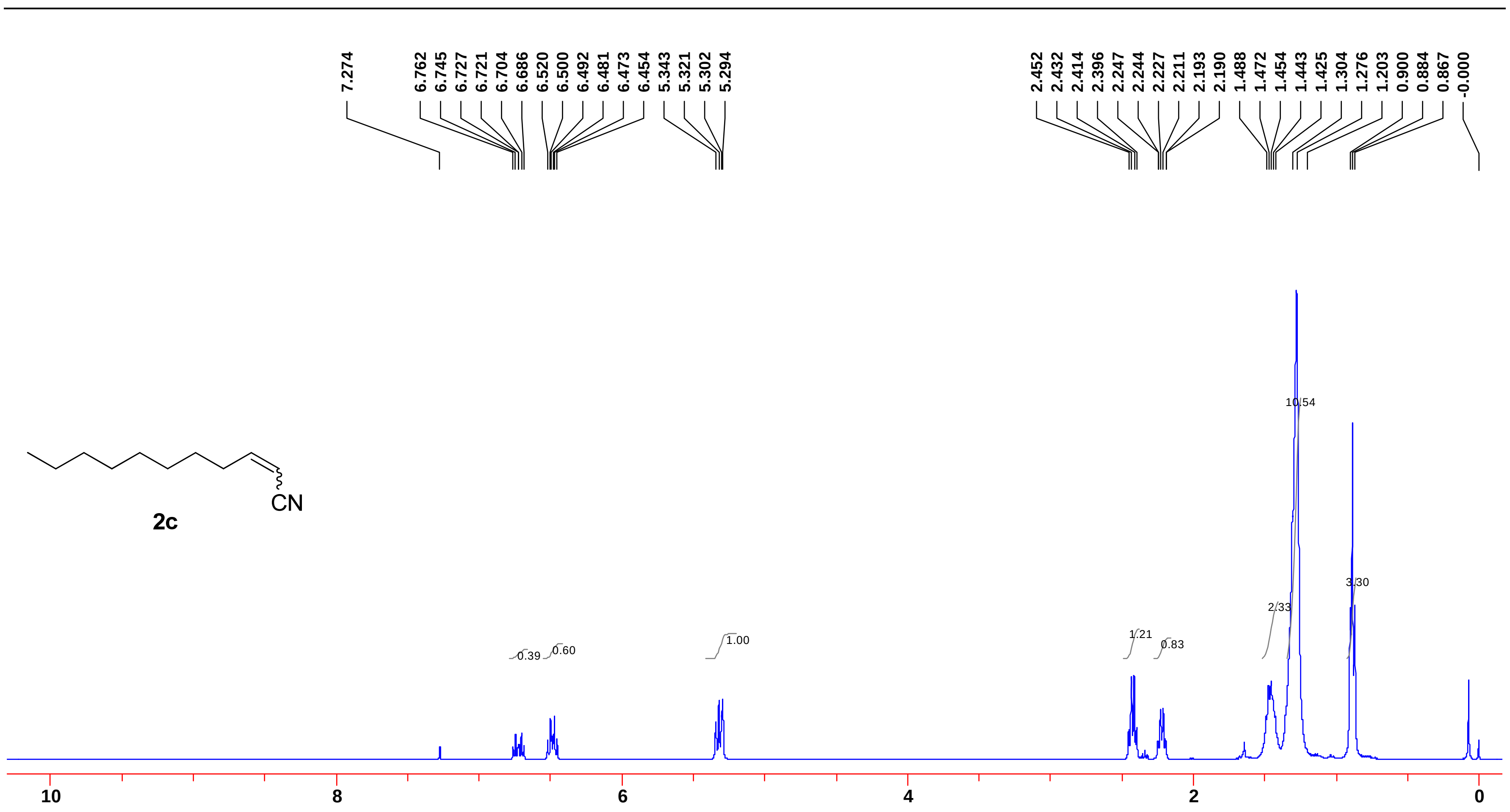
7.274
6.762
6.745
6.727
6.721
6.704
6.686
6.520
6.500
6.492
6.481
6.473
6.454
5.343
5.321
5.302
5.294
2.452
2.432
2.414
2.396
2.247
2.244
2.227
2.211
2.193
2.190
1.488
1.472
1.454
1.443
1.425
1.304
1.276
1.203
0.900
0.884
0.867
-0.000
CN
2c
0.39
0.60
1.00
1.21
0.83
2.33
10.54
3.30
10
8
6
4
2
0

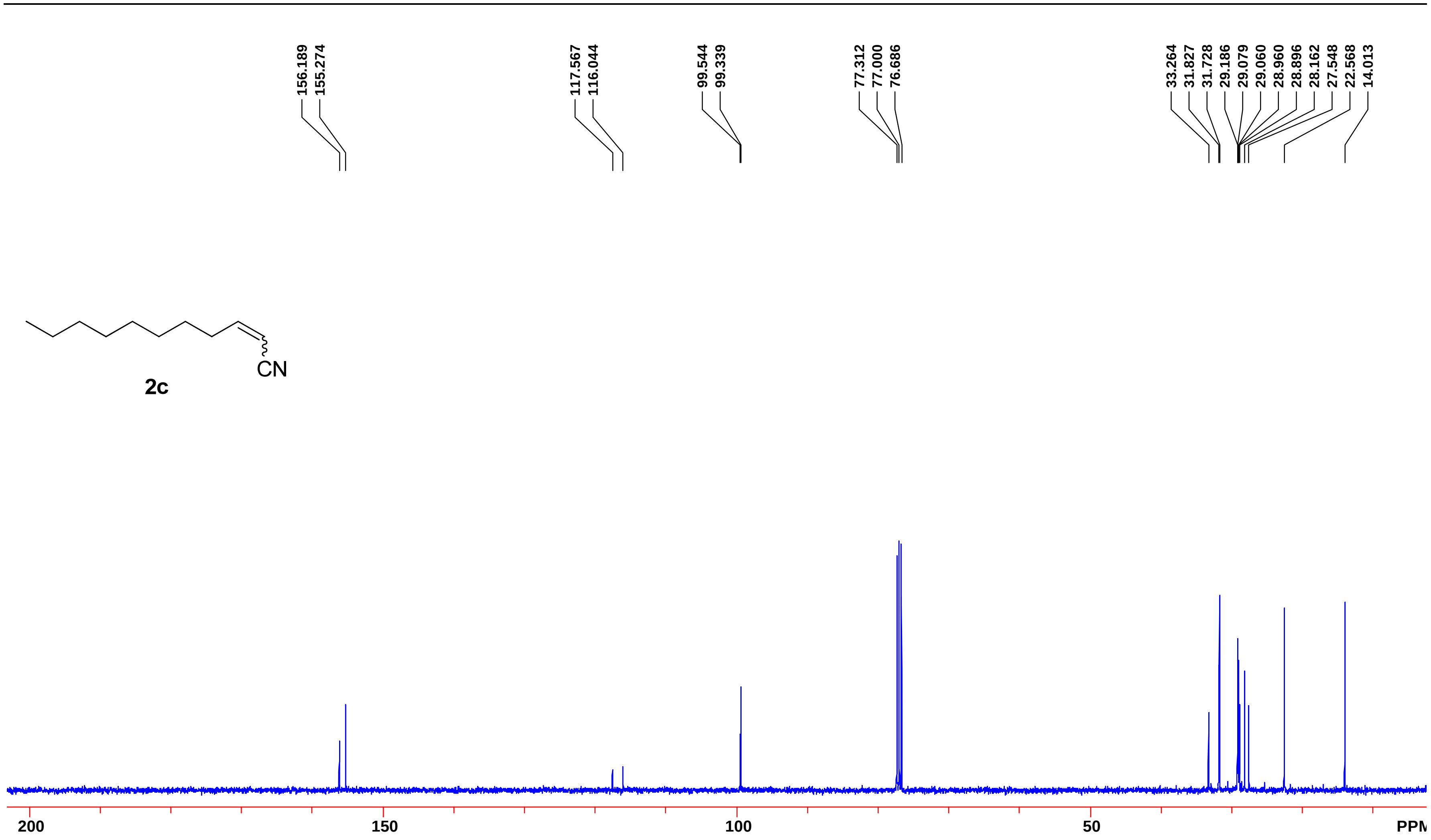
156.189
155.274
117.567
116.044
99.544
99.339
77.312
77.000
76.686
33.264
31.827
31.728
29.186
29.079
29.060
28.960
28.896
28.162
27.548
22.568
14.013
200
150
100
50
PPM
CN

2c

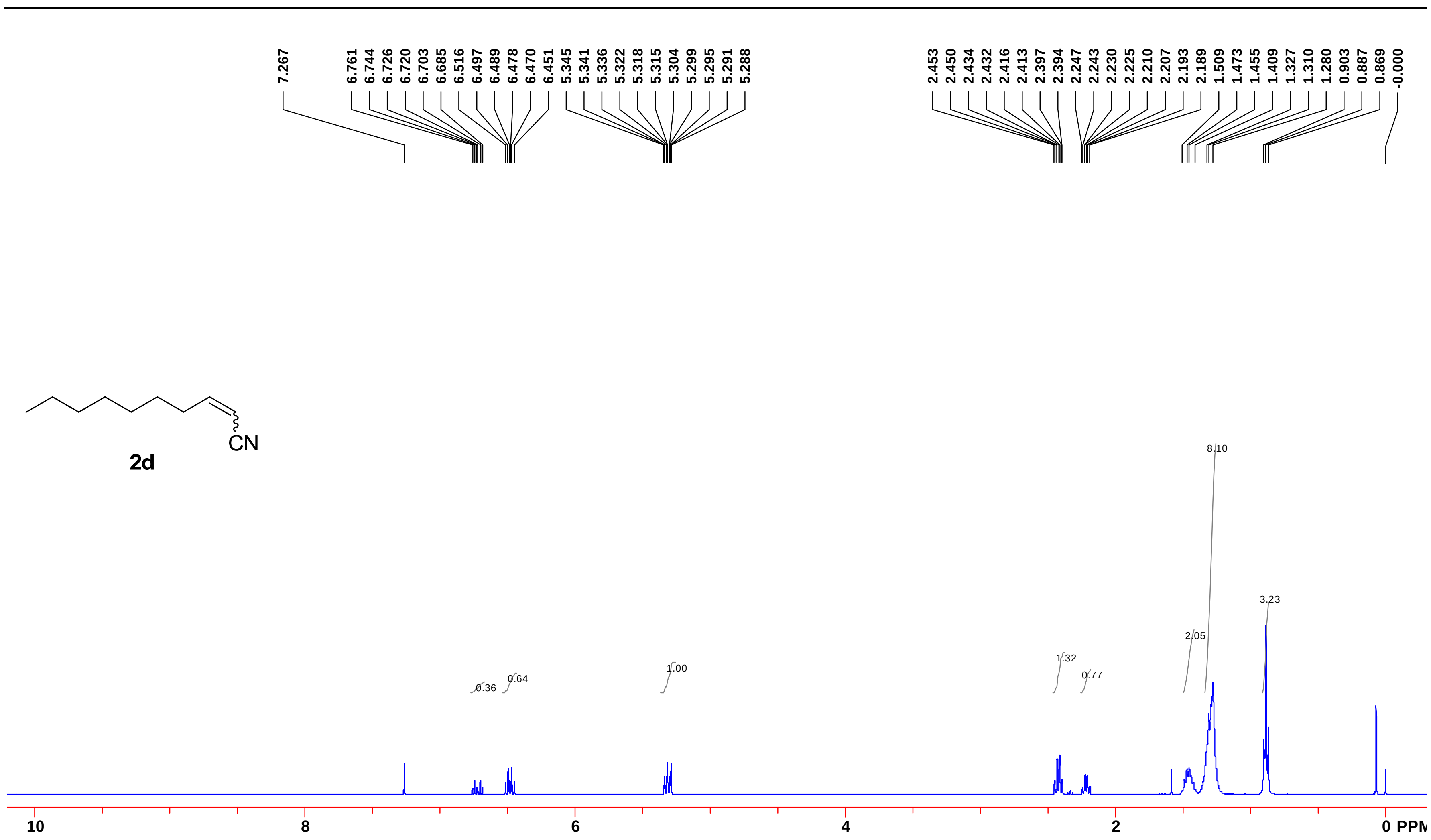
7.267
6.761
6.744
6.726
6.720
6.703
6.685
6.516
6.497
6.489
6.478
6.470
6.451
5.345
5.341
5.336
5.322
5.318
5.315
5.304
5.299
5.295
5.291
5.288
2.453
2.450
2.434
2.432
2.416
2.413
2.397
2.394
2.247
2.243
2.230
2.225
2.210
2.207
2.193
2.189
1.509
1.473
1.455
1.409
1.327
1.310
1.280
0.903
0.887
0.869
-0.000
CN
2d
0.36
0.64
1.00
1.32
0.77
2.05
8.10
3.23
10
8
6
4
2
0 PPM

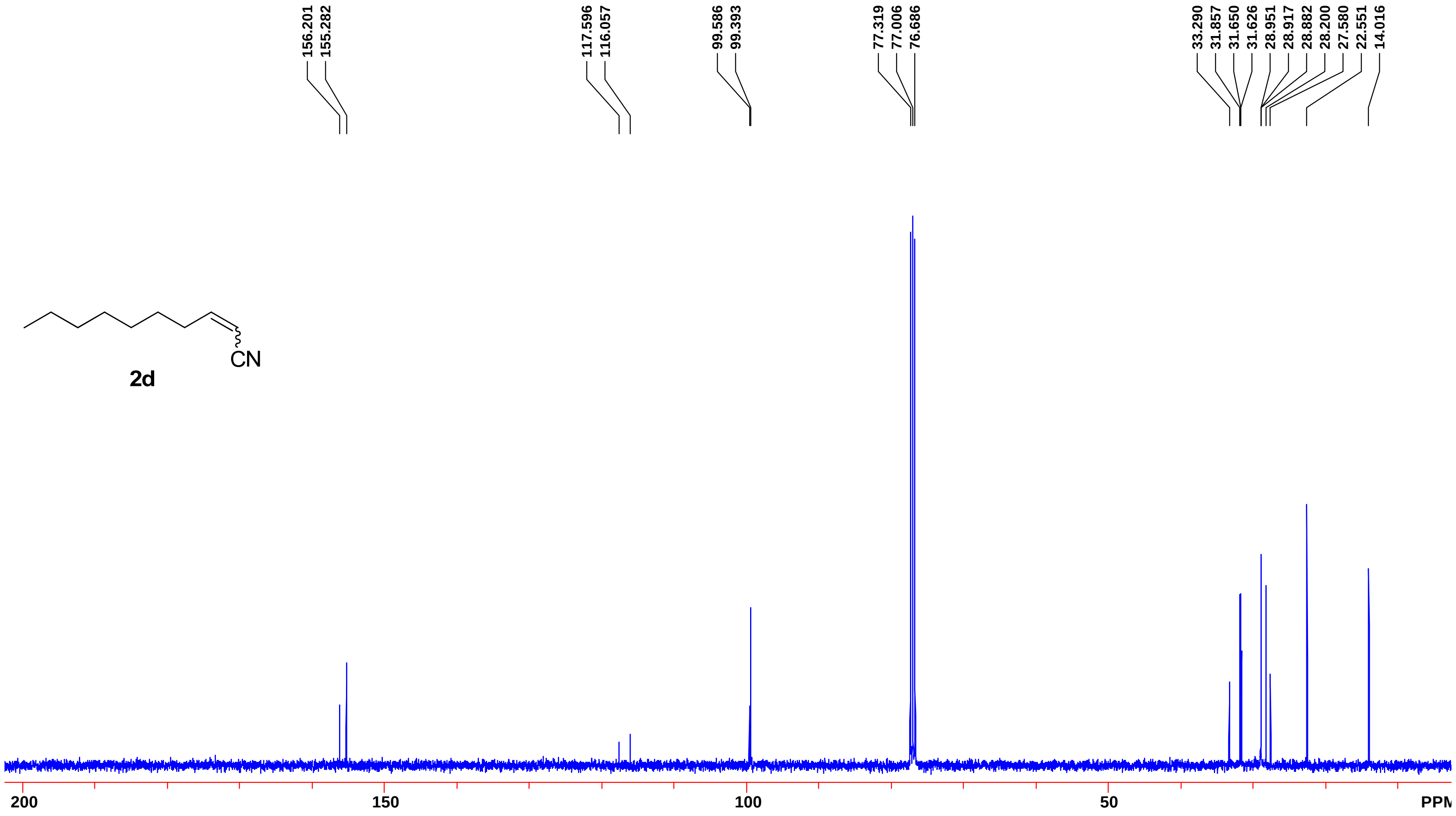
156.201
155.282
117.596
116.057
99.586
99.393
77.319
77.006
76.686
33.290
31.857
31.650
31.626
28.951
28.917
28.882
28.200
27.580
22.551
14.016
CN
2d
200
150
100
50
PPM

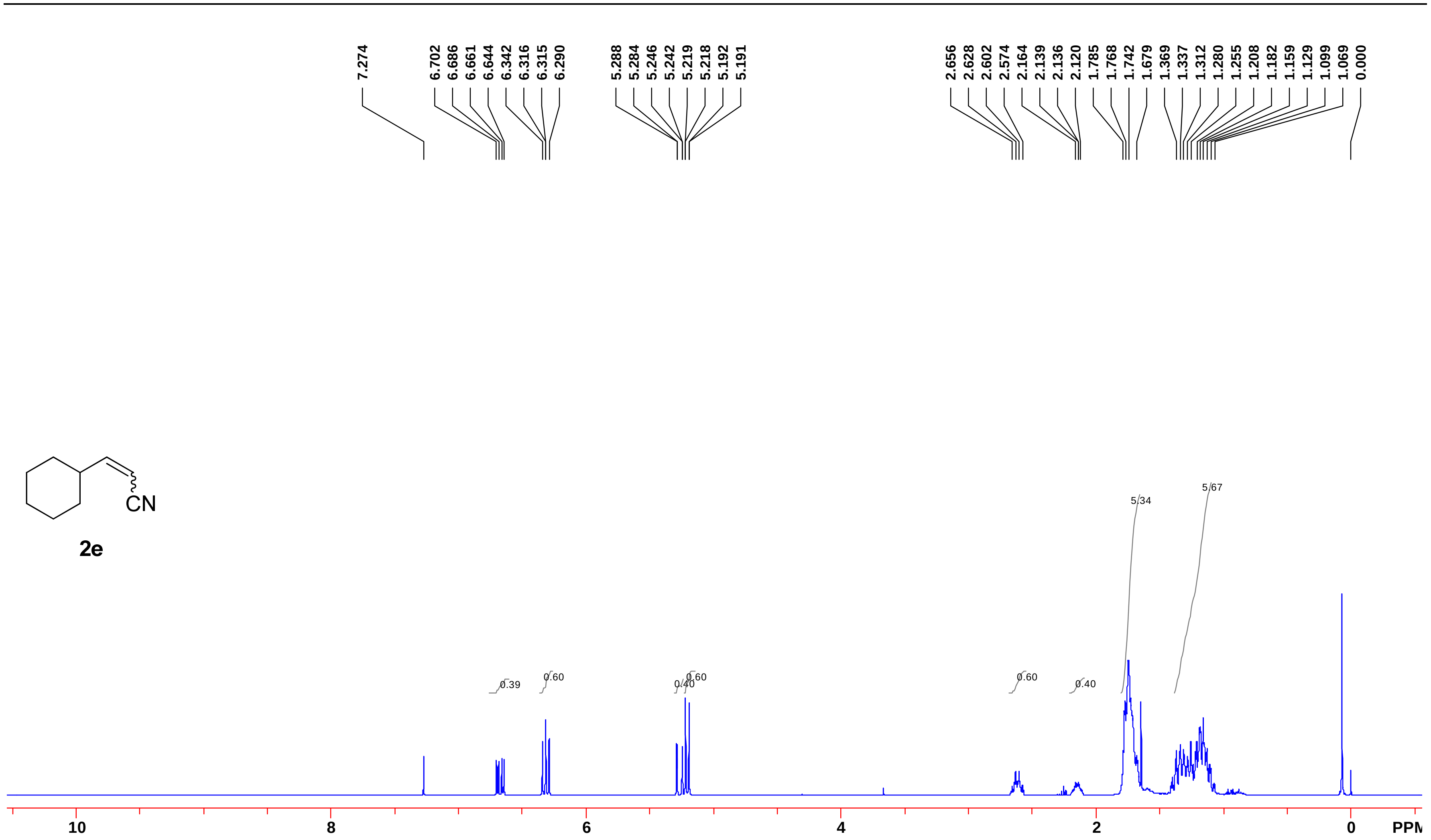
7.274
6.702
6.686
6.661
6.644
6.342
6.316
6.315
6.290
5.288
5.284
5.246
5.242
5.219
5.218
5.192
5.191
2.656
2.628
2.602
2.574
2.164
2.139
2.136
2.120
1.785
1.768
1.742
1.679
1.369
1.337
1.312
1.280
1.255
1.208
1.182
1.159
1.129
1.099
1.069
0.000
CN
2e
0.39
0.60
0.40
0.60
0.60
0.40
5.34
5.67
10
8
6
4
2
0
PPM

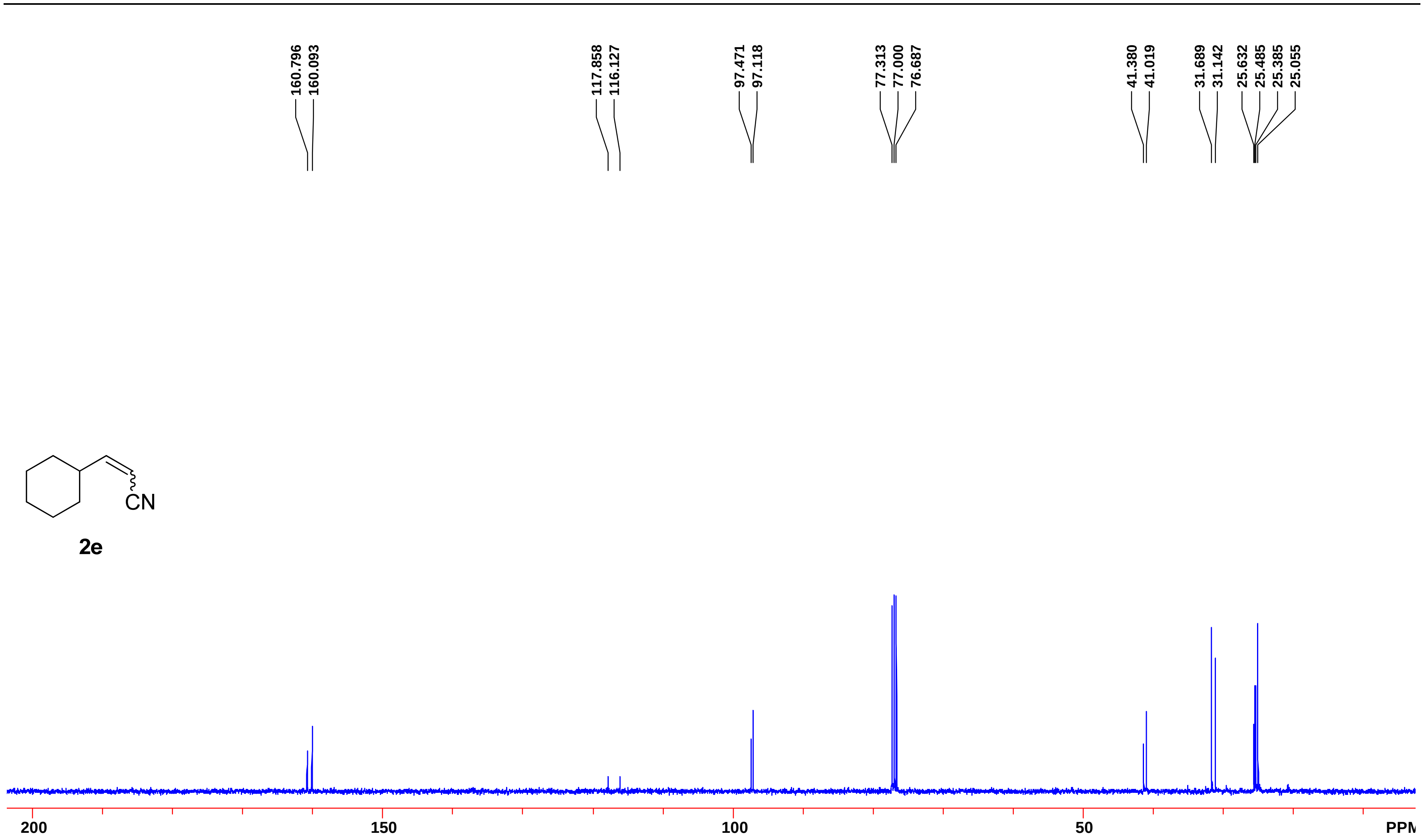
160.796
160.093
117.858
116.127
97.471
97.118
77.313
77.000
76.687
41.380
41.019
31.689
31.142
25.632
25.485
25.385
25.055
200
150
100
50
PPM
CN

2e

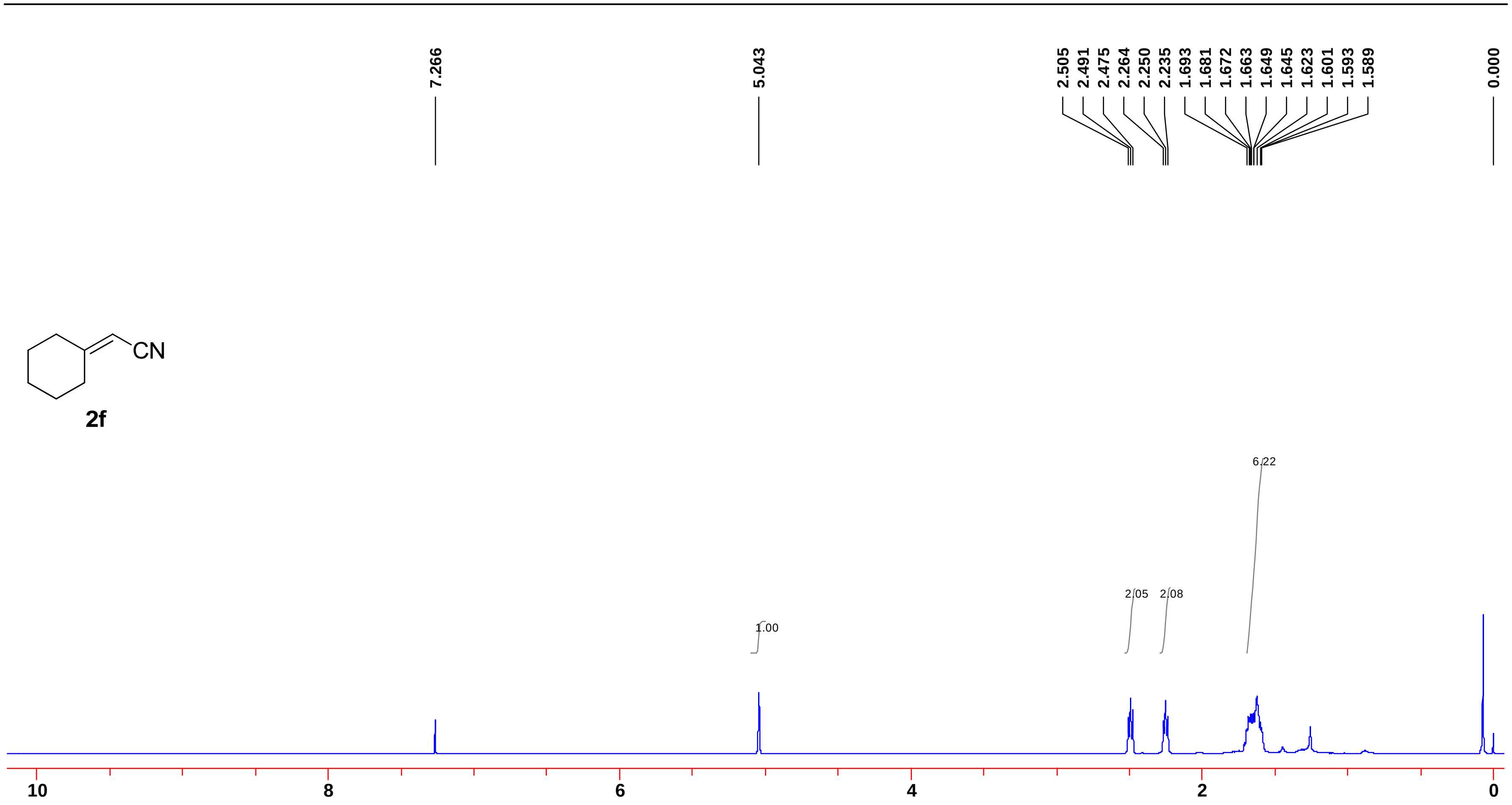
CN
7.266
5.043
2.505
2.491
2.475
2.264
2.250
2.235
1.693
1.681
1.672
1.663
1.649
1.645
1.623
1.601
1.593
1.589
0.000
6.22
2.05
2.08
1.00
10
8
6
4
2
0

2f

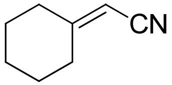
CN

2f

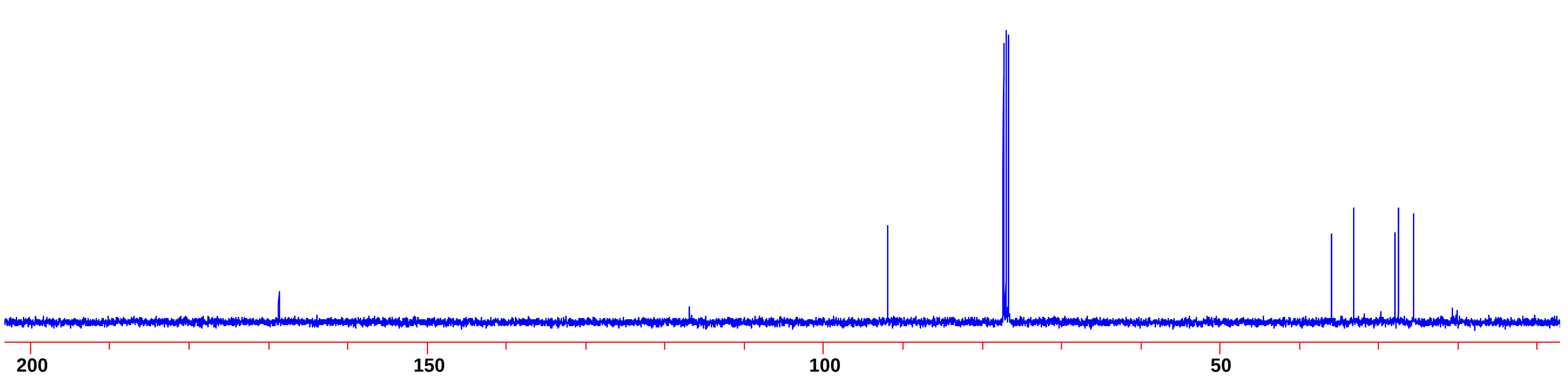
168.671
116.980
91.948
77.313
77.000
76.684
35.947
33.131
27.898
27.509
25.556
200
150
100
50

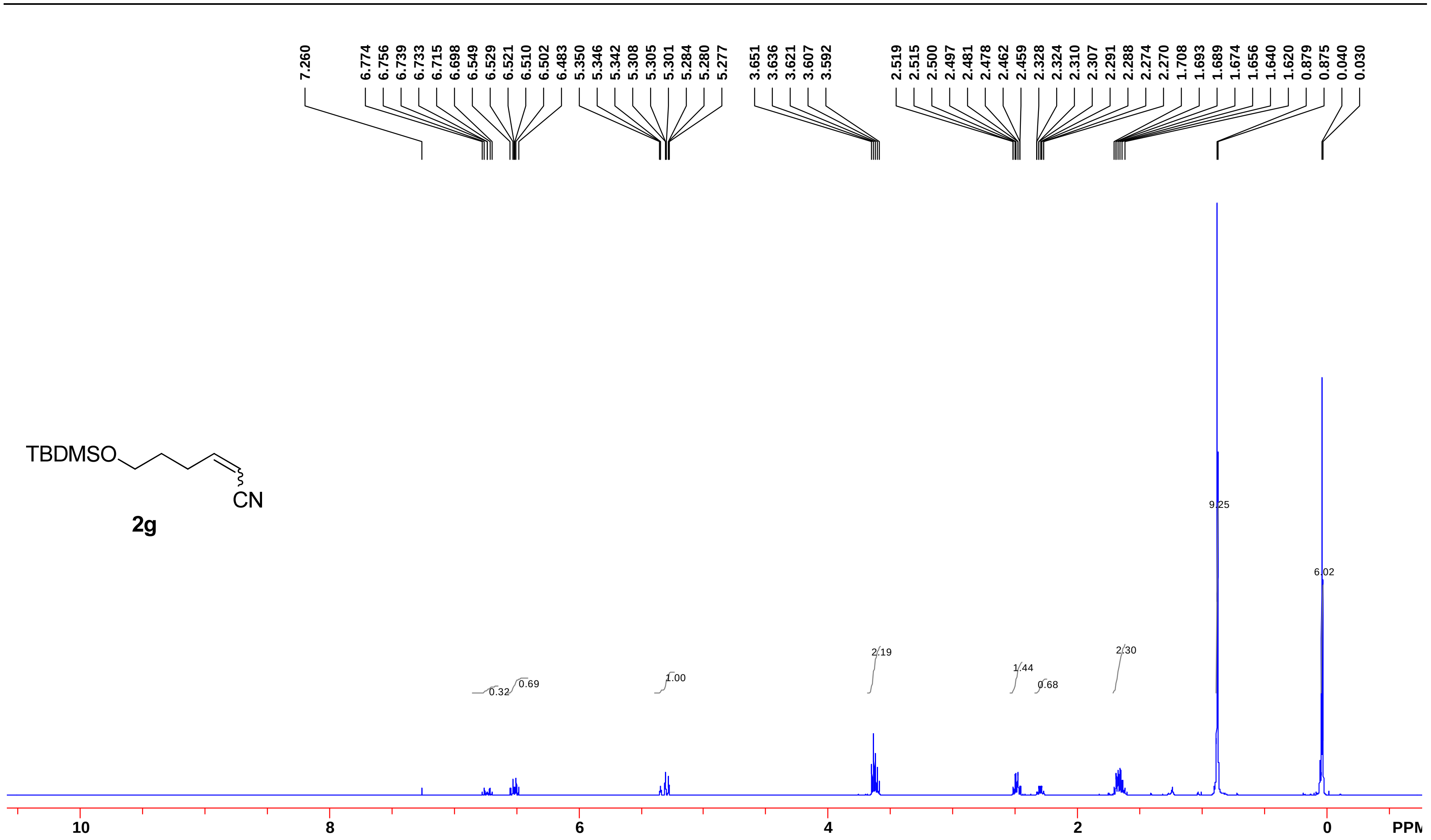
TBDMSO
CN
7.260
6.774
6.756
6.739
6.733
6.715
6.698
6.549
6.529
6.521
6.510
6.502
6.483
5.350
5.346
5.342
5.308
5.305
5.301
5.284
5.280
5.277
3.651
3.636
3.621
3.607
3.592
2.519
2.515
2.500
2.497
2.481
2.478
2.462
2.459
2.328
2.324
2.310
2.307
2.291
2.288
2.274
2.270
1.708
1.693
1.689
1.674
1.656
1.640
1.620
0.879
0.875
0.040
0.030
0.32
0.69
1.00
2.19
1.44
0.68
2.30
9.25
6.02
10
8
6
4
2
0
PPM

2g

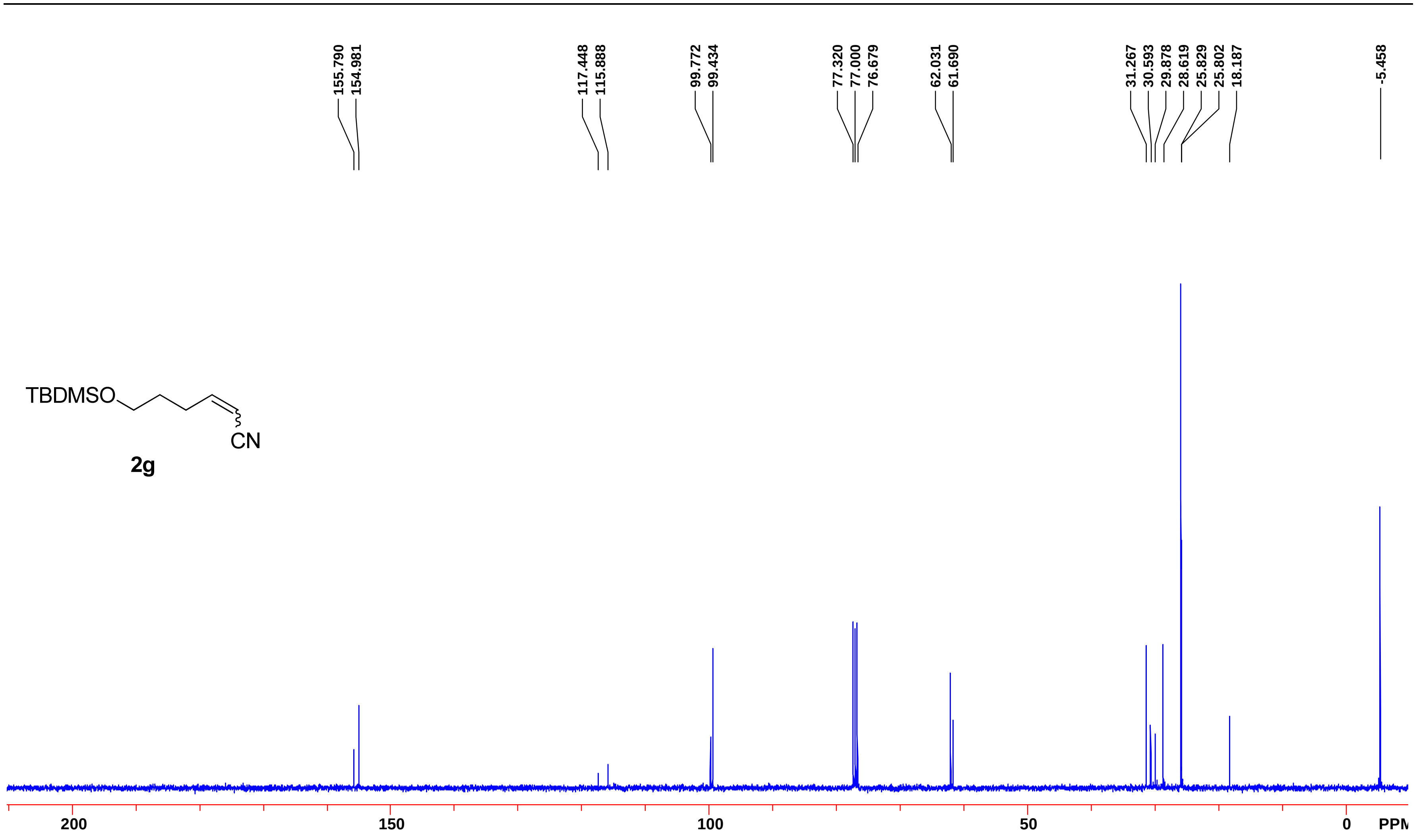
155.790
154.981
117.448
115.888
99.772
99.434
77.320
77.000
76.679
62.031
61.690
31.267
30.593
29.878
28.619
25.829
25.802
18.187
-5.458
TBDMSO
CN
2g
200
150
100
50
0
PPM

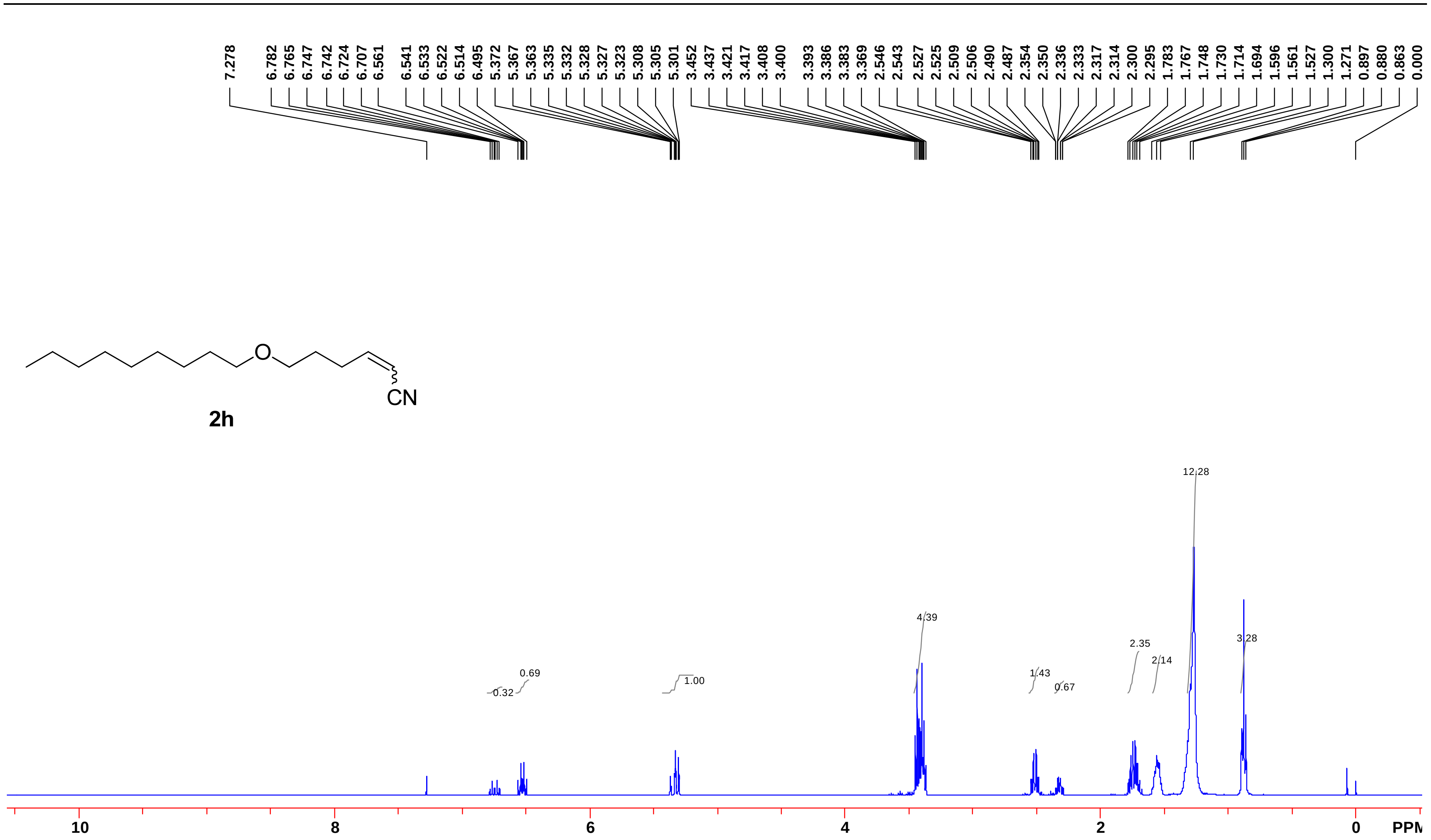
CN
2h
PPM

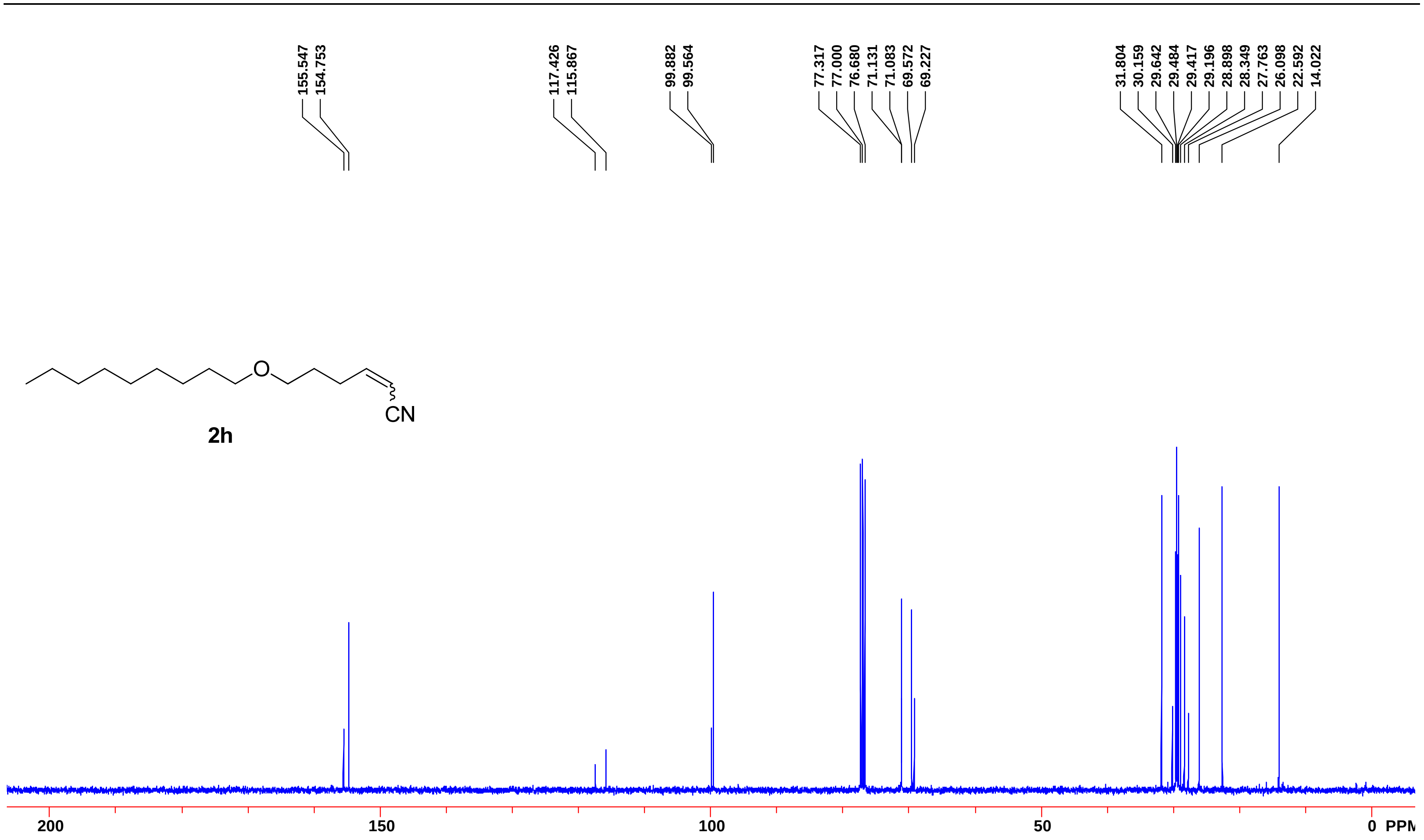
2h
CN
O
155.547
154.753
117.426
115.867
99.882
99.564
77.317
77.000
76.680
71.131
71.083
69.572
69.227
31.804
30.159
29.642
29.484
29.417
29.196
28.898
28.349
27.763
26.098
22.592
14.022
200
150
100
50
0 PPM

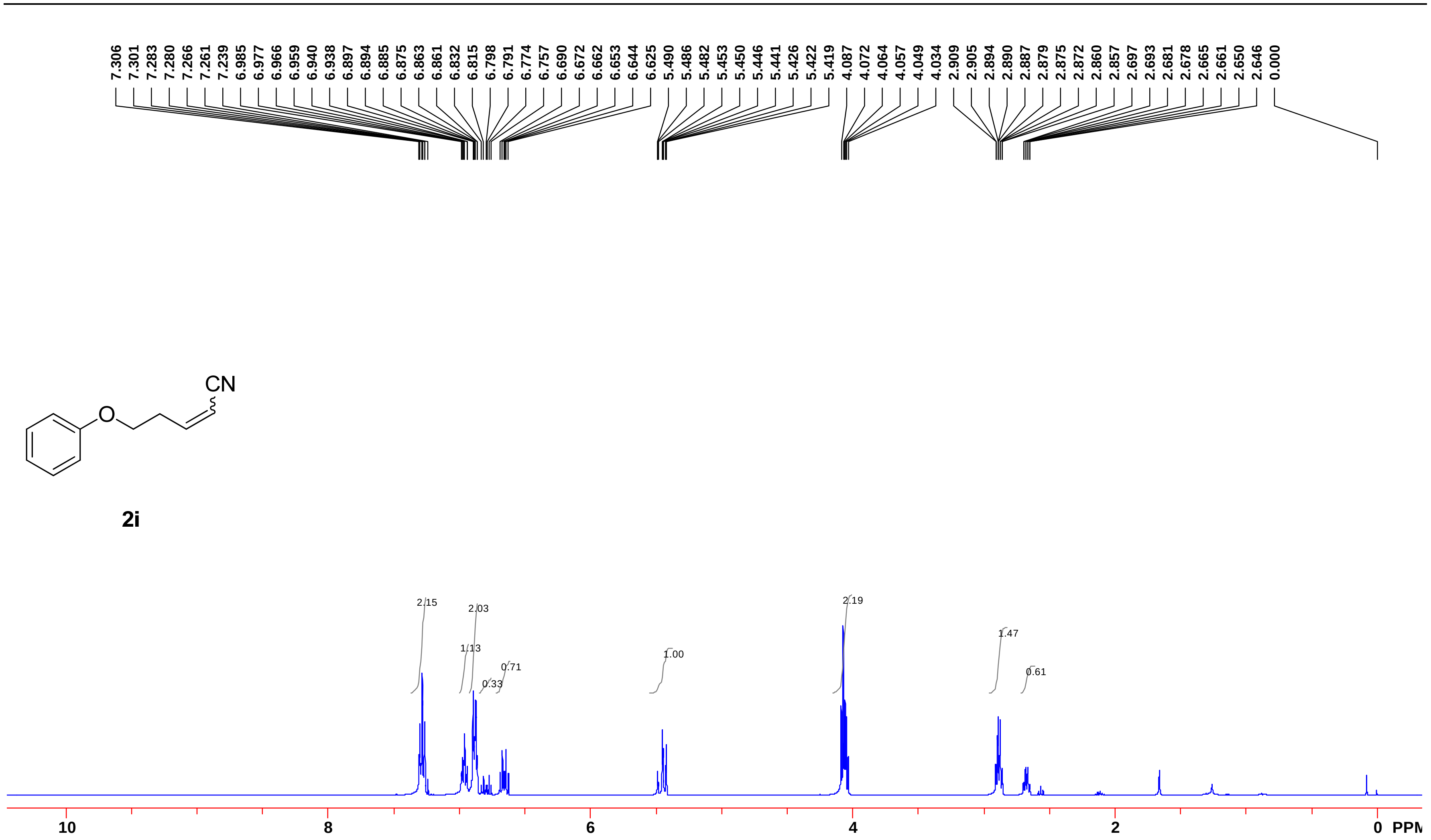
7.306 7.301 7.283 7.280 7.266 7.261 7.239 6.985 6.977 6.966 6.959 6.940 6.938 6.897 6.894 6.885 6.875 6.863 6.861 6.832 6.815 6.798 6.791 6.774 6.757 6.690 6.672 6.662 6.653 6.644 6.625 5.490 5.486 5.482 5.453 5.450 5.446 5.441 5.426 5.422 5.419 4.087 4.072 4.064 4.057 4.049 4.034 2.909 2.905 2.894 2.890 2.887 2.879 2.875 2.872 2.860 2.857 2.697 2.693 2.681 2.678 2.665 2.661 2.650 2.646 0.000
CN
O
2i
2.15
2.03
1.13
0.33
0.71
1.00
2.19
1.47
0.61
10
8
6
4
2
0 PPM

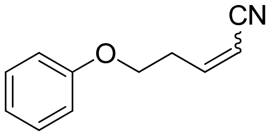
CN
O

2i

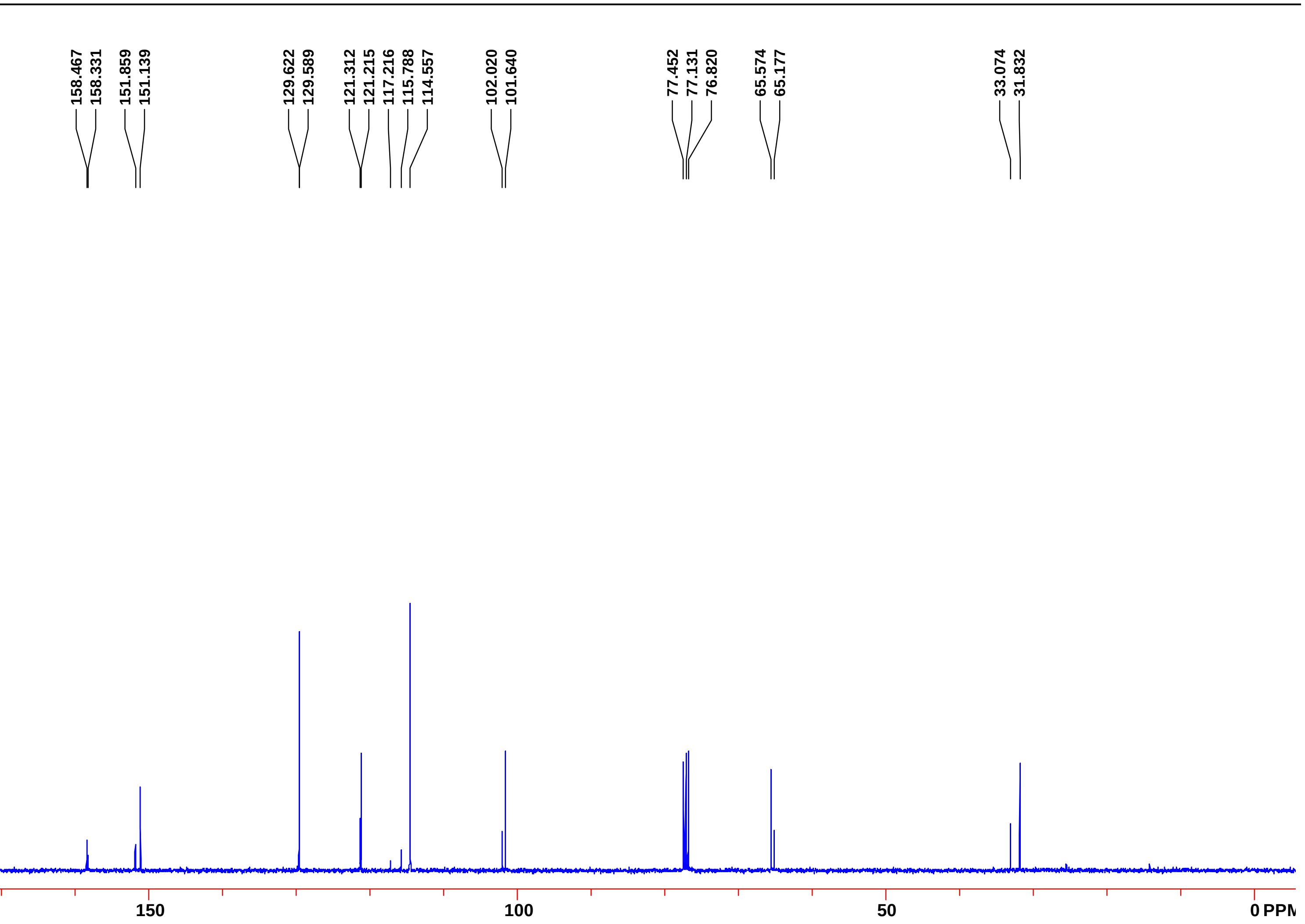
158.467
158.331
151.859
151.139
129.622
129.589
121.312
121.215
117.216
115.788
114.557
102.020
101.640
77.452
77.131
76.820
65.574
65.177
33.074
31.832
200
150
100
50
0 PPM

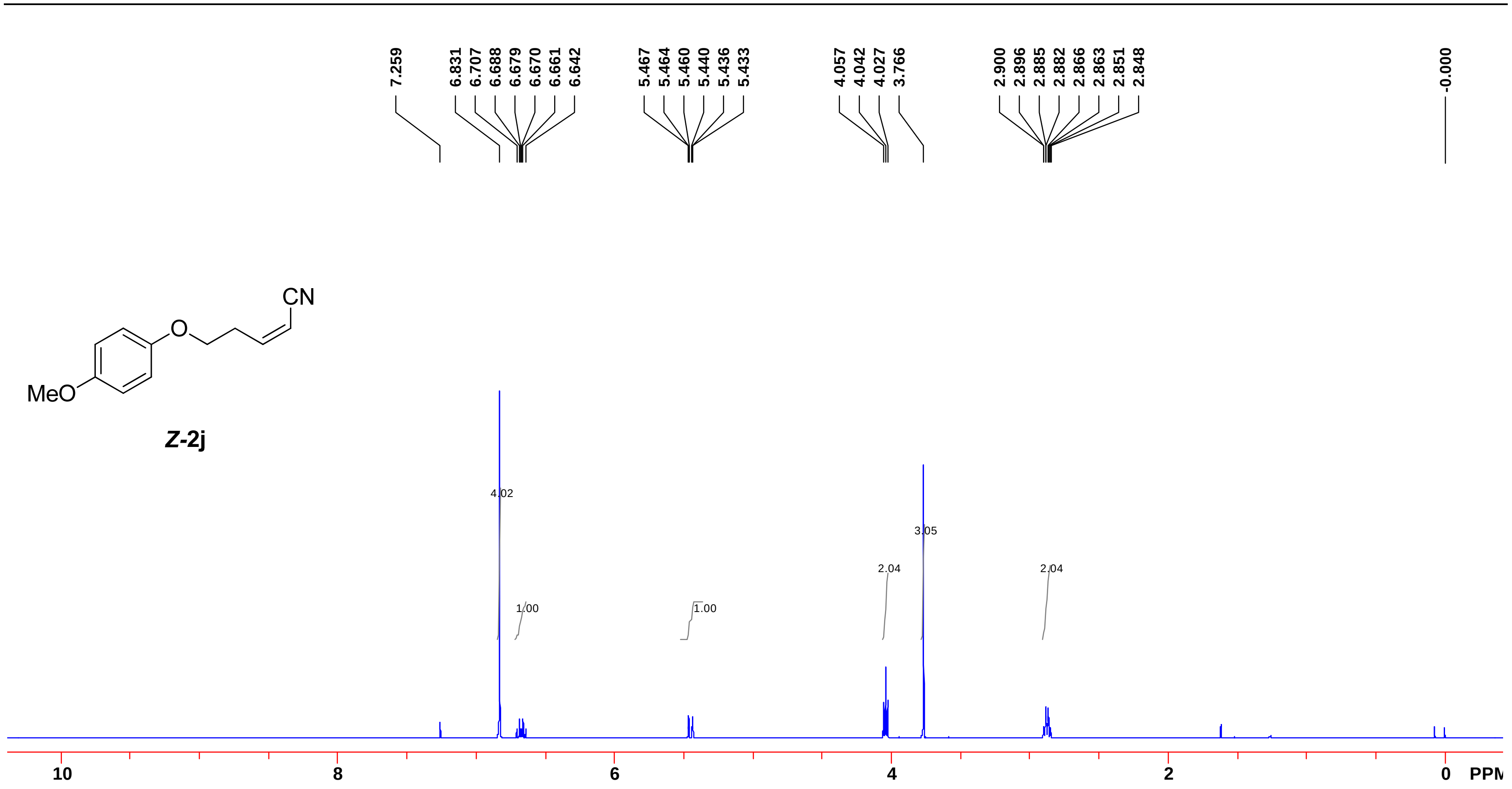
7.259
6.831
6.707
6.688
6.679
6.670
6.661
6.642
5.467
5.464
5.460
5.440
5.436
5.433
4.057
4.042
4.027
3.766
2.900
2.896
2.885
2.882
2.866
2.863
2.851
2.848
-0.000
CN
O
MeO
Z-2j
4.02
1.00
1.00
2.04
3.05
2.04
10
8
6
4
2
0
PPM

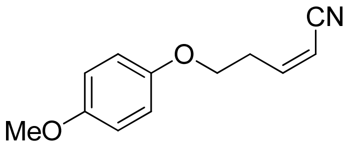
CN
O
MeO

***Z*-2j**

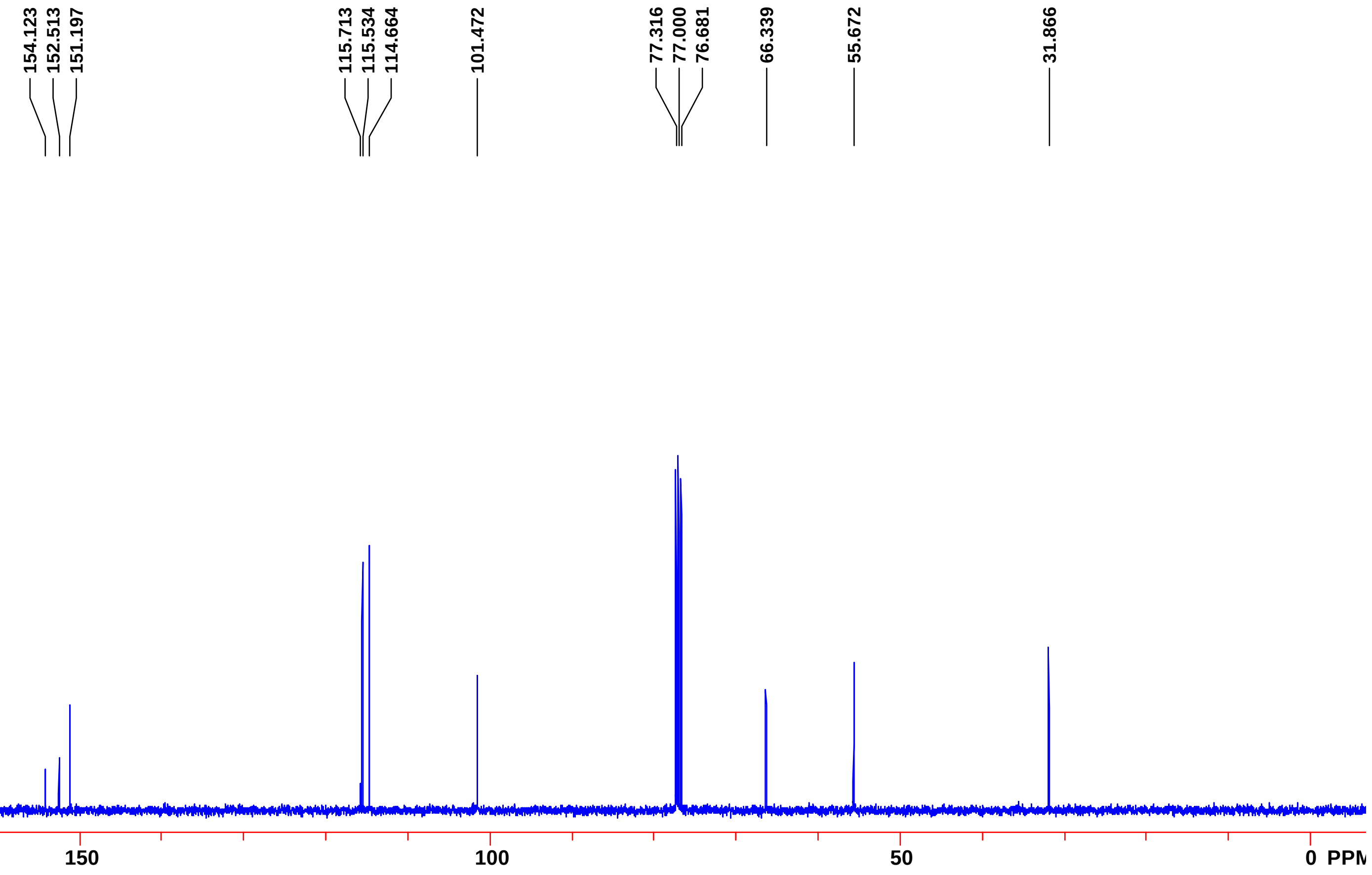
154.123
152.513
151.197
115.713
115.534
114.664
101.472
77.316
77.000
76.681
66.339
55.672
31.866
200
150
100
50
0 PPM

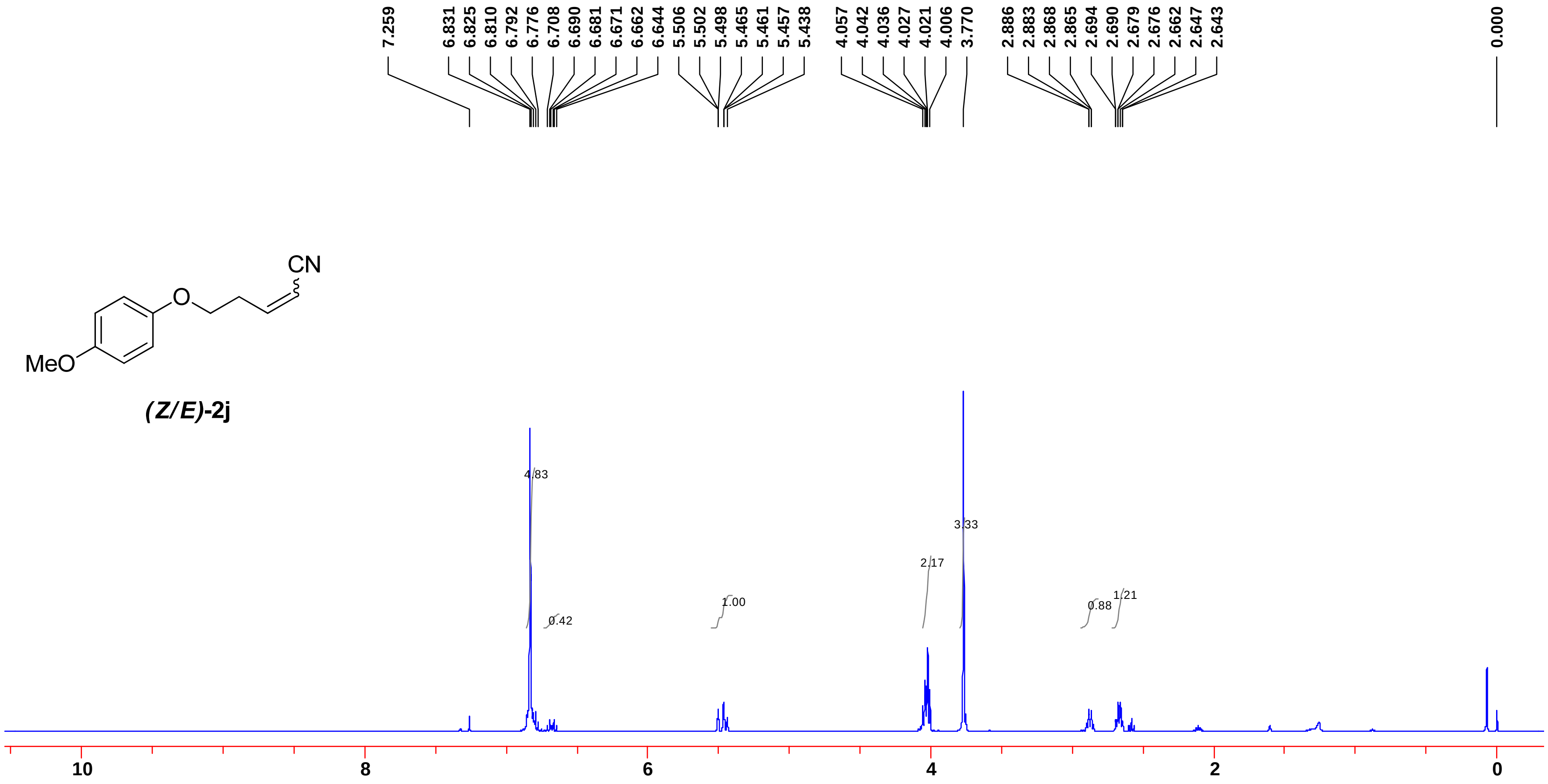
7.259
6.831
6.825
6.810
6.792
6.776
6.708
6.690
6.681
6.671
6.662
6.644
5.506
5.502
5.498
5.465
5.461
5.457
5.438
4.057
4.042
4.036
4.027
4.021
4.006
3.770
2.886
2.883
2.868
2.865
2.694
2.690
2.679
2.676
2.662
2.647
2.643
0.000
CN
O
MeO
4.83
0.42
1.00
2.17
3.33
0.88
1.21
10
8
6
4
2
0

(Z/E)-2j

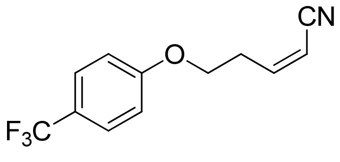

Z-2k

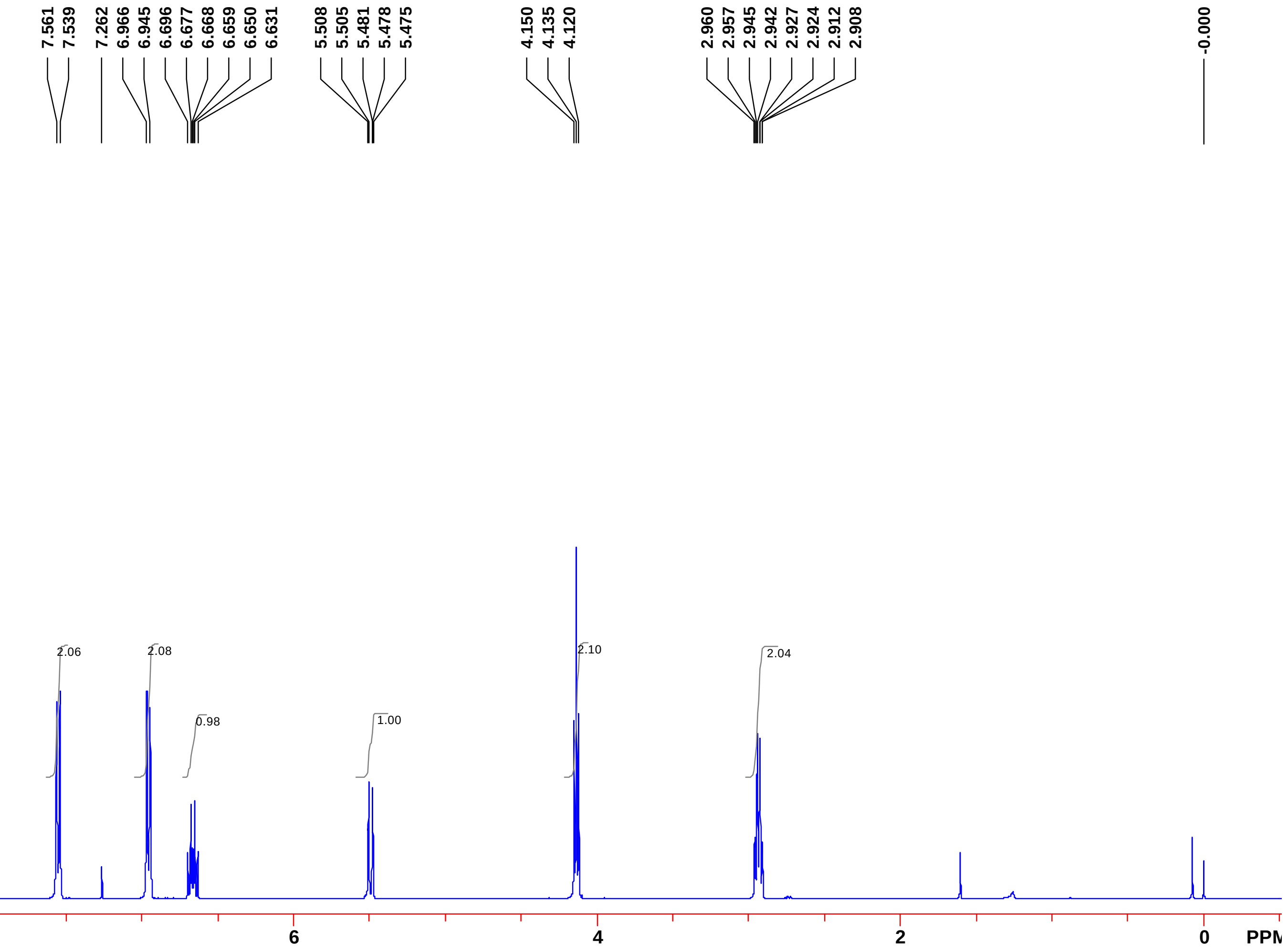
7.561
7.539
7.262
6.966
6.945
6.696
6.677
6.668
6.659
6.650
6.631
5.508
5.505
5.481
5.478
5.475
4.150
4.135
4.120
2.960
2.957
2.945
2.942
2.927
2.924
2.912
2.908
-0.000
2.06
2.08
0.98
1.00
2.10
2.04
10
8
6
4
2
0
PPM

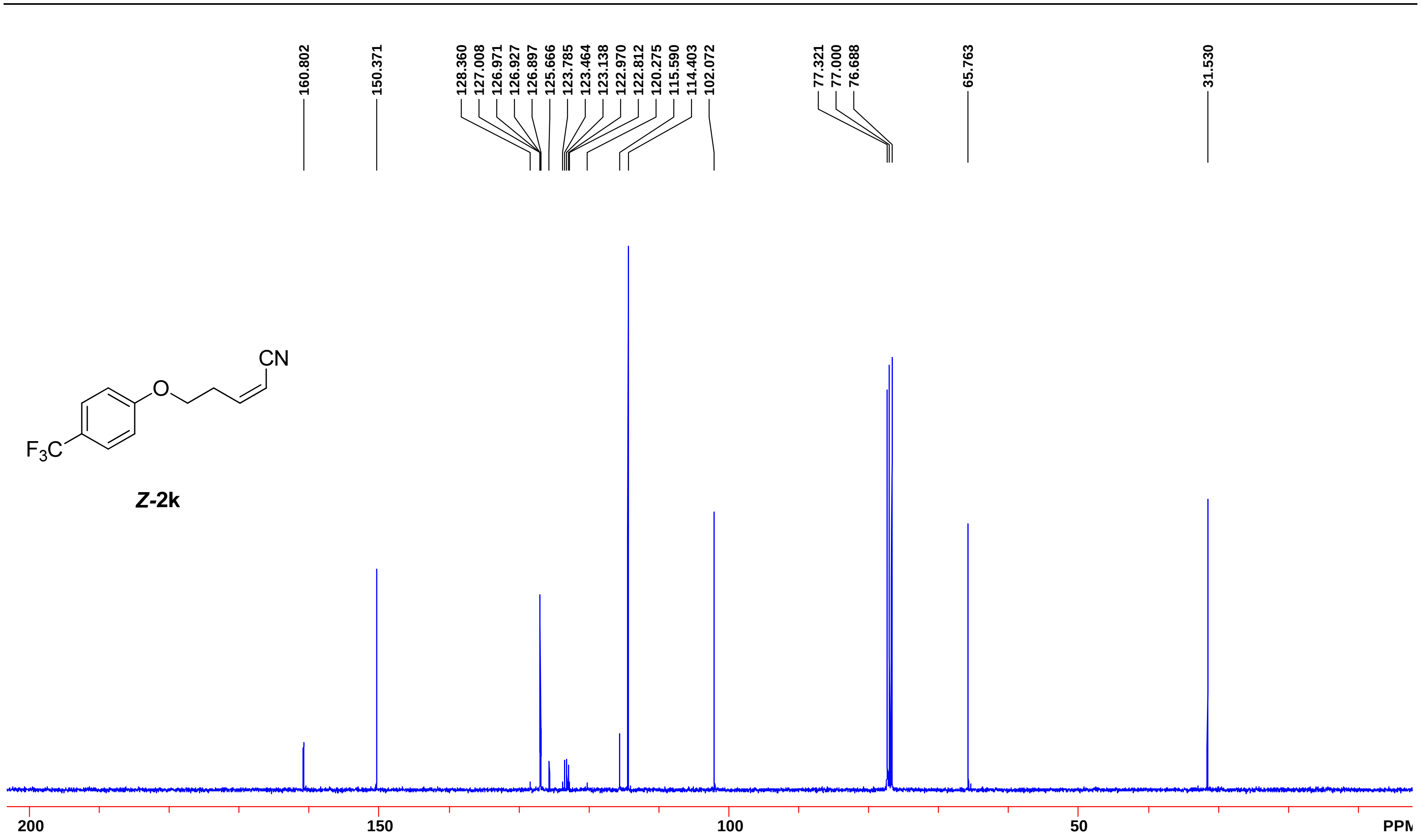
160.802
150.371
128.360
127.008
126.971
126.927
126.897
125.666
123.785
123.464
123.138
122.970
122.812
120.275
115.590
114.403
102.072
77.321
77.000
76.688
65.763
31.530
CN
O
F_3C
Z-2k
200
150
100
50
PPM

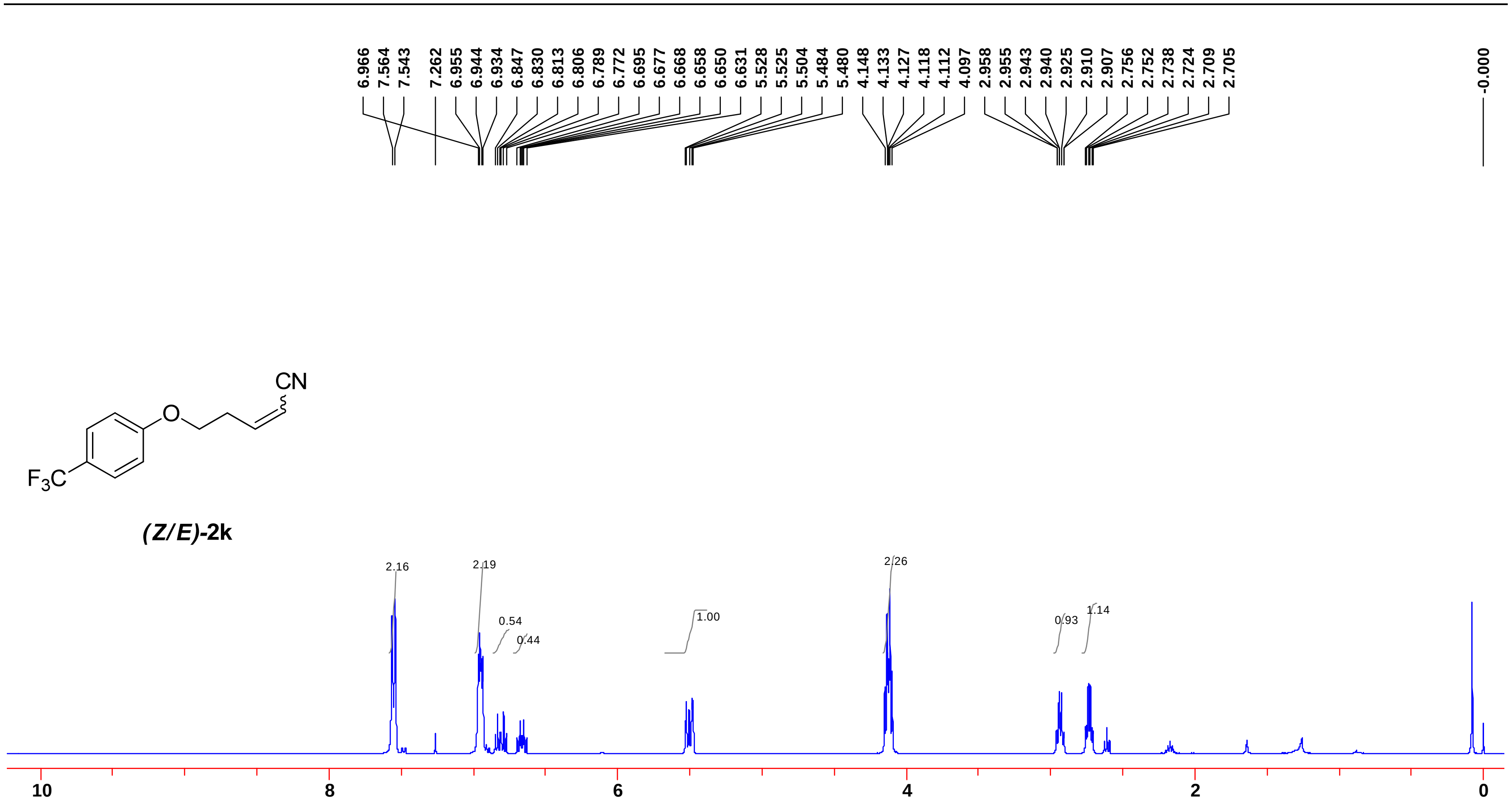
6.966
7.564
7.543
7.262
6.955
6.944
6.934
6.847
6.830
6.813
6.806
6.789
6.772
6.695
6.677
6.668
6.658
6.650
6.631
5.528
5.525
5.504
5.484
5.480
4.148
4.133
4.127
4.118
4.112
4.097
2.958
2.955
2.943
2.940
2.925
2.910
2.907
2.756
2.752
2.738
2.724
2.709
2.705
-0.000
CN
O
F3C
(Z/E)-2k
2.16
2.19
0.54
0.44
1.00
2.26
0.93
1.14
10
8
6
4
2
0

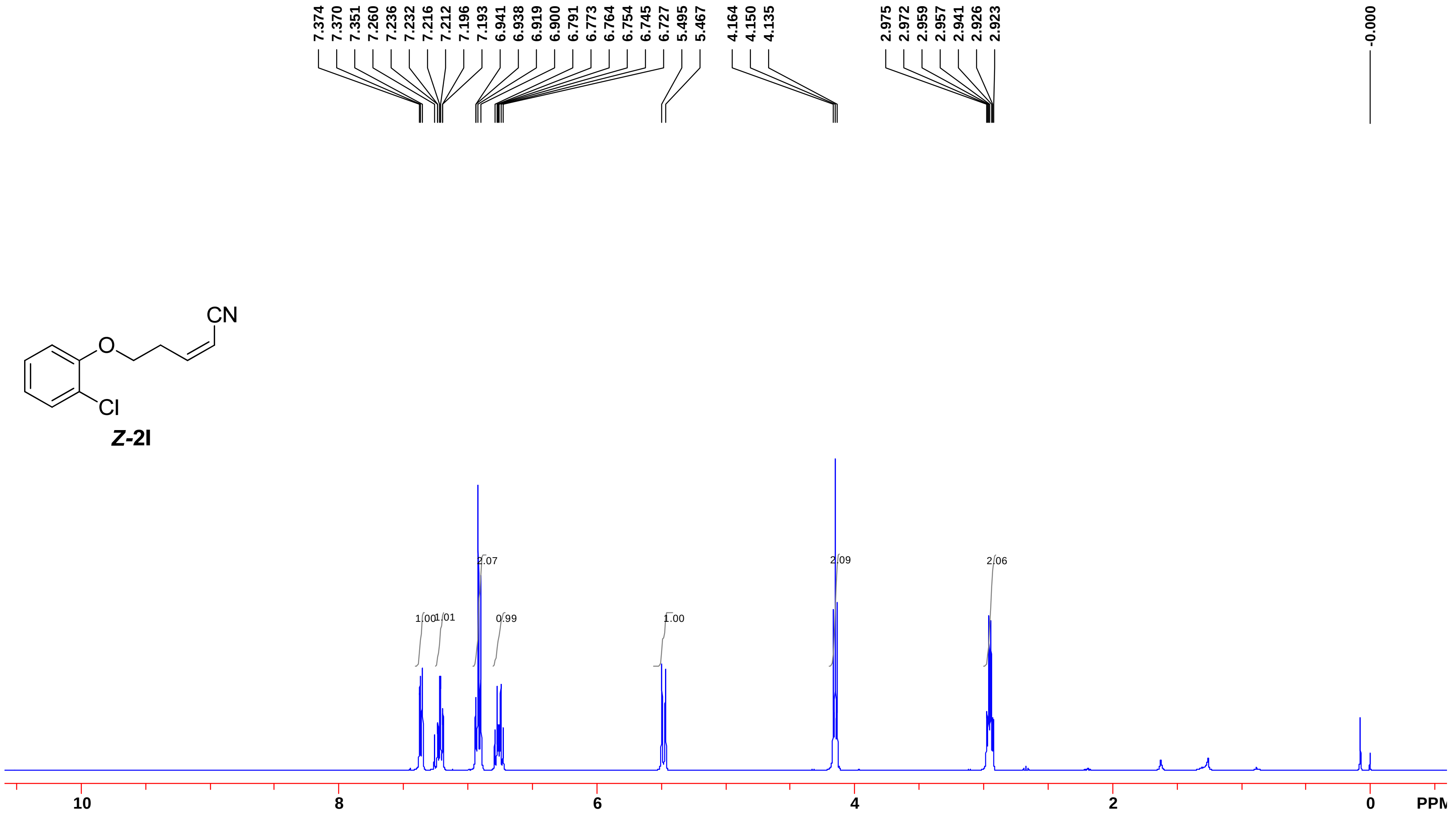
7.374
7.370
7.351
7.260
7.236
7.232
7.216
7.212
7.196
7.193
6.941
6.938
6.919
6.900
6.791
6.773
6.764
6.754
6.745
6.727
5.495
5.467
4.164
4.150
4.135
2.975
2.972
2.959
2.957
2.941
2.926
2.923
-0.000
CN
O
Cl
Z-2l
1.00
1.01
2.07
0.99
1.00
2.09
2.06
10
8
6
4
2
0
PPM

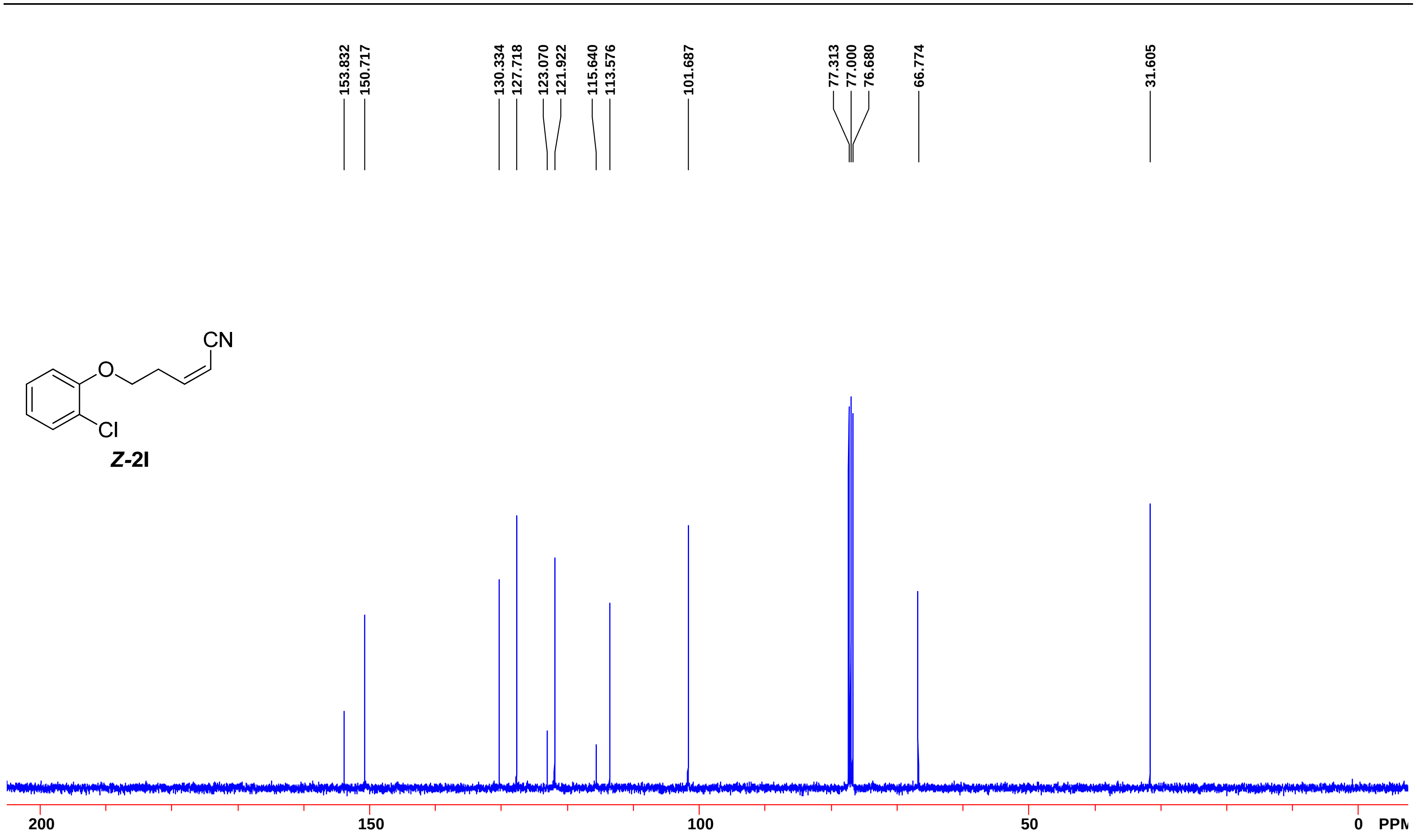
153.832
150.717
130.334
127.718
123.070
121.922
115.640
113.576
101.687
77.313
77.000
76.680
66.774
31.605
CN
O
Cl
Z-2l
200
150
100
50
0
PPM

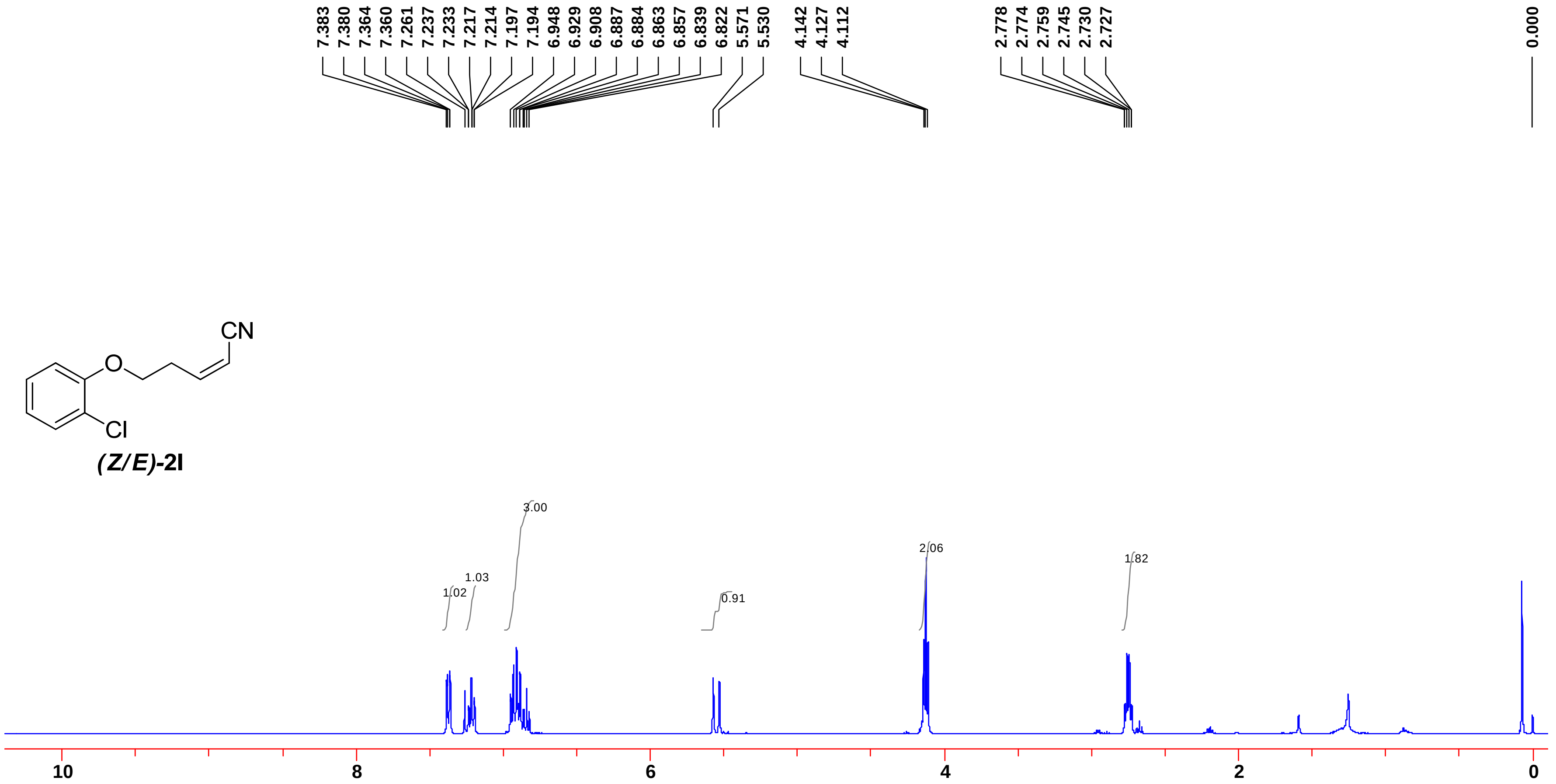
7.383
7.380
7.364
7.360
7.261
7.237
7.233
7.217
7.214
7.197
7.194
6.948
6.929
6.908
6.887
6.884
6.863
6.857
6.839
6.822
5.571
5.530
4.142
4.127
4.112
2.778
2.774
2.759
2.745
2.730
2.727
0.000
CN
O
Cl
1.02
1.03
3.00
0.91
2.06
1.82
10
8
6
4
2
0

***(Z/E)*-2l**

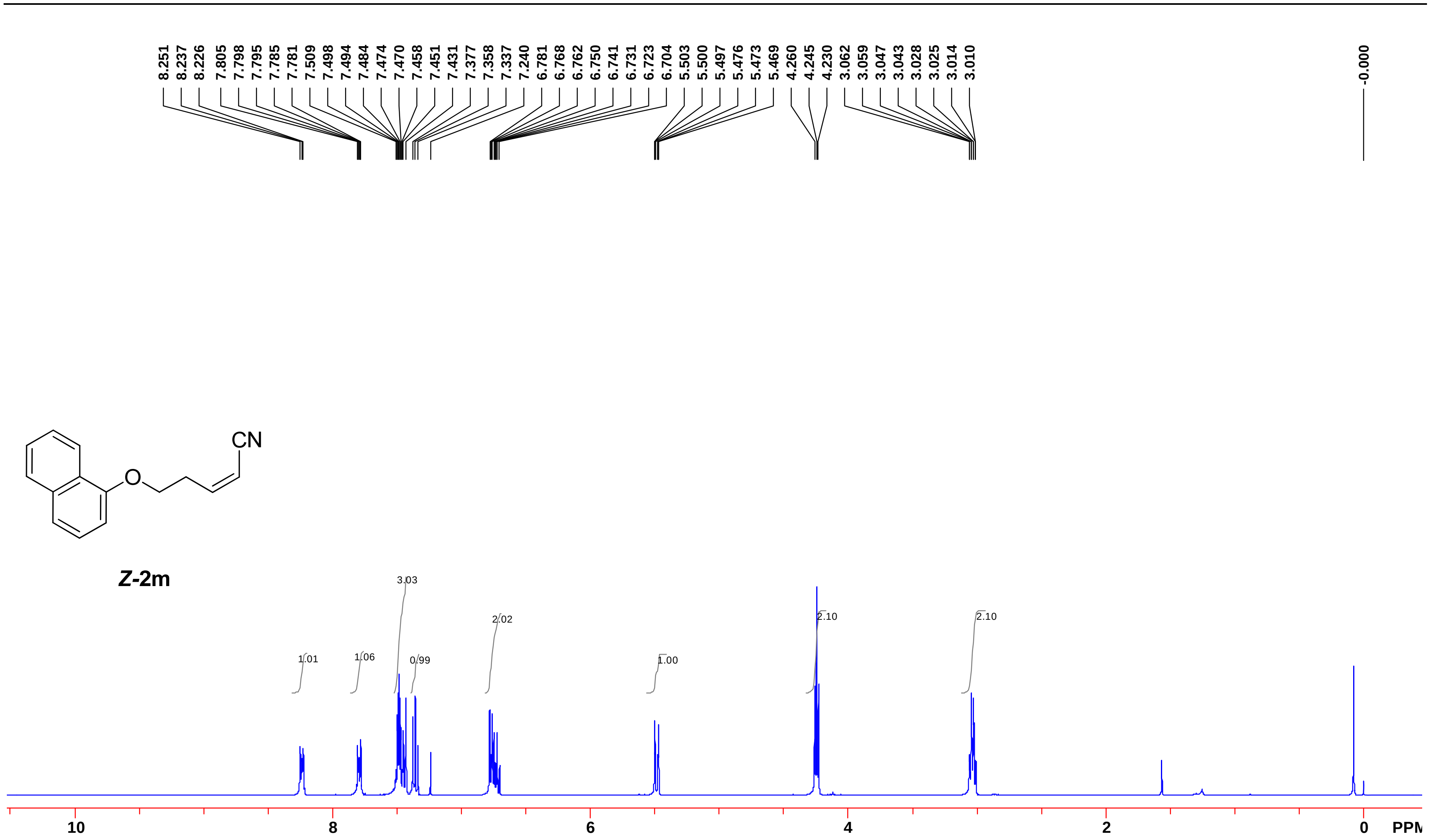
8.251 8.237 8.226 7.805 7.798 7.795 7.785 7.781 7.509 7.498 7.494 7.484 7.474 7.470 7.458 7.451 7.431 7.377 7.358 7.337 7.240 6.781 6.768 6.762 6.750 6.741 6.731 6.723 6.704 5.503 5.500 5.497 5.476 5.473 5.469 4.260 4.245 4.230 3.062 3.059 3.047 3.043 3.028 3.025 3.014 3.010
-0.000
CN
O
1.01
1.06
3.03
0.99
2.02
1.00
2.10
2.10
10
8
6
4
2
0
PPM

***Z*-2m**

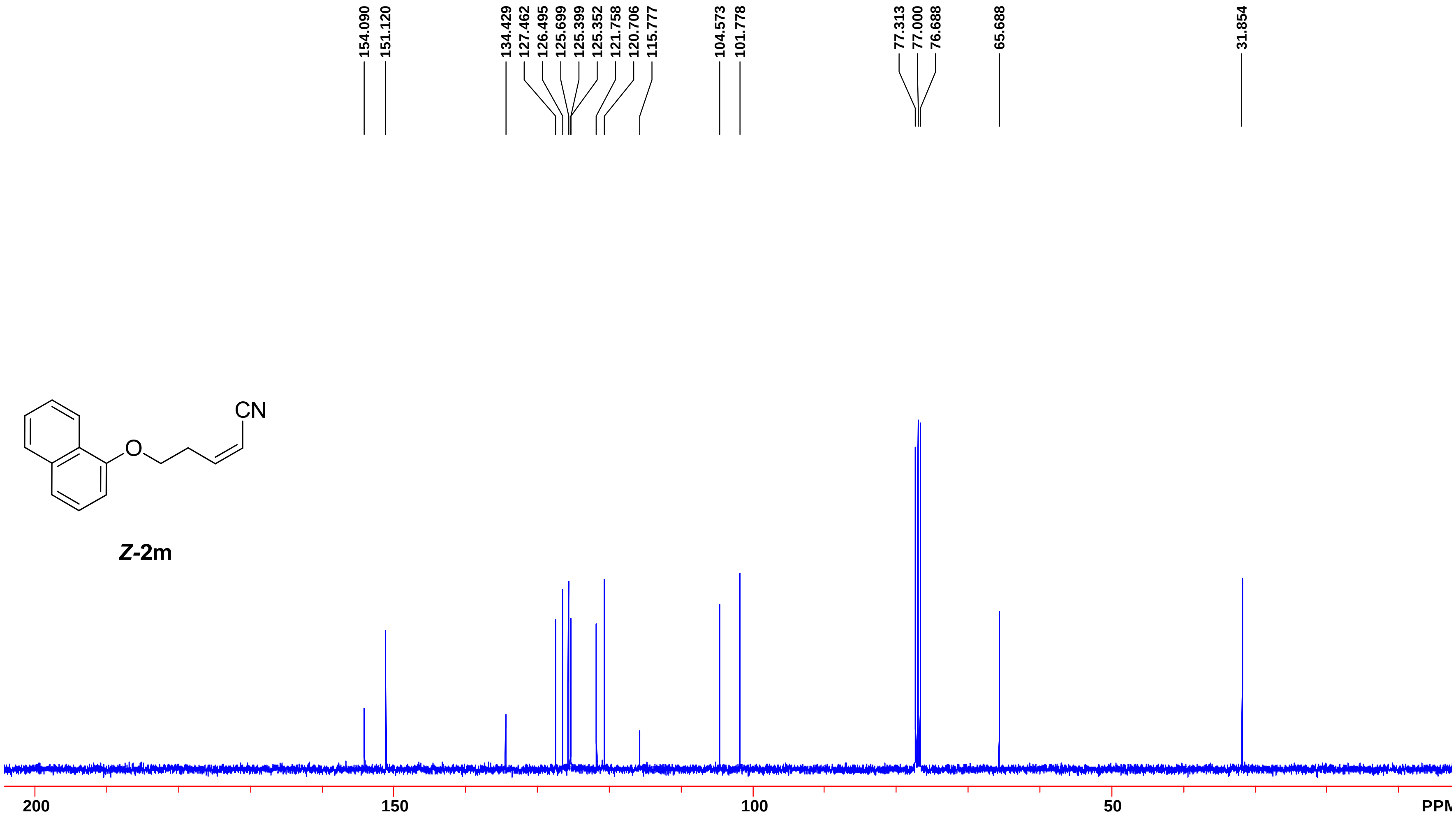
154.090
151.120
134.429
127.462
126.495
125.699
125.399
125.352
121.758
120.706
115.777
104.573
101.778
77.313
77.000
76.688
65.688
31.854
200
150
100
50
PPM
O
CN

***Z*-2m**

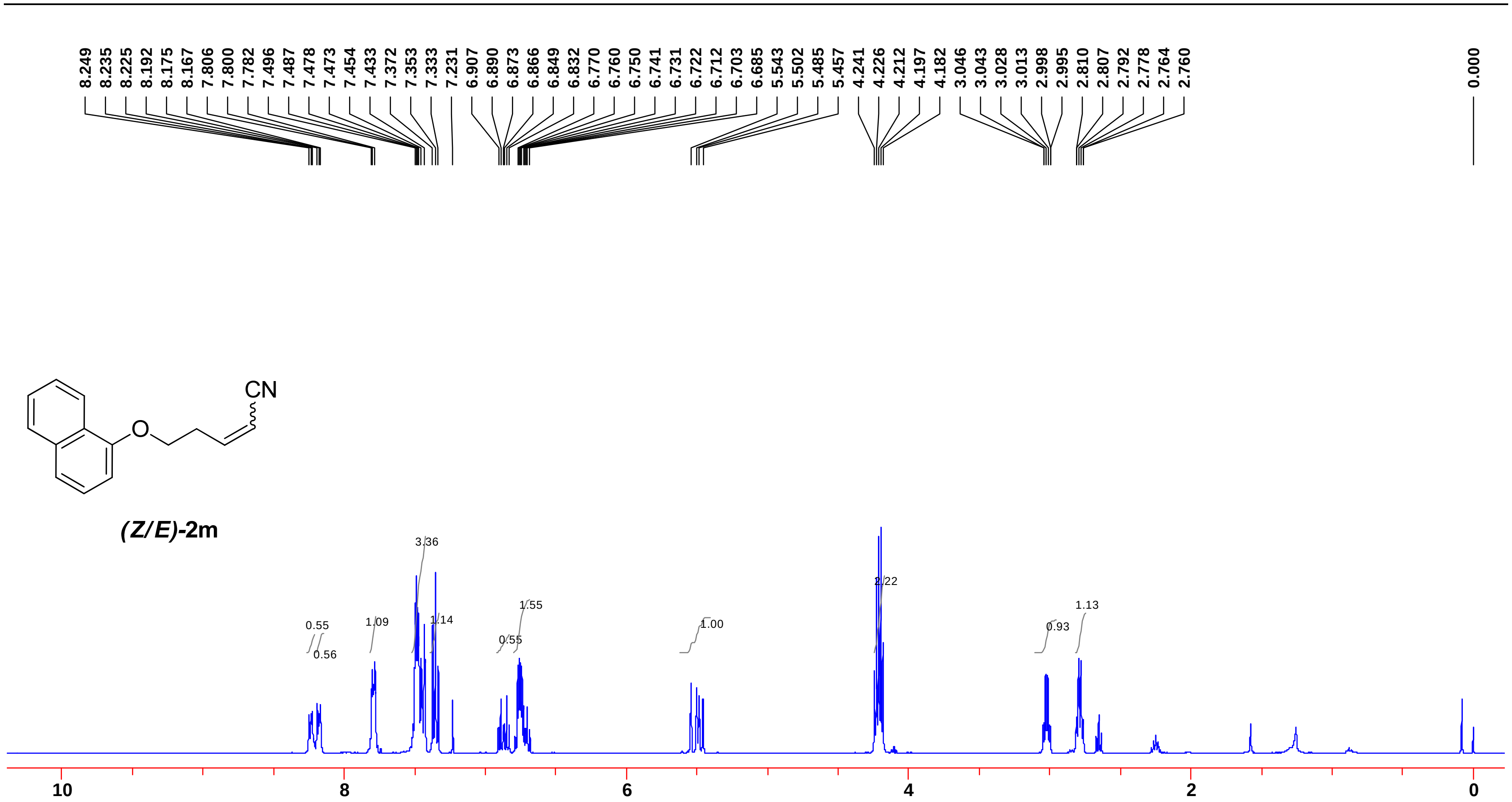
8.249 8.235 8.225 8.192 8.175 8.167 7.806 7.800 7.782 7.496 7.487 7.478 7.473 7.454 7.433 7.372 7.353 7.333 7.231 6.907 6.890 6.873 6.866 6.849 6.832 6.770 6.760 6.750 6.741 6.731 6.722 6.712 6.703 6.685 5.543 5.502 5.485 5.457 4.241 4.226 4.212 4.197 4.182 3.046 3.043 3.028 3.013 2.998 2.995 2.810 2.807 2.792 2.778 2.764 2.760 0.000
CN
O
(Z/E)-2m
0.55
0.56
1.09
3.36
1.14
0.55
1.55
1.00
2.22
0.93
1.13
10
8
6
4
2
0

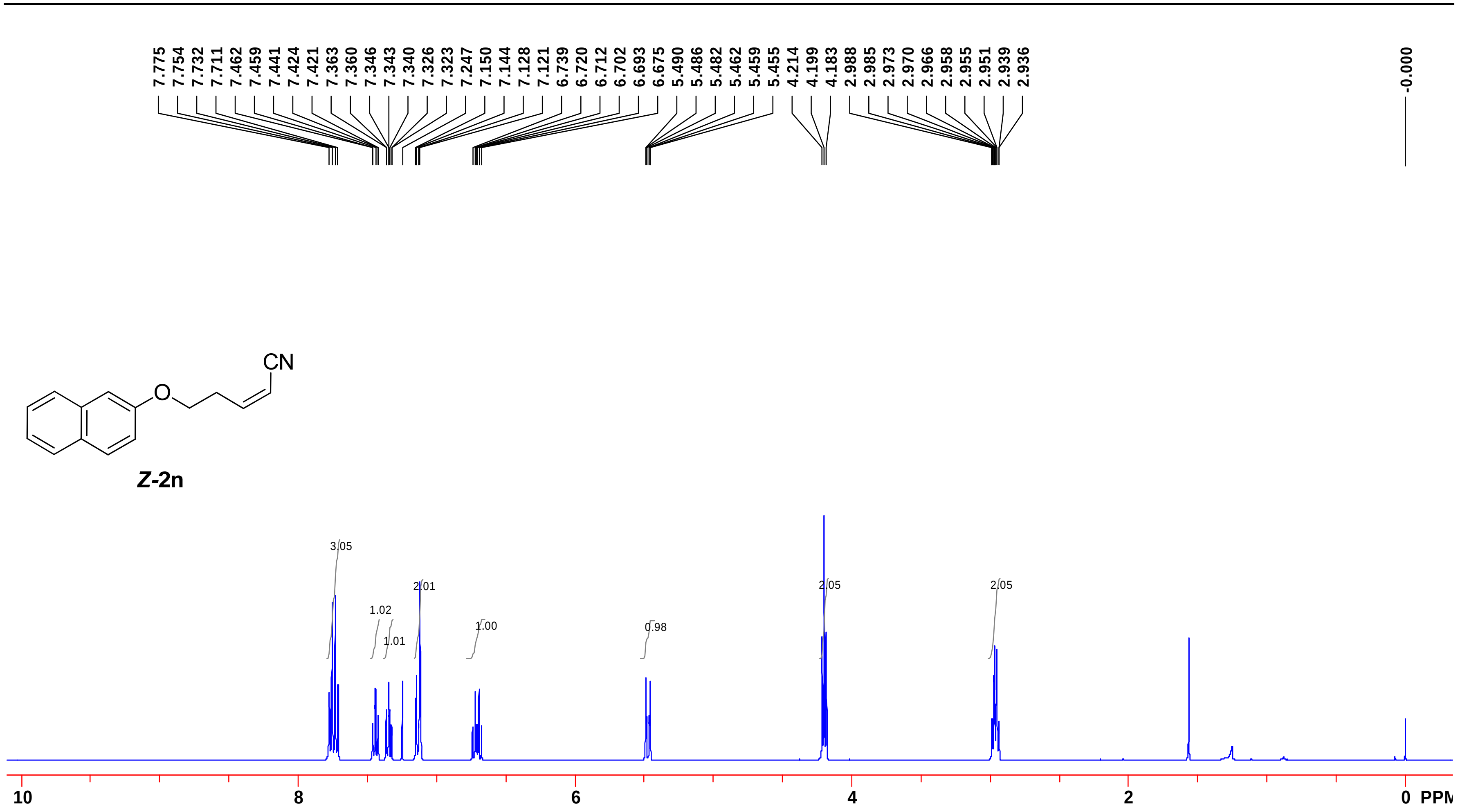
7.775 7.754 7.732 7.711 7.462 7.459 7.441 7.424 7.421 7.363 7.360 7.346 7.343 7.340 7.326 7.323 7.247 7.150 7.144 7.128 7.121 6.739 6.720 6.712 6.702 6.693 6.675 5.490 5.486 5.482 5.462 5.459 5.455 4.214 4.199 4.183 2.988 2.985 2.973 2.970 2.966 2.958 2.955 2.951 2.939 2.936
-0.000
CN
O
3.05
1.02
1.01
2.01
1.00
0.98
2.05
2.05
10
8
6
4
2
0 PPM

Z-2n

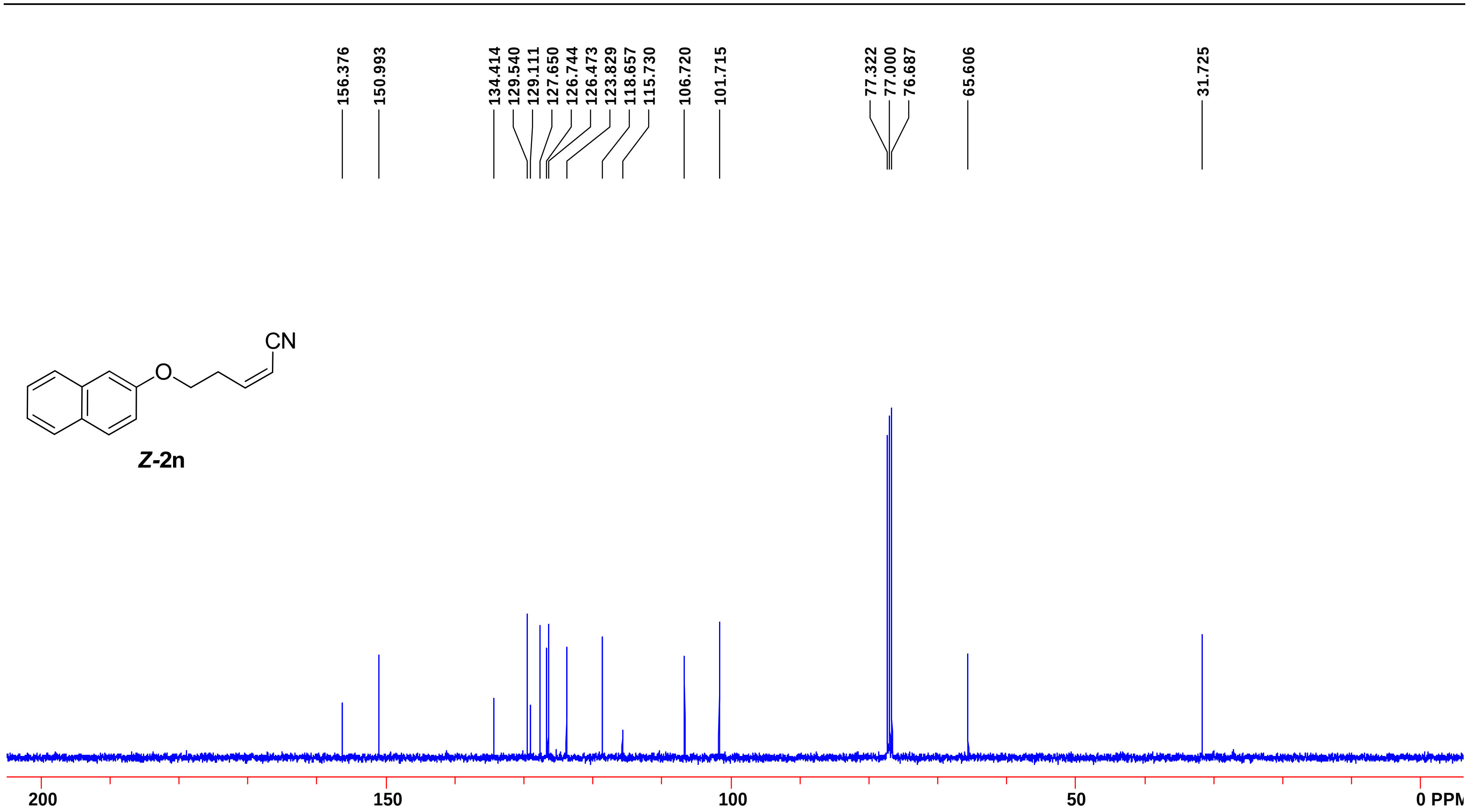
CN
O
156.376
150.993
134.414
129.540
129.111
127.650
126.744
126.473
123.829
118.657
115.730
106.720
101.715
77.322
77.000
76.687
65.606
31.725
200
150
100
50
0 PPM

***Z*-2n**

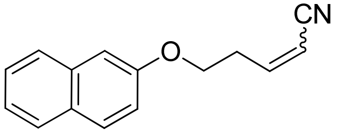
CN
O

(Z/E)-2n

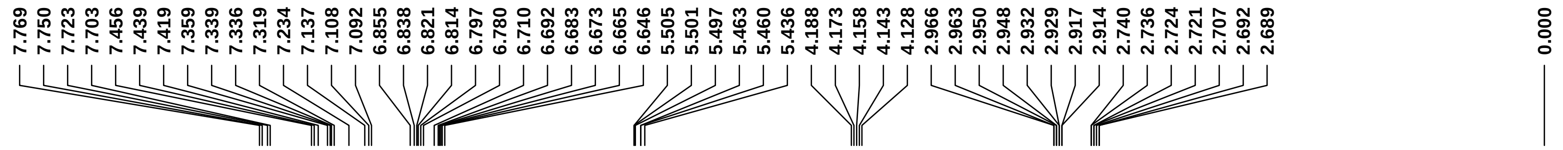
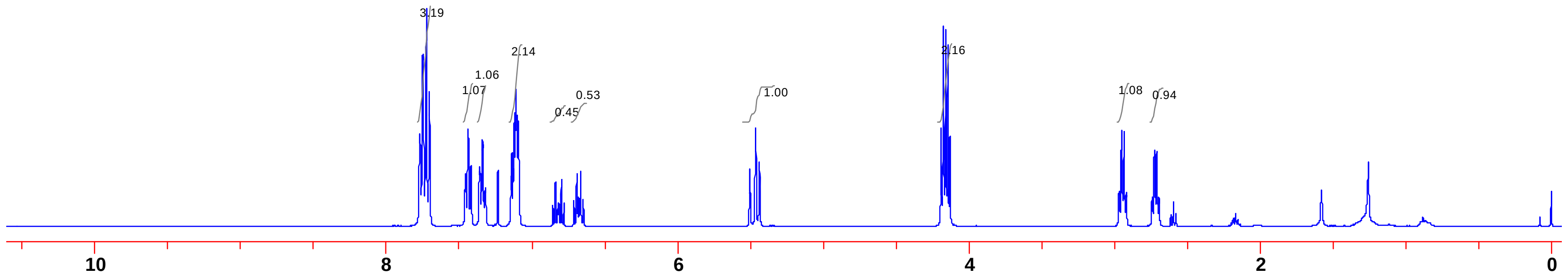
7.769 7.750 7.723 7.703 7.456 7.439 7.419 7.359 7.339 7.336 7.319 7.234 7.137 7.108 7.092 6.855 6.838 6.821 6.814 6.797 6.780 6.710 6.692 6.683 6.673 6.665 6.646 5.505 5.501 5.497 5.463 5.460 5.436 4.188 4.173 4.158 4.143 4.128 2.966 2.963 2.950 2.948 2.932 2.929 2.917 2.914 2.740 2.736 2.724 2.721 2.707 2.692 2.689
0.000
3.19
1.07
1.06
2.14
0.45
0.53
1.00
2.16
1.08
0.94
10
8
6
4
2
0

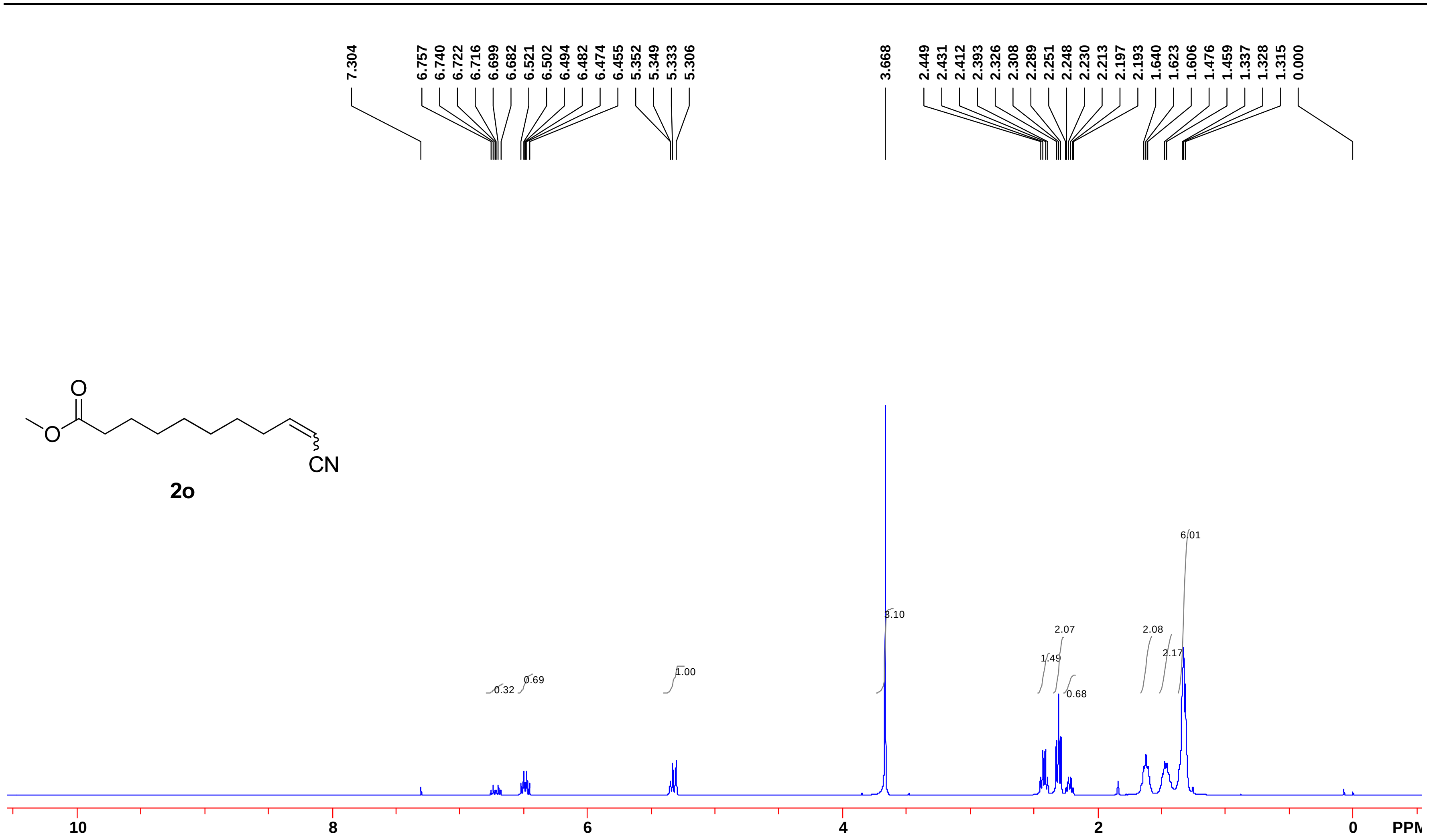
7.304
6.757
6.740
6.722
6.716
6.699
6.682
6.521
6.502
6.494
6.482
6.474
6.455
5.352
5.349
5.333
5.306
3.668
2.449
2.431
2.412
2.393
2.326
2.308
2.289
2.251
2.248
2.230
2.213
2.197
2.193
1.640
1.623
1.606
1.476
1.459
1.337
1.328
1.315
0.000
O
O
CN
2o
0.32
0.69
1.00
3.10
1.49
2.07
0.68
2.08
2.17
6.01
10
8
6
4
2
0
PPM

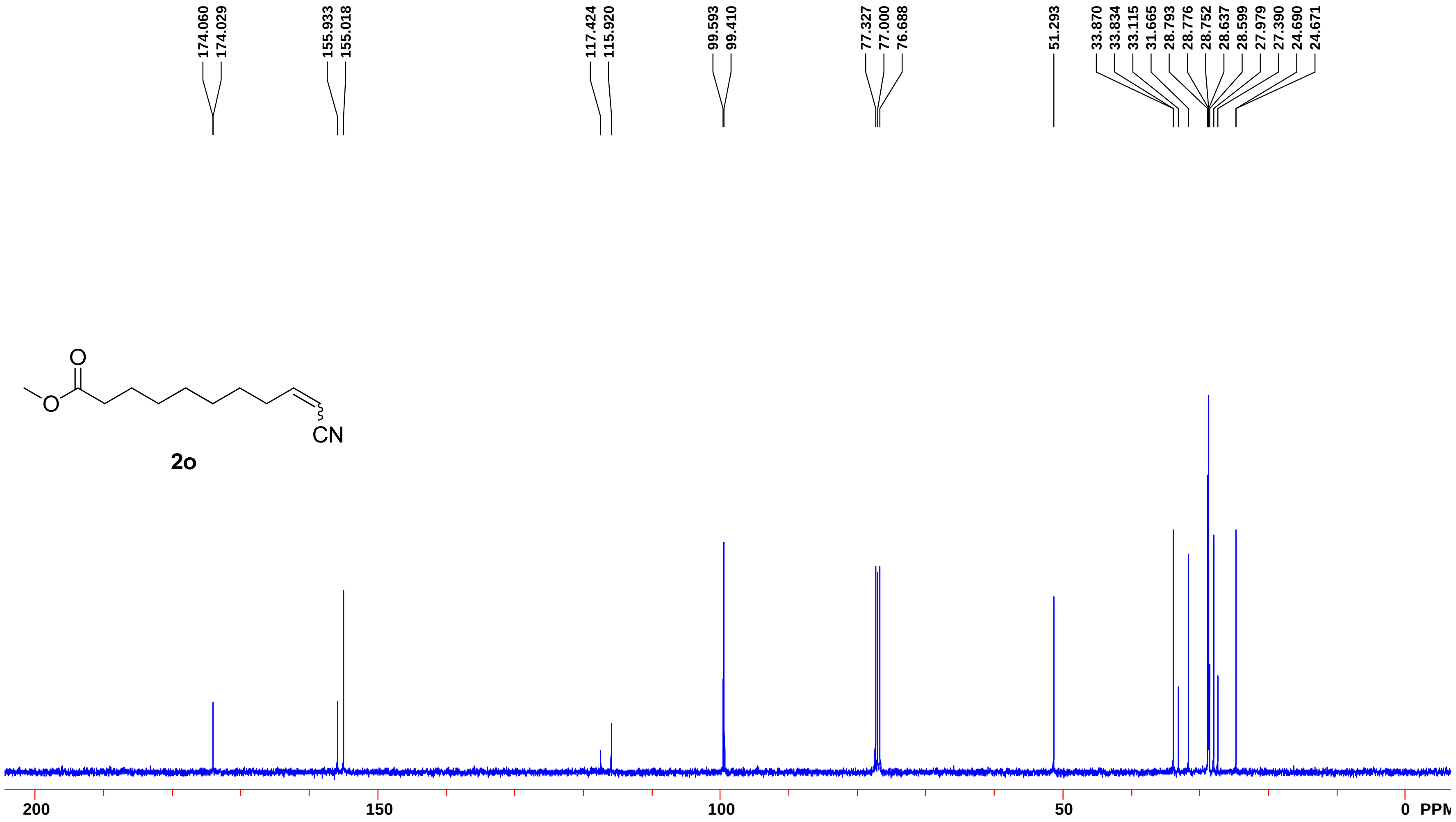
O
O
CN
174.060
174.029
155.933
155.018
117.424
115.920
99.593
99.410
77.327
77.000
76.688
51.293
33.870
33.834
33.115
31.665
28.793
28.776
28.752
28.637
28.599
27.979
27.390
24.690
24.671
200
150
100
50
0 PPM

2o

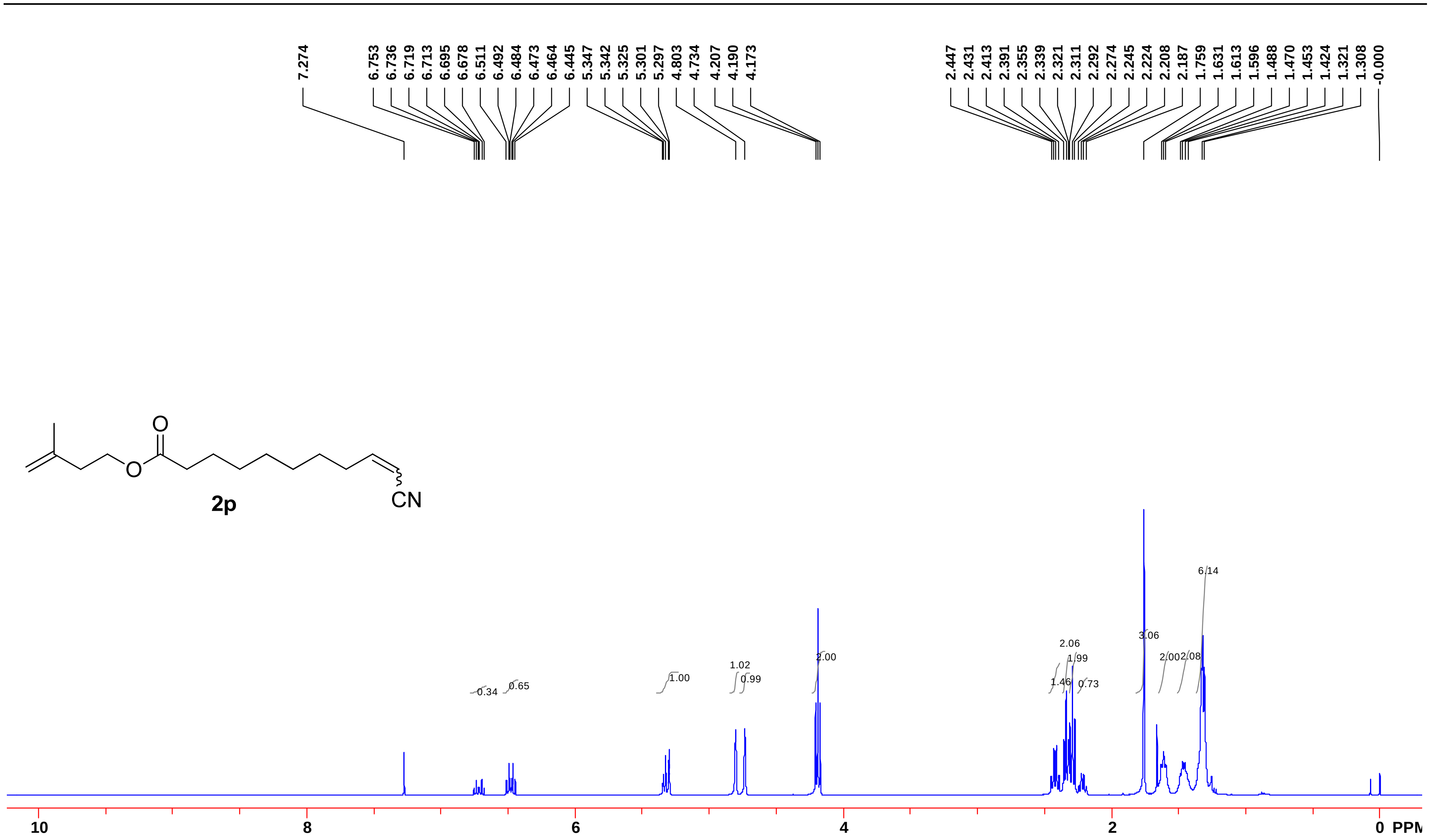

2p
CN
O
10
8
6
4
2
0 PPM

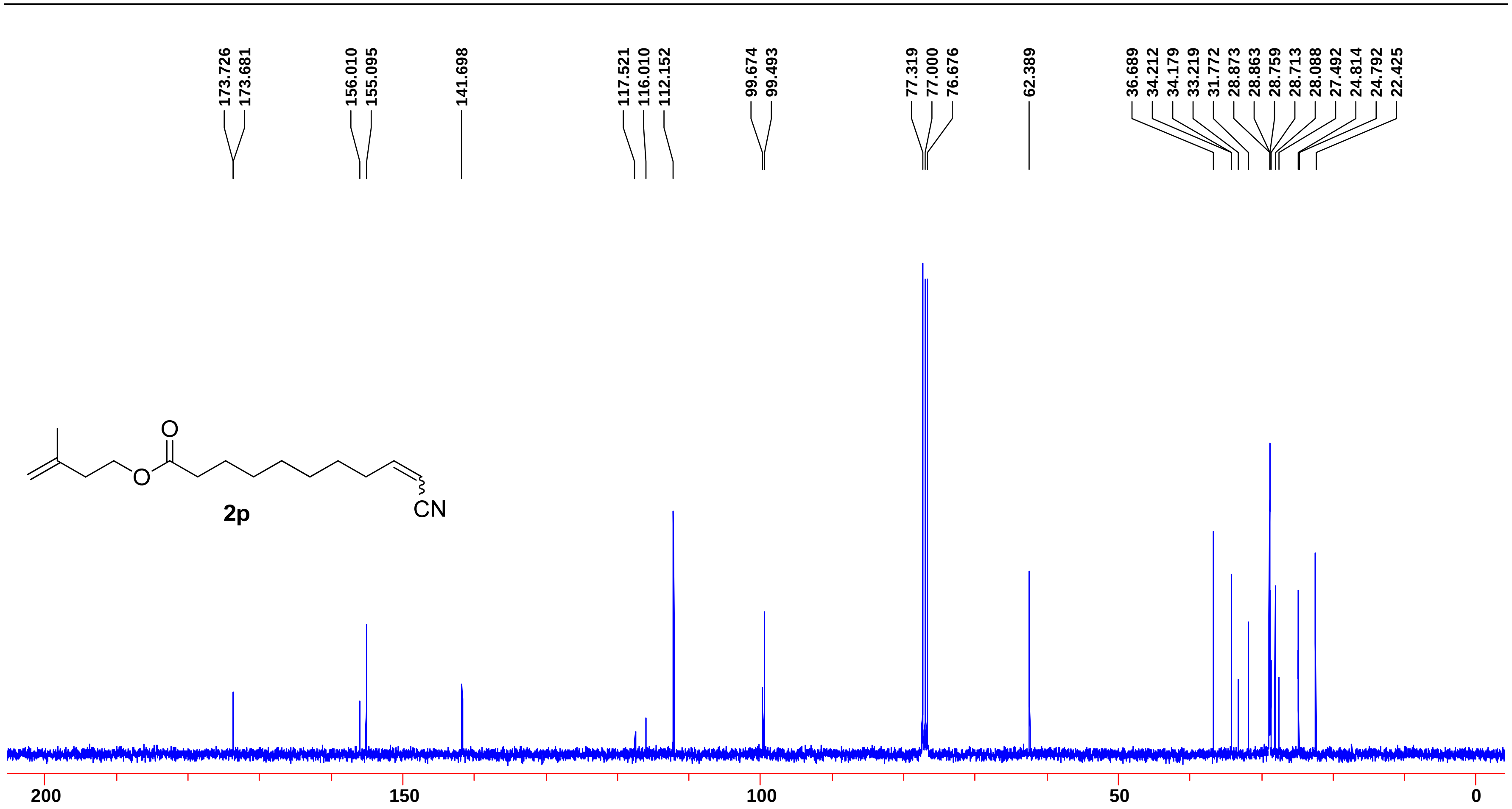
173.726
173.681
156.010
155.095
141.698
117.521
116.010
112.152
99.674
99.493
77.319
77.000
76.676
62.389
36.689
34.212
34.179
33.219
31.772
28.873
28.863
28.759
28.713
28.088
27.492
24.814
24.792
22.425
O
O
CN
2p
200
150
100
50
0

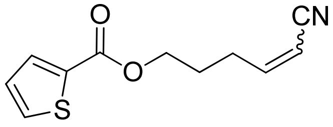
O
CN
O
S

2q

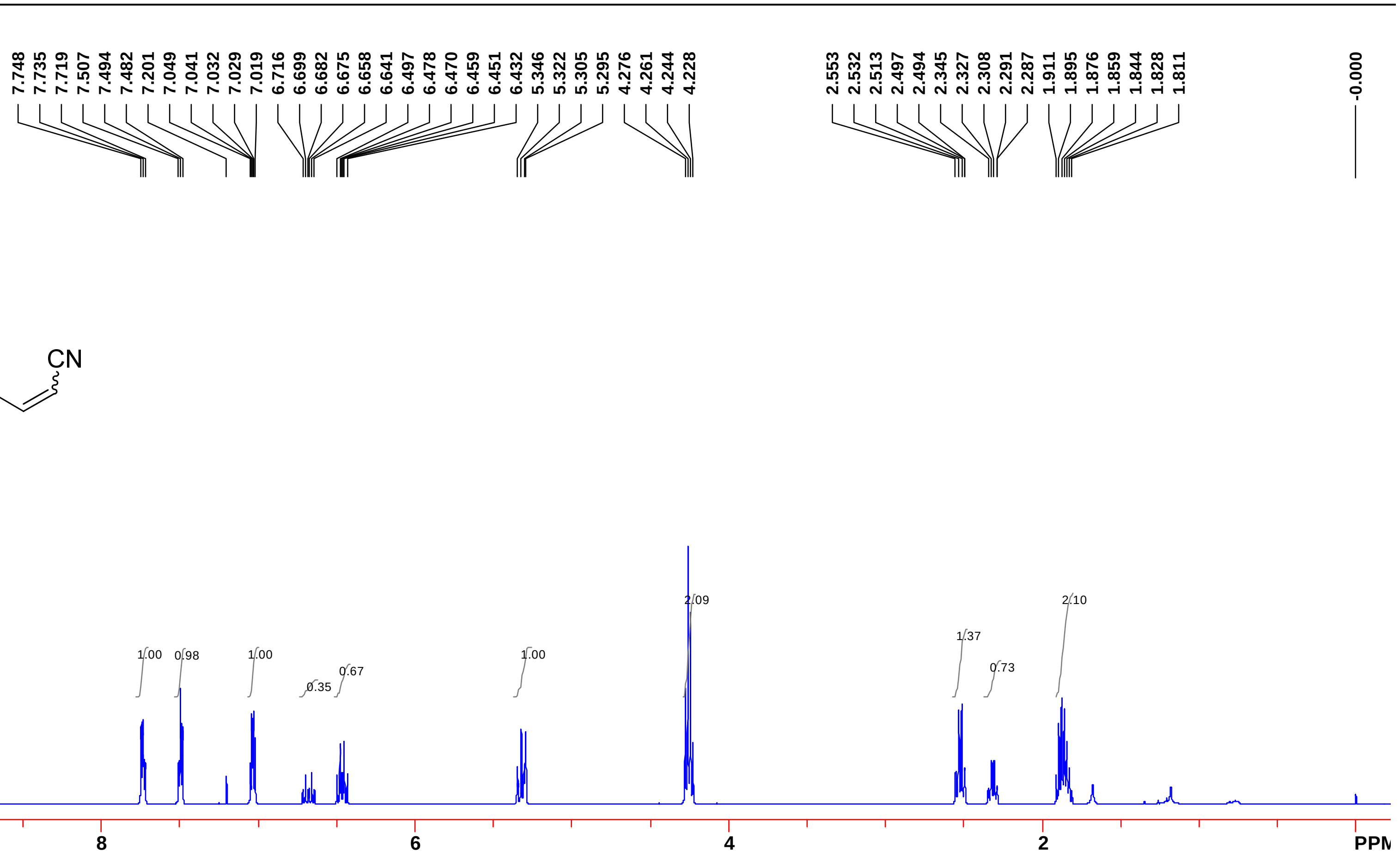
7.748
7.735
7.719
7.507
7.494
7.482
7.201
7.049
7.041
7.032
7.029
7.019
6.716
6.699
6.682
6.675
6.658
6.641
6.497
6.478
6.470
6.459
6.451
6.432
5.346
5.322
5.305
5.295
4.276
4.261
4.244
4.228
2.553
2.532
2.513
2.497
2.494
2.345
2.327
2.308
2.291
2.287
1.911
1.895
1.876
1.859
1.844
1.828
1.811
-0.000
1.00
0.98
1.00
0.35
0.67
1.00
2.09
1.37
0.73
2.10
10
8
6
4
2
PPM

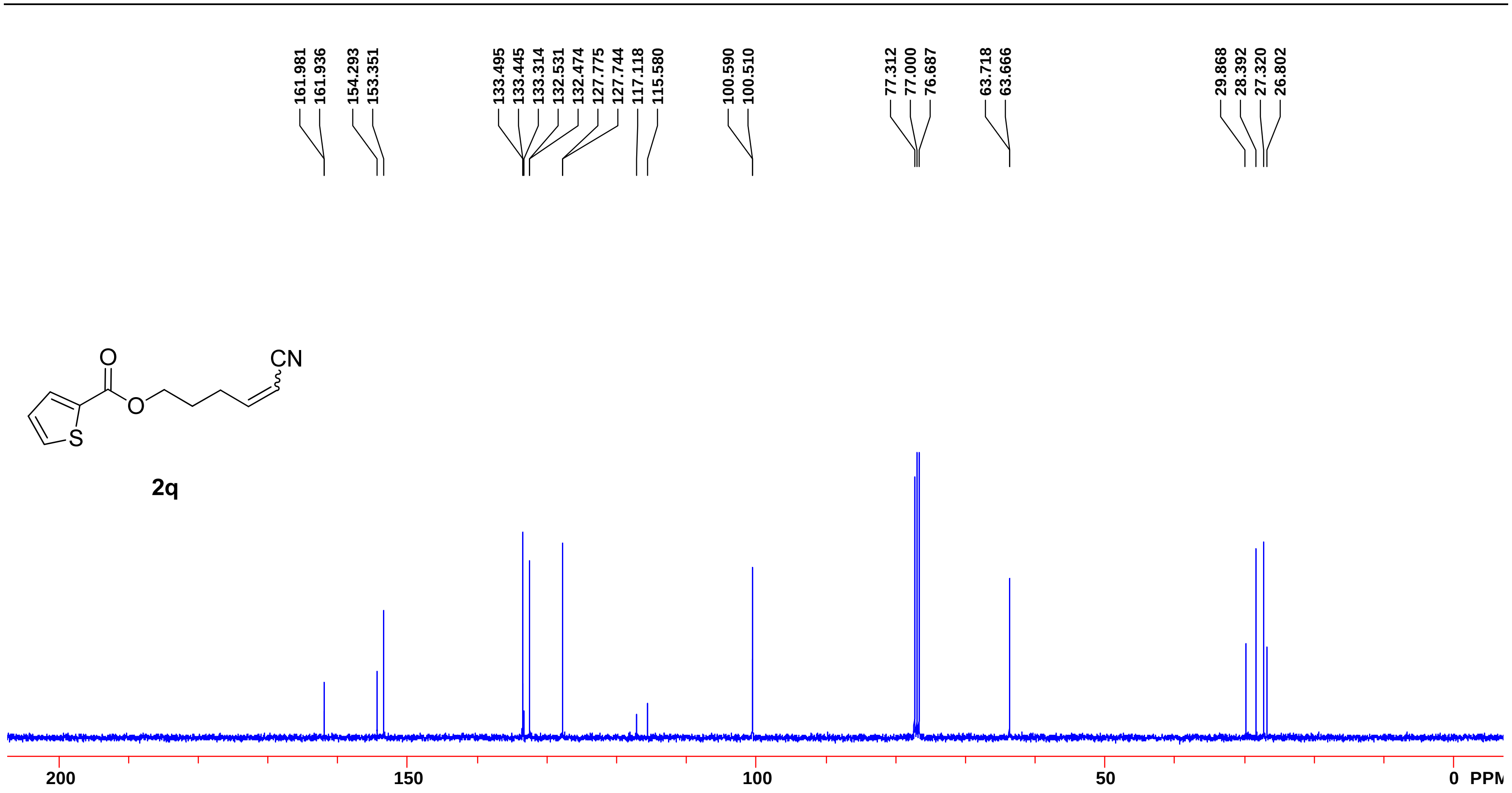
161.981
161.936
154.293
153.351
133.495
133.445
133.314
132.531
132.474
127.775
127.744
117.118
115.580
100.590
100.510
77.312
77.000
76.687
63.718
63.666
29.868
28.392
27.320
26.802
200
150
100
50
0 PPM
O
CN
O
S

2q

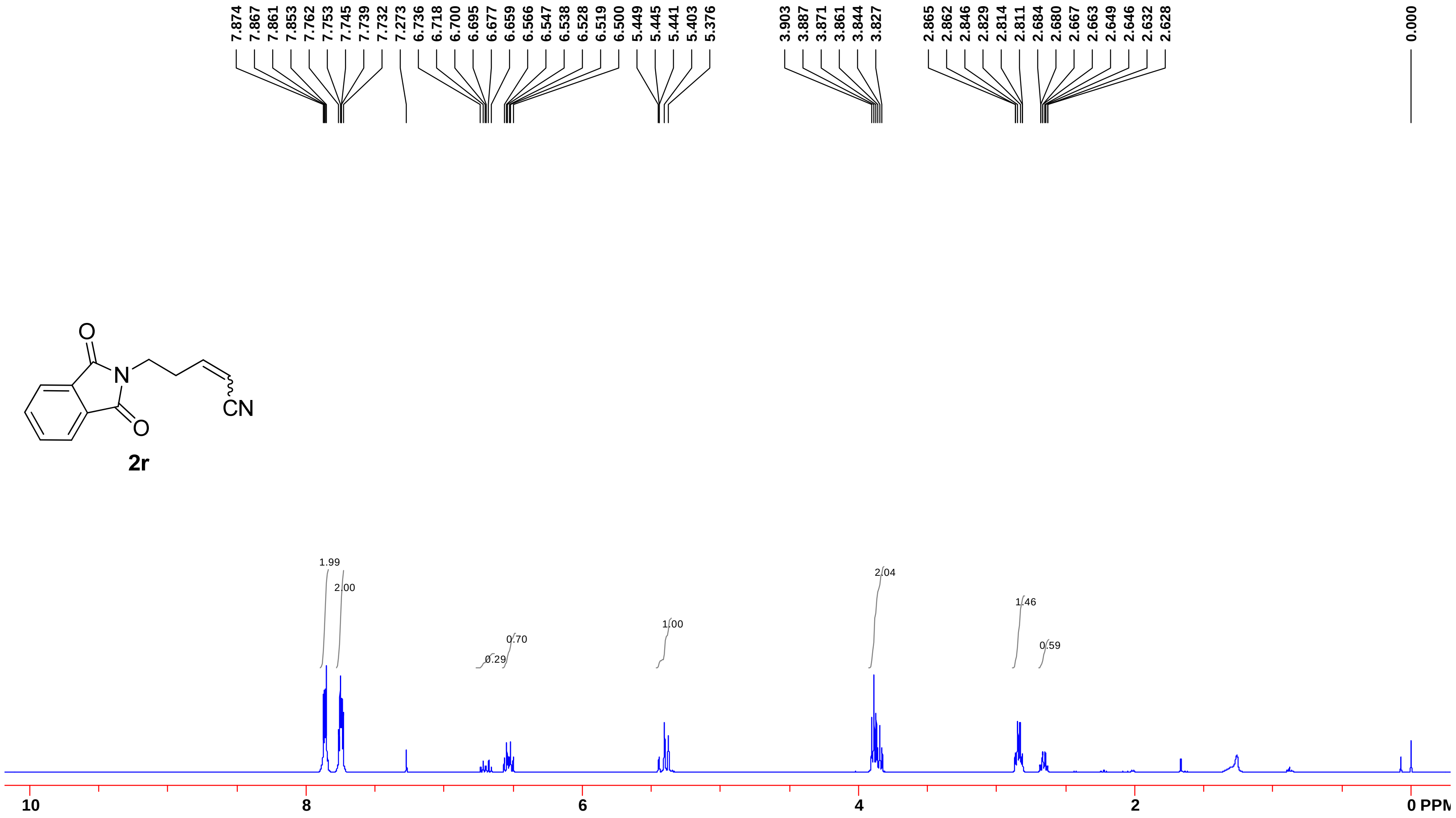
7.874
7.867
7.861
7.853
7.762
7.753
7.745
7.739
7.732
7.273
6.736
6.718
6.700
6.695
6.677
6.659
6.566
6.547
6.538
6.528
6.519
6.500
5.449
5.445
5.441
5.403
5.376
3.903
3.887
3.871
3.861
3.844
3.827
2.865
2.862
2.846
2.829
2.814
2.811
2.684
2.680
2.667
2.663
2.649
2.646
2.632
2.628
0.000
O
N
O
CN
2r
1.99
2.00
0.29
0.70
1.00
2.04
1.46
0.59
10
8
6
4
2
0 PPM

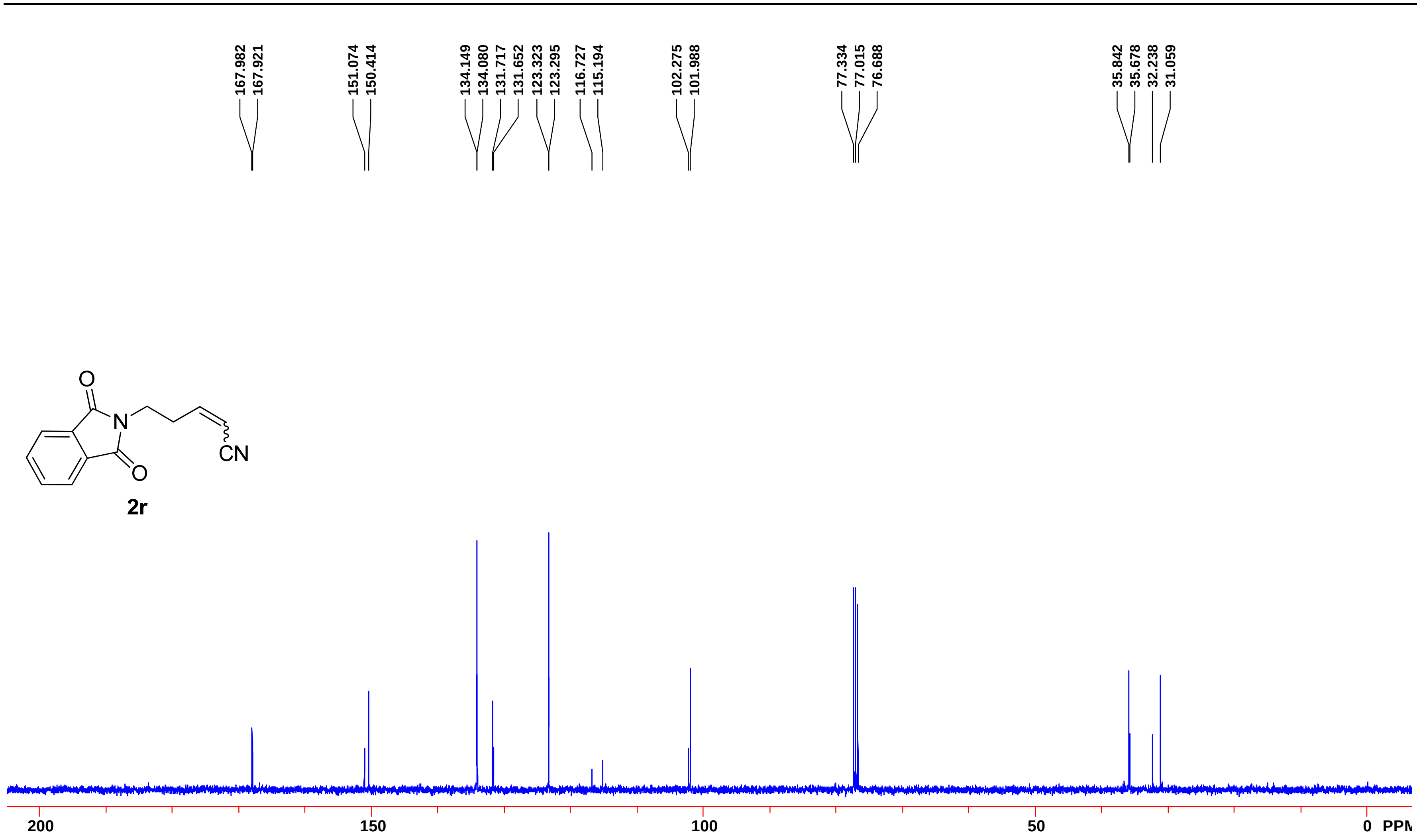
167.982
167.921
151.074
150.414
134.149
134.080
131.717
131.652
123.323
123.295
116.727
115.194
102.275
101.988
77.334
77.015
76.688
35.842
35.678
32.238
31.059
O
N
O
CN
2r
200
150
100
50
0 PPM

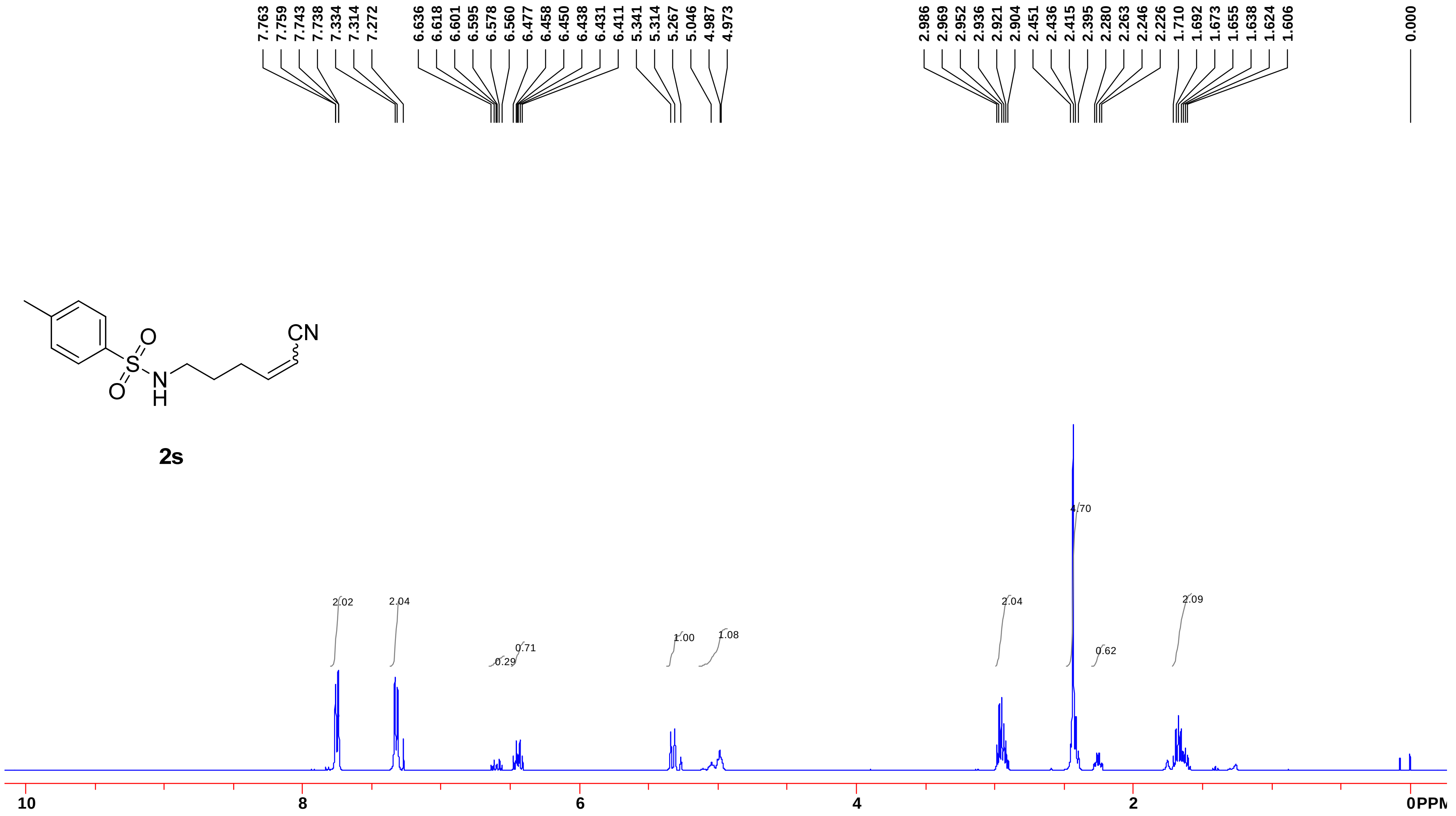
7.763
7.759
7.743
7.738
7.334
7.314
7.272
6.636
6.618
6.601
6.595
6.578
6.560
6.477
6.458
6.450
6.438
6.431
6.411
5.341
5.314
5.267
5.046
4.987
4.973
2.986
2.969
2.952
2.936
2.921
2.904
2.451
2.436
2.415
2.395
2.280
2.263
2.246
2.226
1.710
1.692
1.673
1.655
1.638
1.624
1.606
0.000
CN
O
S
O
N
H
2s
2.02
2.04
0.71
0.29
1.00
1.08
2.04
4.70
0.62
2.09
10
8
6
4
2
0 PPM

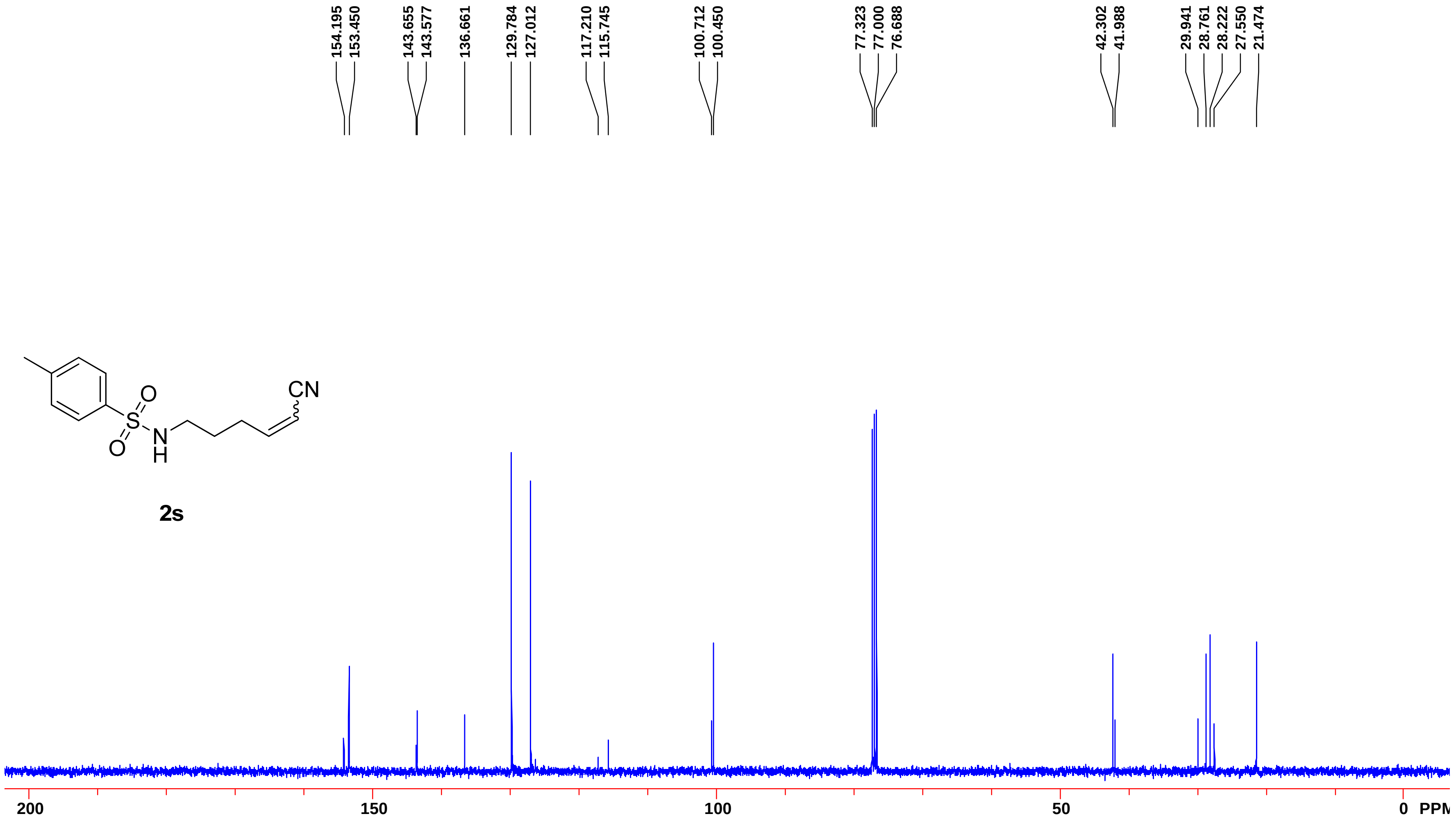
154.195
153.450
143.655
143.577
136.661
129.784
127.012
117.210
115.745
100.712
100.450
77.323
77.000
76.688
42.302
41.988
29.941
28.761
28.222
27.550
21.474
200
150
100
50
0
PPM
O
S
O
N
H
CN

2s

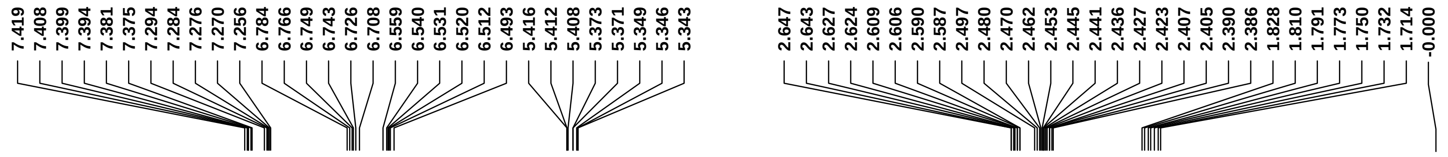
7.419
7.408
7.399
7.394
7.381
7.375
7.294
7.284
7.276
7.270
7.256
6.784
6.766
6.749
6.743
6.726
6.708
6.559
6.540
6.531
6.520
6.512
6.493
5.416
5.412
5.408
5.373
5.371
5.349
5.346
5.343
2.647
2.643
2.627
2.624
2.609
2.606
2.590
2.587
2.497
2.480
2.470
2.462
2.453
2.445
2.441
2.436
2.427
2.423
2.407
2.405
2.390
2.386
1.828
1.810
1.791
1.773
1.750
1.732
1.714
-0.000

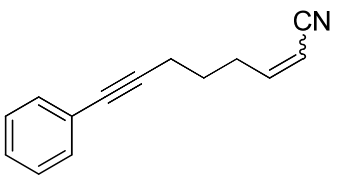
CN

2t

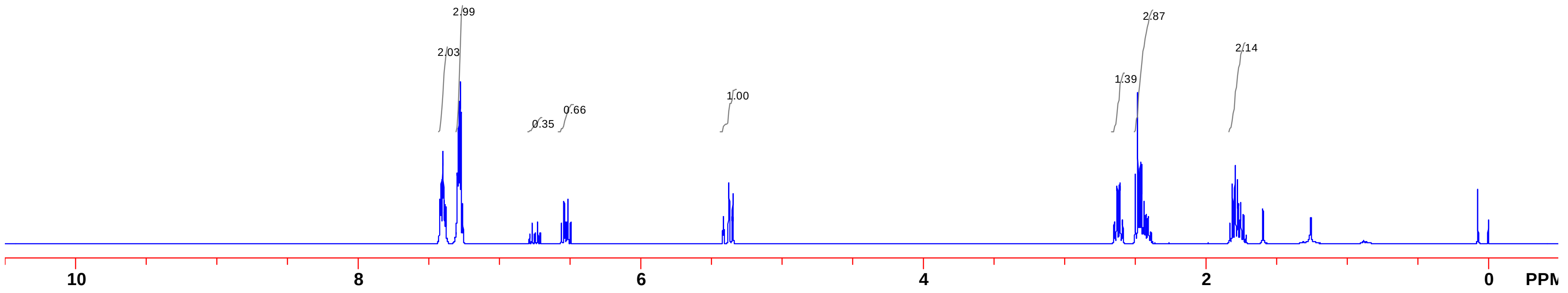
2.03
2.99
0.35
0.66
1.00
1.39
2.87
2.14
10
8
6
4
2
0
PPM

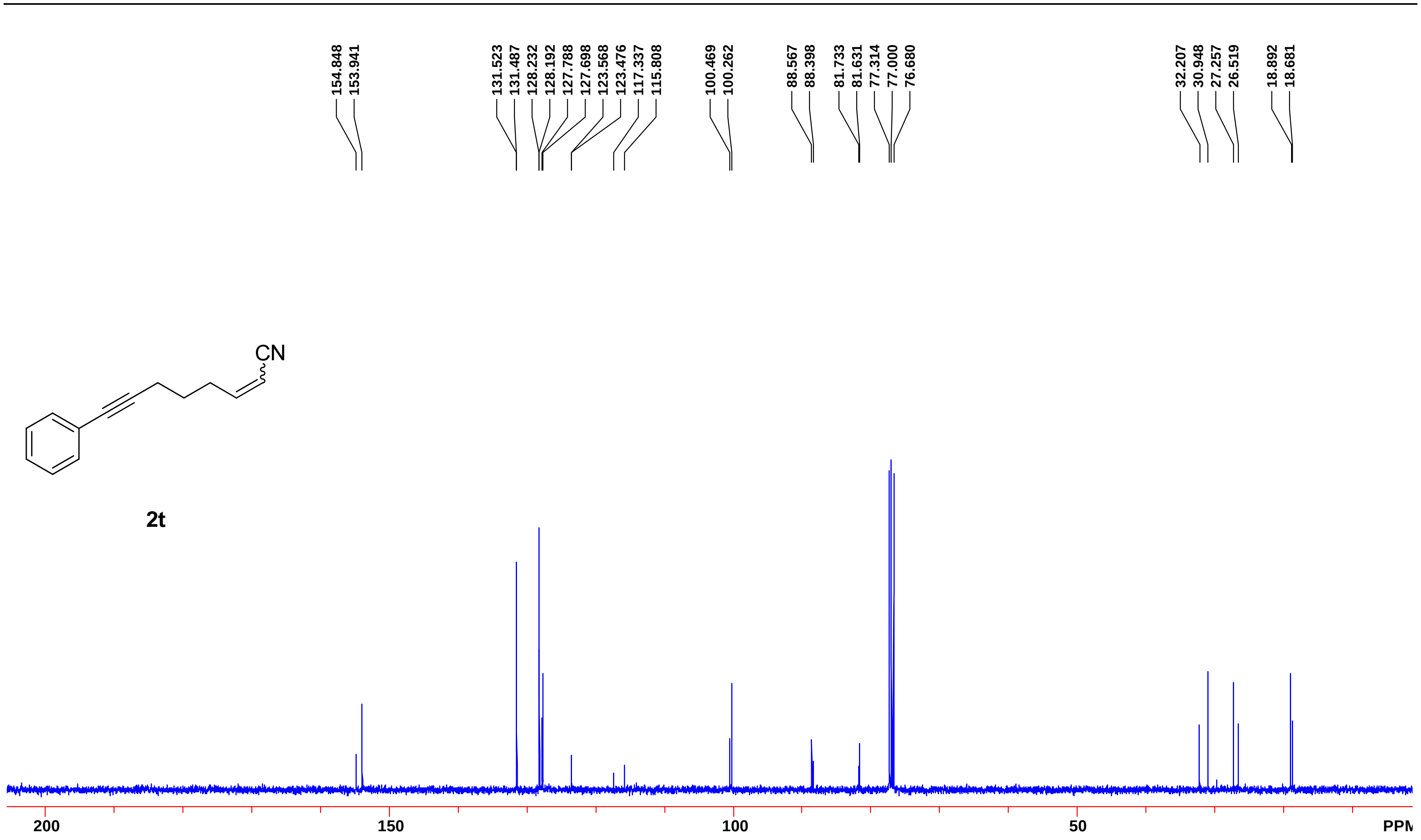
154.848
153.941
131.523
131.487
128.232
128.192
127.788
127.698
123.568
123.476
117.337
115.808
100.469
100.262
88.567
88.398
81.733
81.631
77.314
77.000
76.680
32.207
30.948
27.257
26.519
18.892
18.681
CN
2t
200
150
100
50
PPM

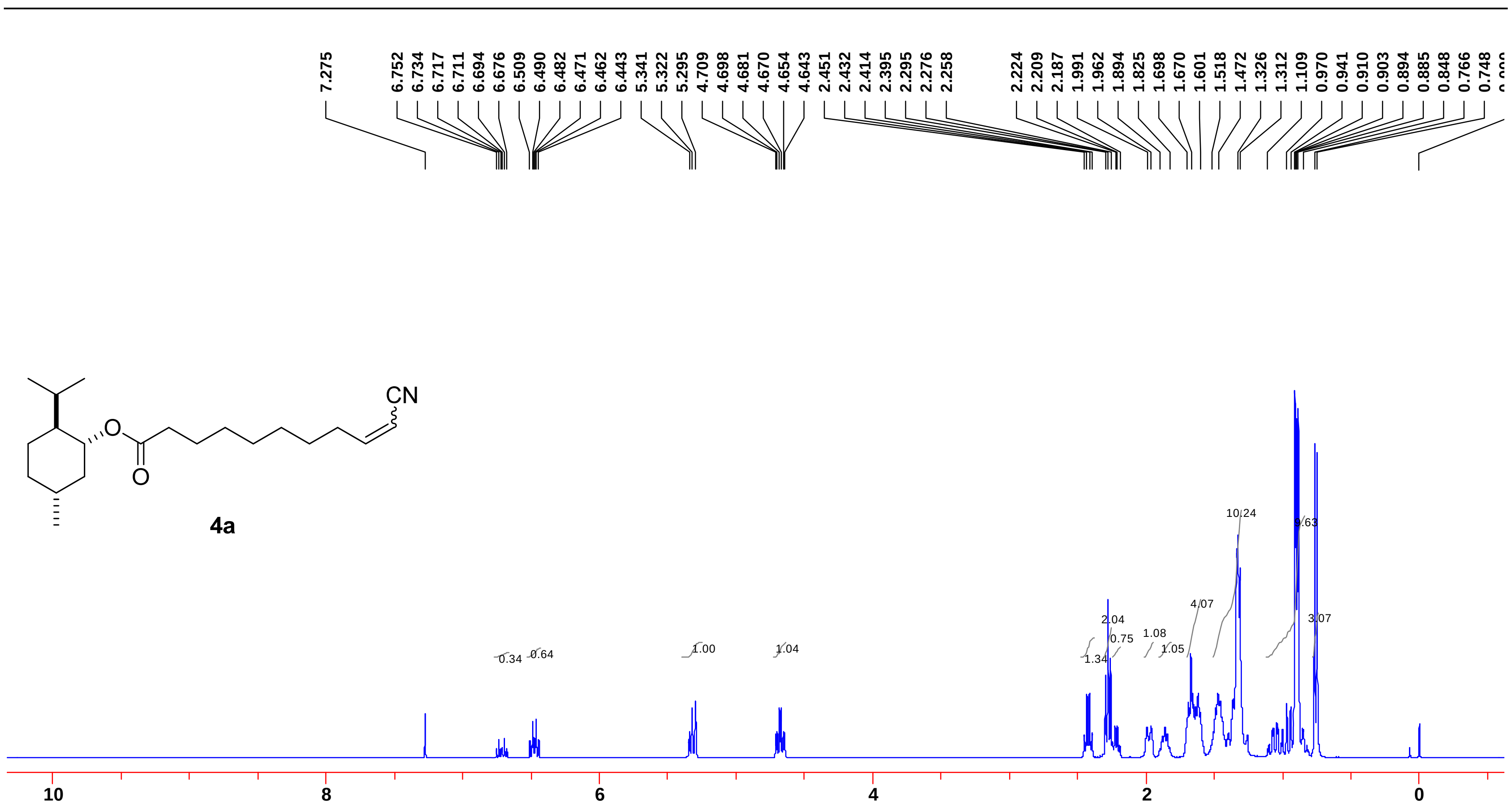
7.275
6.752
6.734
6.717
6.711
6.694
6.676
6.509
6.490
6.482
6.471
6.462
6.443
5.341
5.322
5.295
4.709
4.698
4.681
4.670
4.654
4.643
2.451
2.432
2.414
2.395
2.295
2.276
2.258
2.224
2.209
2.187
1.991
1.962
1.894
1.825
1.698
1.670
1.601
1.518
1.472
1.326
1.312
1.109
0.970
0.941
0.910
0.903
0.894
0.885
0.848
0.766
0.748
CN
O
O
4a
0.34
0.64
1.00
1.04
1.34
2.04
0.75
1.08
1.05
4.07
10.24
9.63
3.07
10
8
6
4
2
0

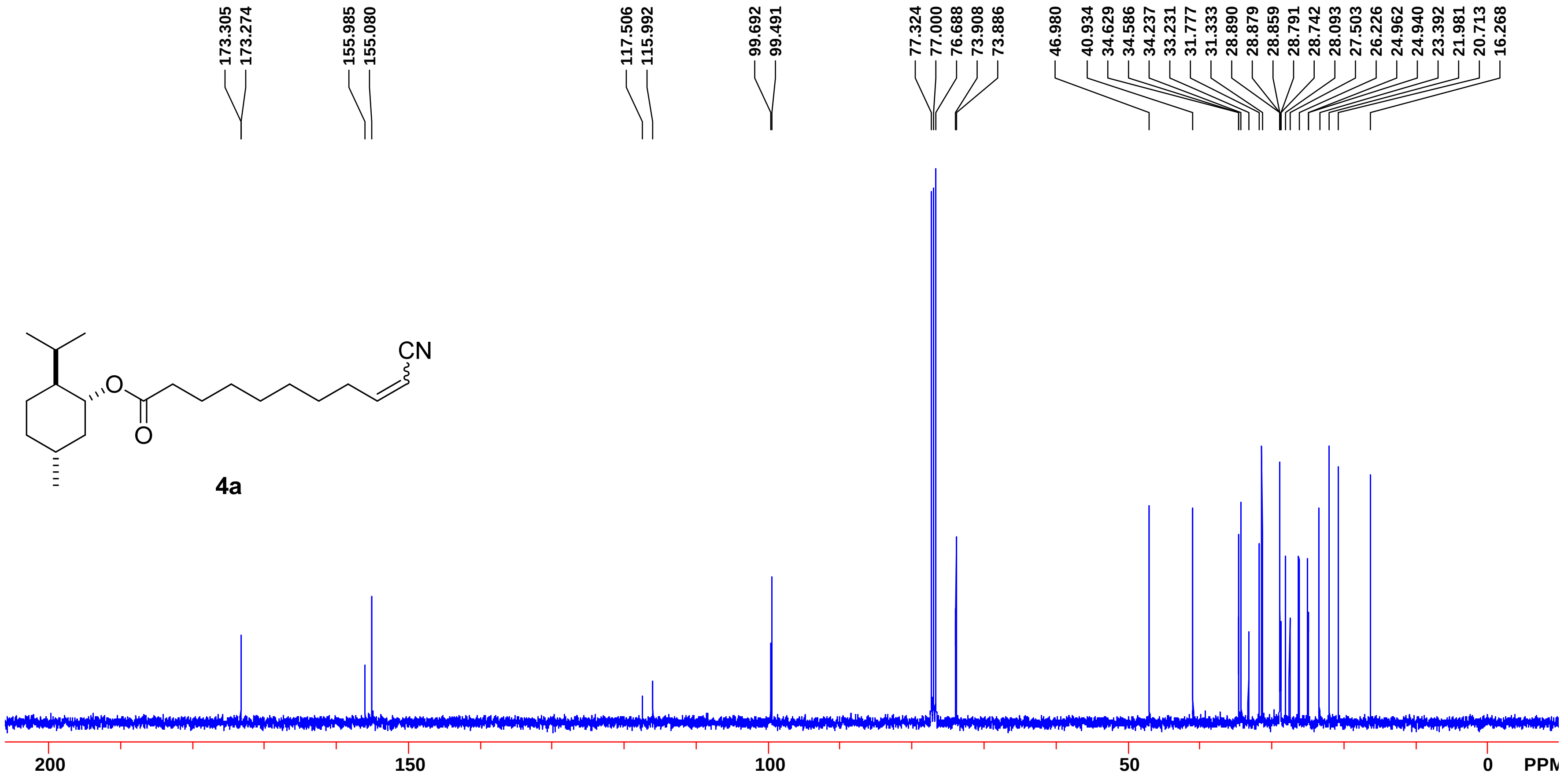
173.305
173.274
155.985
155.080
117.506
115.992
99.692
99.491
77.324
77.000
76.688
73.908
73.886
46.980
40.934
34.629
34.586
34.237
33.231
31.777
31.333
28.890
28.879
28.859
28.791
28.742
28.093
27.503
26.226
24.962
24.940
23.392
21.981
20.713
16.268
CN
O
O
4a
200
150
100
50
0
PPM

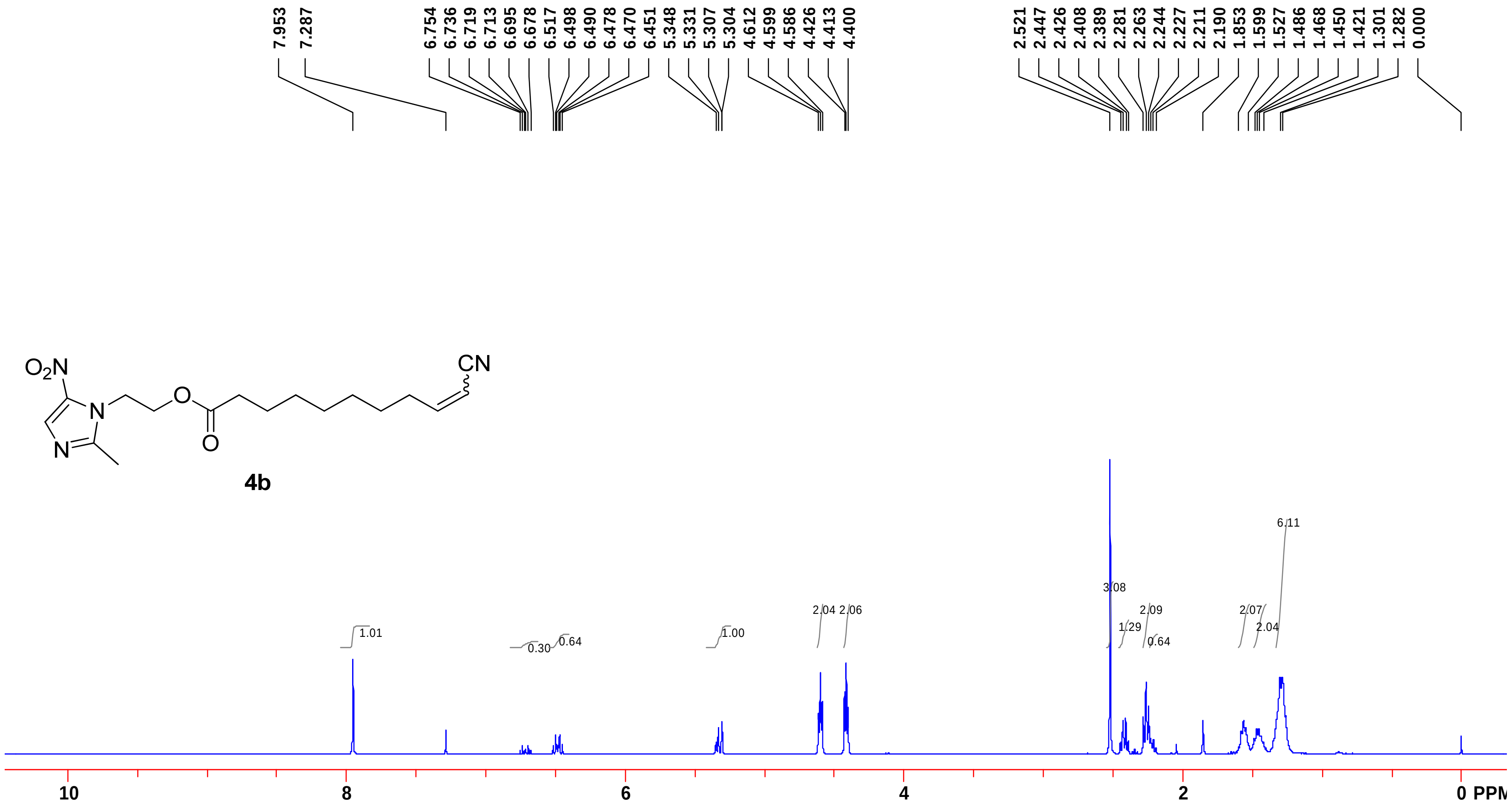
7.953
7.287
6.754
6.736
6.719
6.713
6.695
6.678
6.517
6.498
6.490
6.478
6.470
6.451
5.348
5.331
5.307
5.304
4.612
4.599
4.586
4.426
4.413
4.400
2.521
2.447
2.426
2.408
2.389
2.281
2.263
2.244
2.227
2.211
2.190
1.853
1.599
1.527
1.486
1.468
1.450
1.421
1.301
1.282
0.000
1.01
0.30
0.64
1.00
2.04
2.06
3.08
1.29
2.09
0.64
2.07
2.04
6.11
10
8
6
4
2
0 PPM

4b

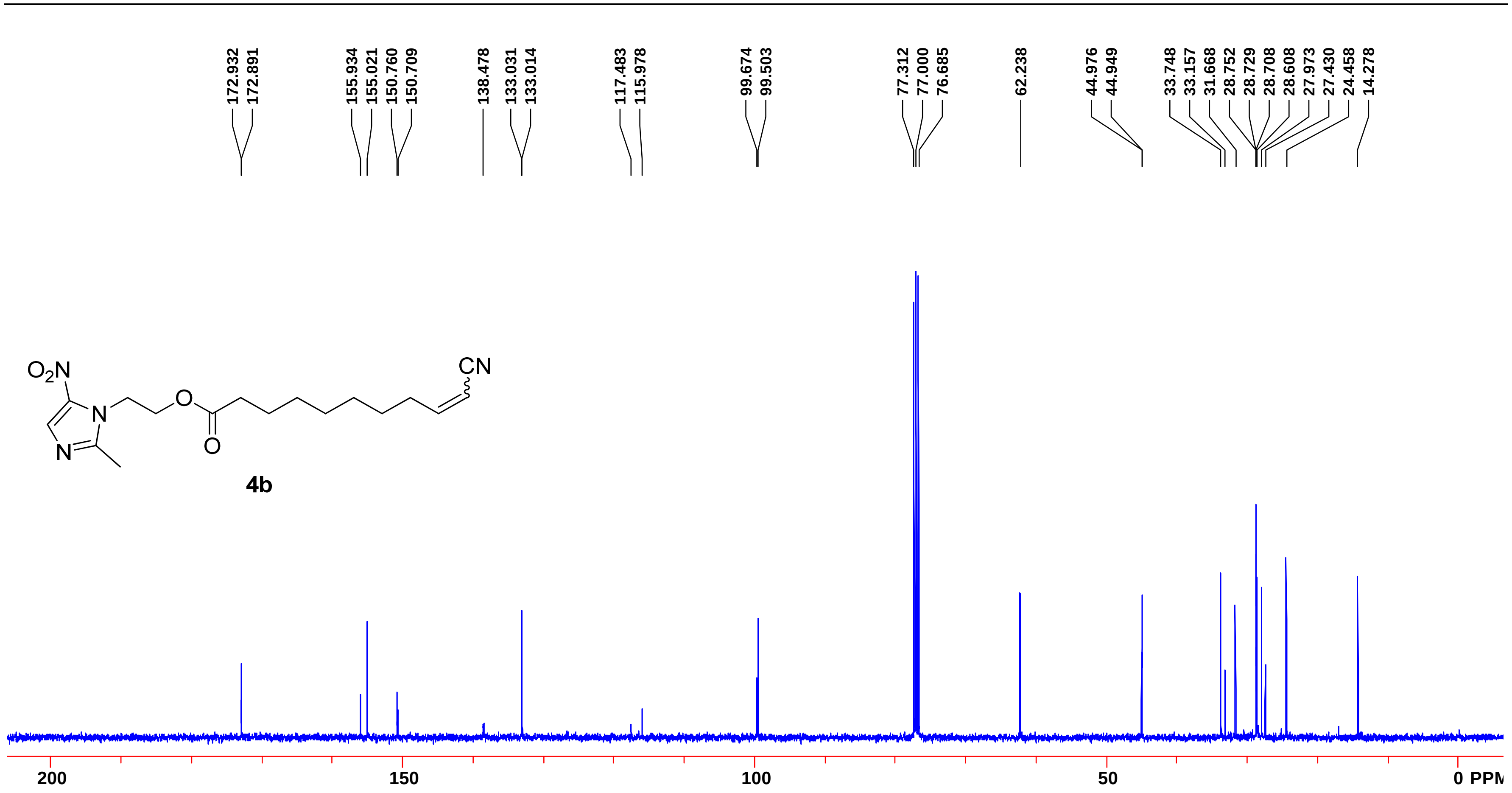
O2N
N
N
O
O
CN

4b

172.932
172.891
155.934
155.021
150.760
150.709
138.478
133.031
133.014
117.483
115.978
99.674
99.503
77.312
77.000
76.685
62.238
44.976
44.949
33.748
33.157
31.668
28.752
28.729
28.708
28.608
27.973
27.430
24.458
14.278
200
150
100
50
0 PPM

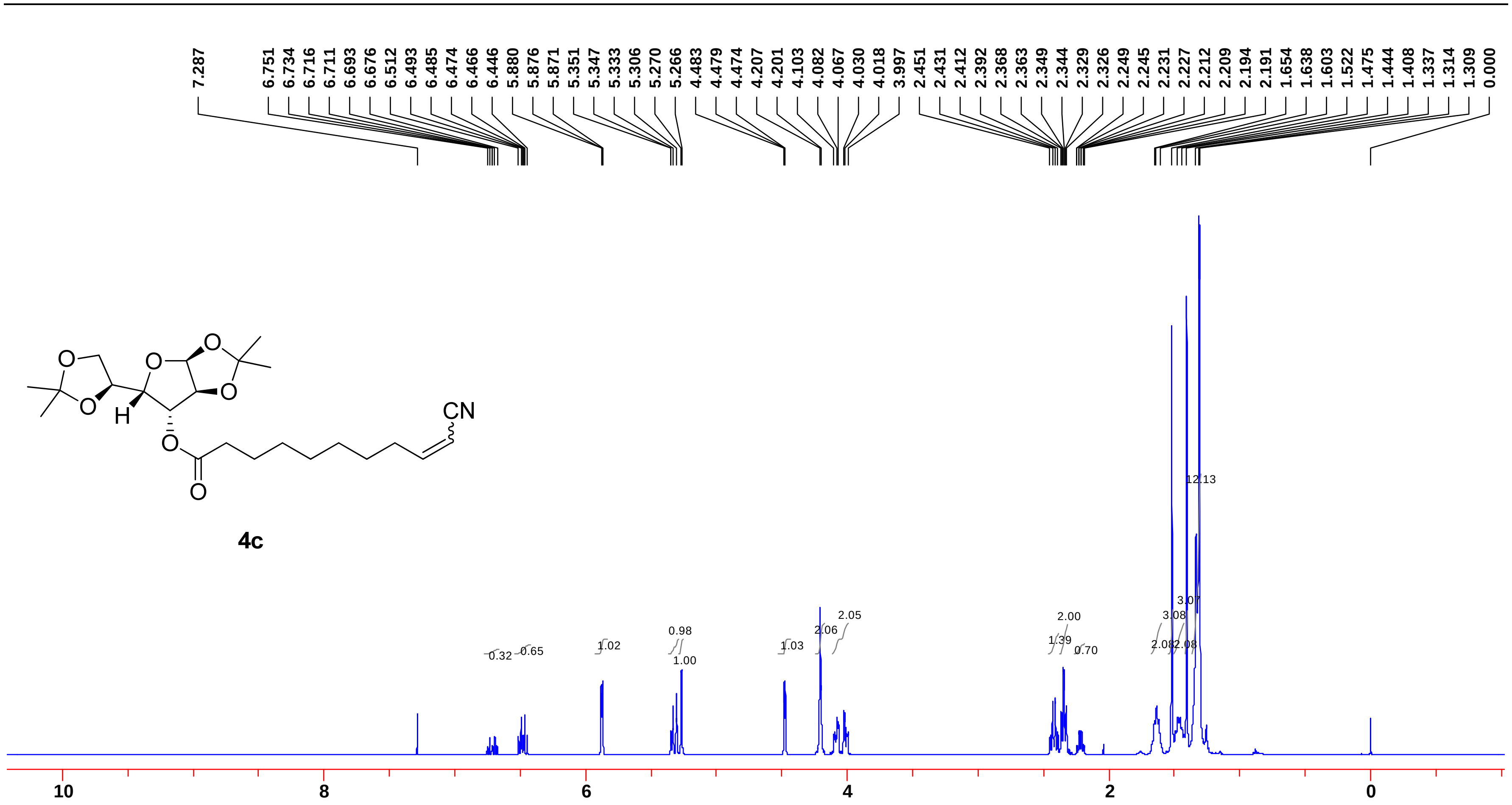
O
O
O
O
O
H
O
O
CN
4c
7.287
6.751
6.734
6.716
6.711
6.693
6.676
6.512
6.493
6.485
6.474
6.466
6.446
5.880
5.876
5.871
5.351
5.347
5.333
5.306
5.270
5.266
4.483
4.479
4.474
4.207
4.201
4.103
4.082
4.067
4.030
4.018
3.997
2.451
2.431
2.412
2.392
2.368
2.363
2.349
2.344
2.329
2.326
2.249
2.245
2.231
2.227
2.212
2.209
2.194
2.191
1.654
1.638
1.603
1.522
1.475
1.444
1.408
1.337
1.314
1.309
0.000
10
8
6
4
2
0

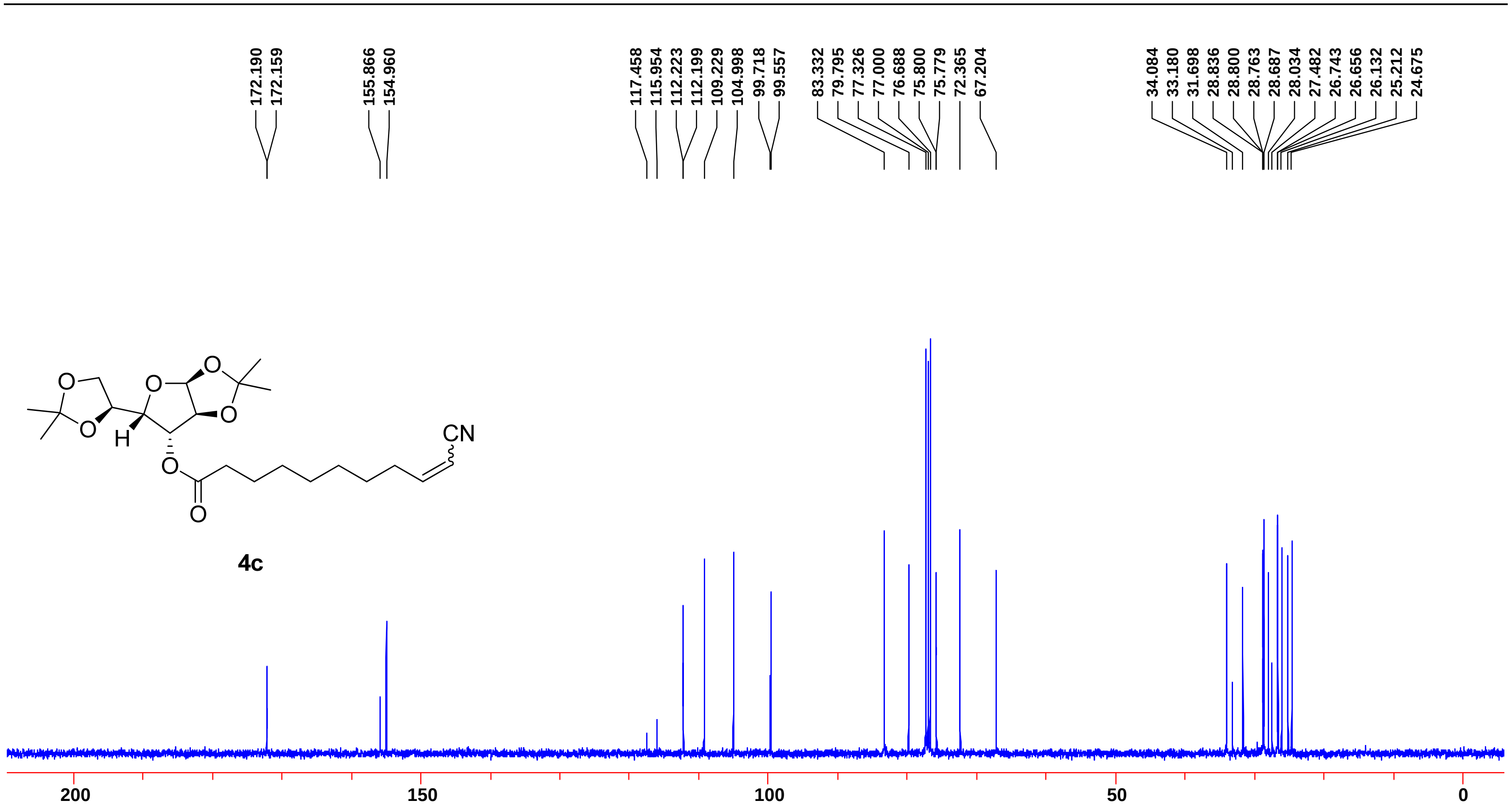
172.190
172.159
155.866
154.960
117.458
115.954
112.223
112.199
109.229
104.998
99.718
99.557
83.332
79.795
77.326
77.000
76.688
75.800
75.779
72.365
67.204
34.084
33.180
31.698
28.836
28.800
28.763
28.687
28.034
27.482
26.743
26.656
26.132
25.212
24.675
CN
H
4c
200
150
100
50
0

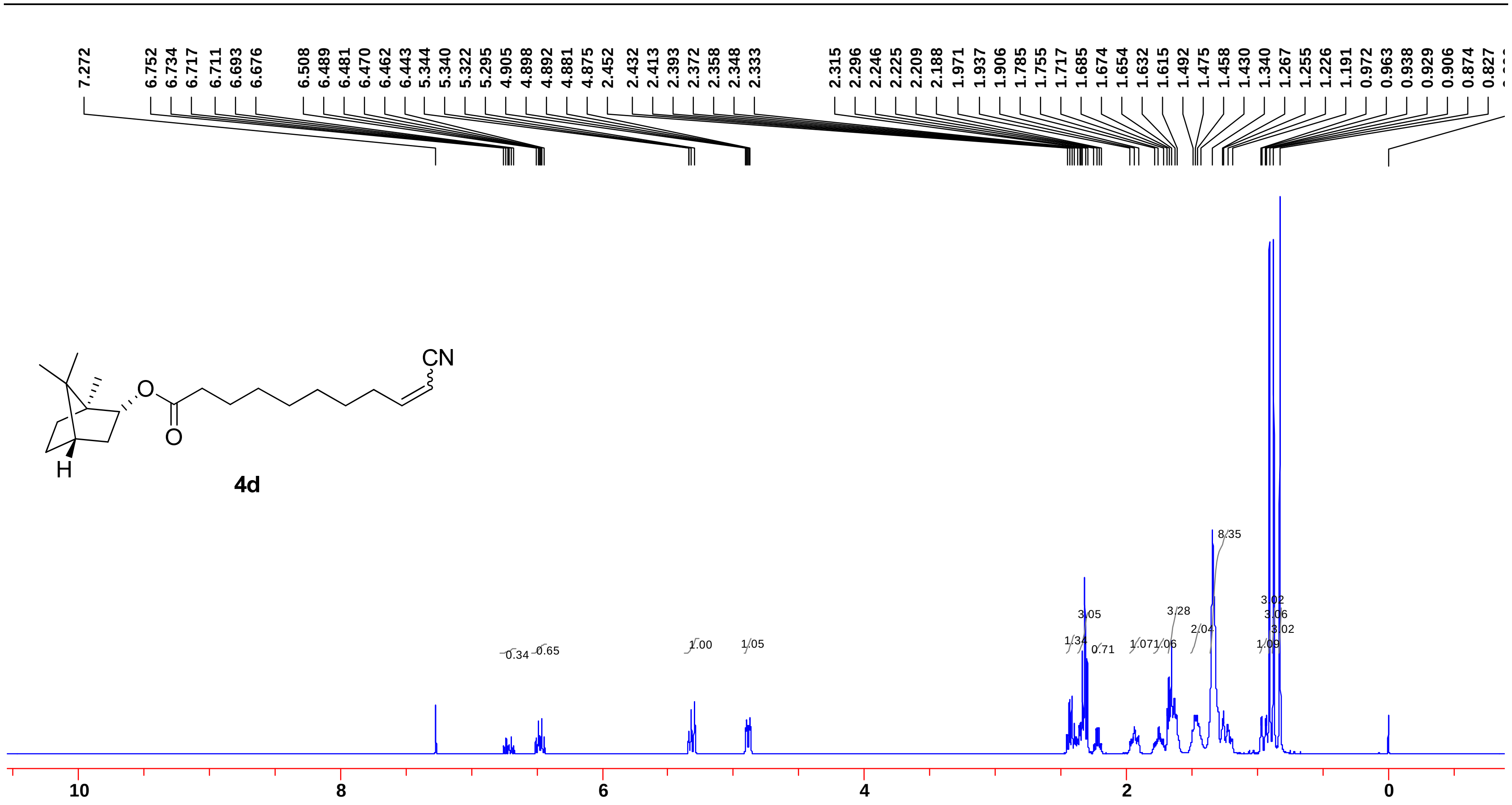
7.272
6.752
6.734
6.717
6.711
6.693
6.676
6.508
6.489
6.481
6.470
6.462
6.443
5.344
5.340
5.322
5.295
4.905
4.898
4.892
4.881
4.875
2.452
2.432
2.413
2.393
2.372
2.358
2.348
2.333
2.315
2.296
2.246
2.225
2.209
2.188
1.971
1.937
1.906
1.785
1.755
1.717
1.685
1.674
1.654
1.632
1.615
1.492
1.475
1.458
1.430
1.340
1.267
1.255
1.226
1.191
0.972
0.963
0.938
0.929
0.906
0.874
0.827
CN
O
O
H
4d
0.34
0.65
1.00
1.05
1.34
3.05
0.71
1.07
1.06
3.28
2.04
8.35
1.09
3.02
3.06
3.02
10
8
6
4
2
0

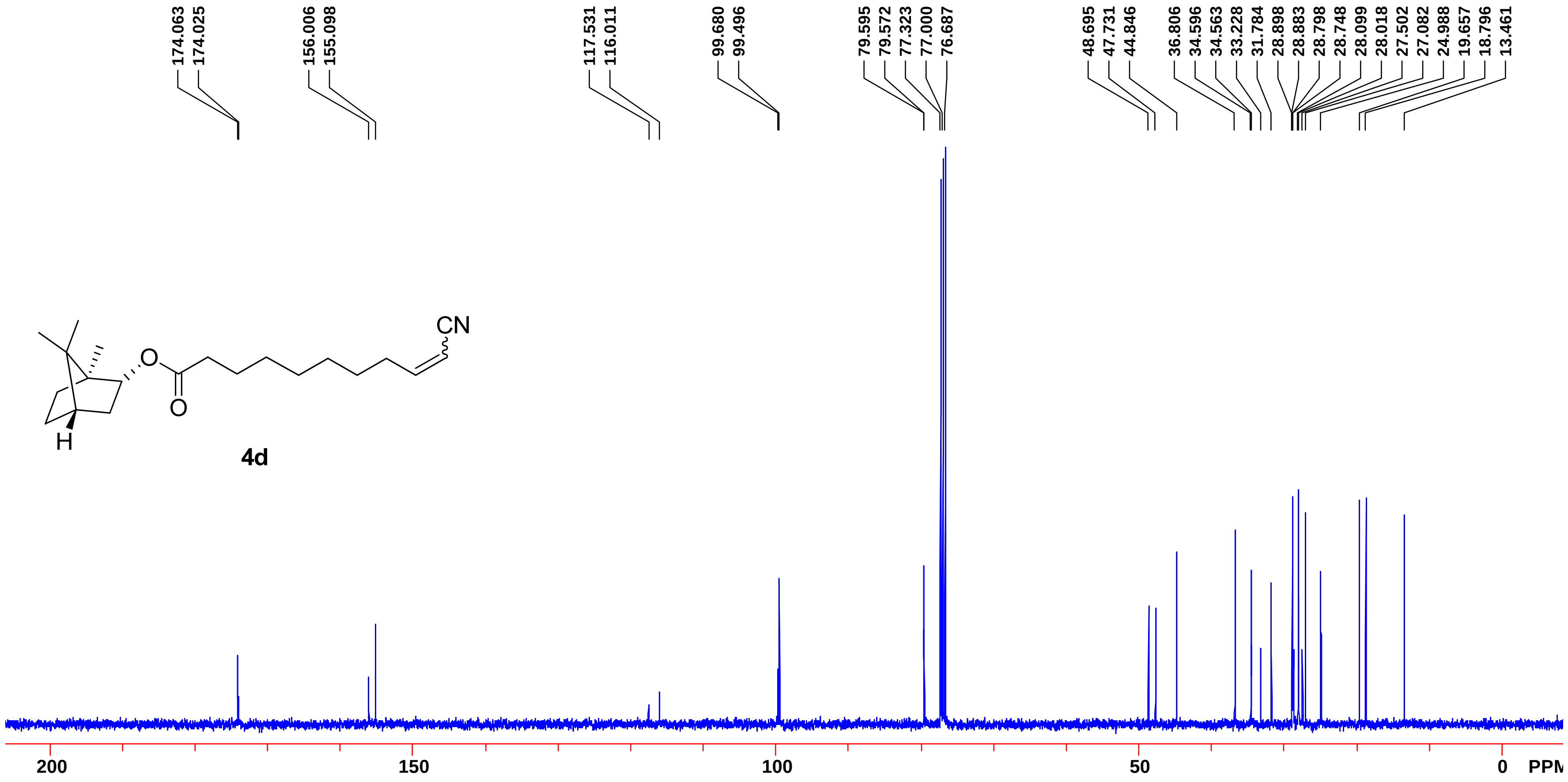
174.063
174.025
156.006
155.098
117.531
116.011
99.680
99.496
79.595
79.572
77.323
77.000
76.687
48.695
47.731
44.846
36.806
34.596
34.563
33.228
31.784
28.898
28.883
28.798
28.748
28.099
28.018
27.502
27.082
24.988
19.657
18.796
13.461
CN
O
O
H
200
150
100
50
0
PPM

4d

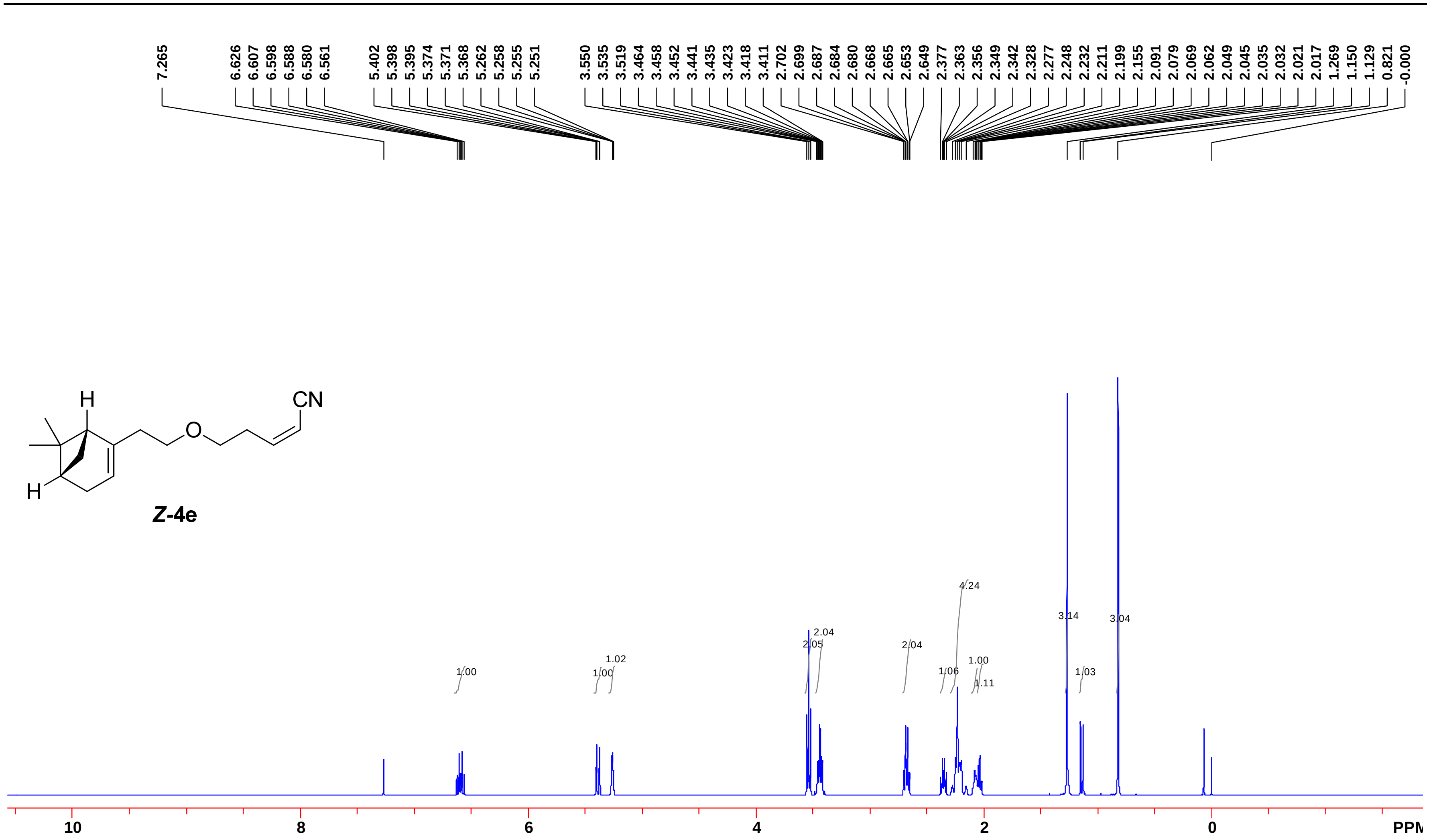
7.265
6.626
6.607
6.598
6.588
6.580
6.561
5.402
5.398
5.395
5.374
5.371
5.368
5.262
5.258
5.255
5.251
3.550
3.535
3.519
3.464
3.458
3.452
3.441
3.435
3.423
3.418
3.411
2.702
2.699
2.687
2.684
2.680
2.668
2.665
2.653
2.649
2.377
2.363
2.356
2.349
2.342
2.328
2.277
2.248
2.232
2.211
2.199
2.155
2.091
2.079
2.069
2.062
2.049
2.045
2.035
2.032
2.021
2.017
1.269
1.150
1.129
0.821
-0.000
H
CN
O
H
Z-4e
1.00
1.00
1.02
2.05
2.04
2.04
1.06
4.24
1.00
1.11
3.14
1.03
3.04
10
8
6
4
2
0
PPM

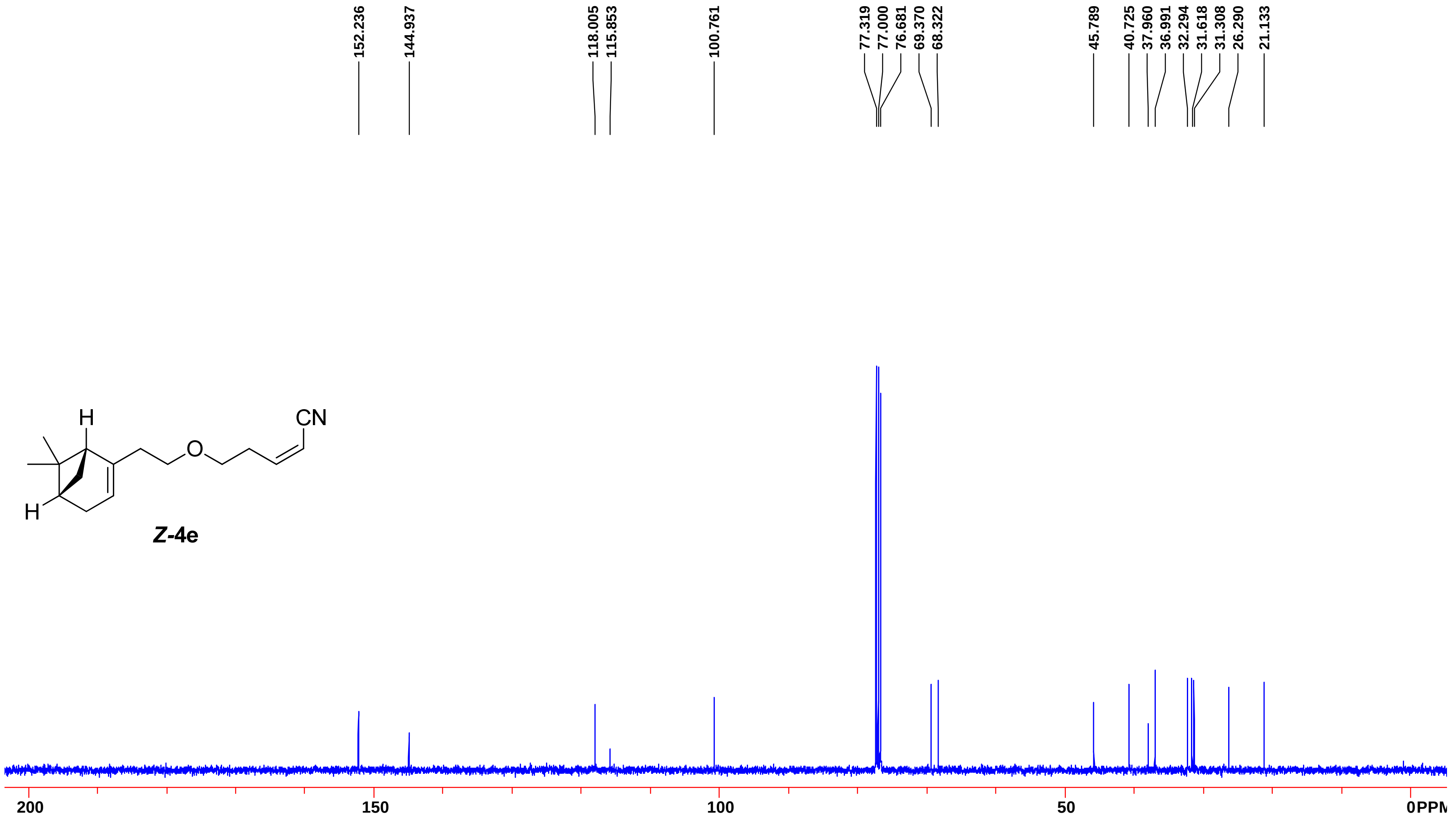
152.236
144.937
118.005
115.853
100.761
77.319
77.000
76.681
69.370
68.322
45.789
40.725
37.960
36.991
32.294
31.618
31.308
26.290
21.133
200
150
100
50
0PPM
H
CN
O
H

***Z*-4e**

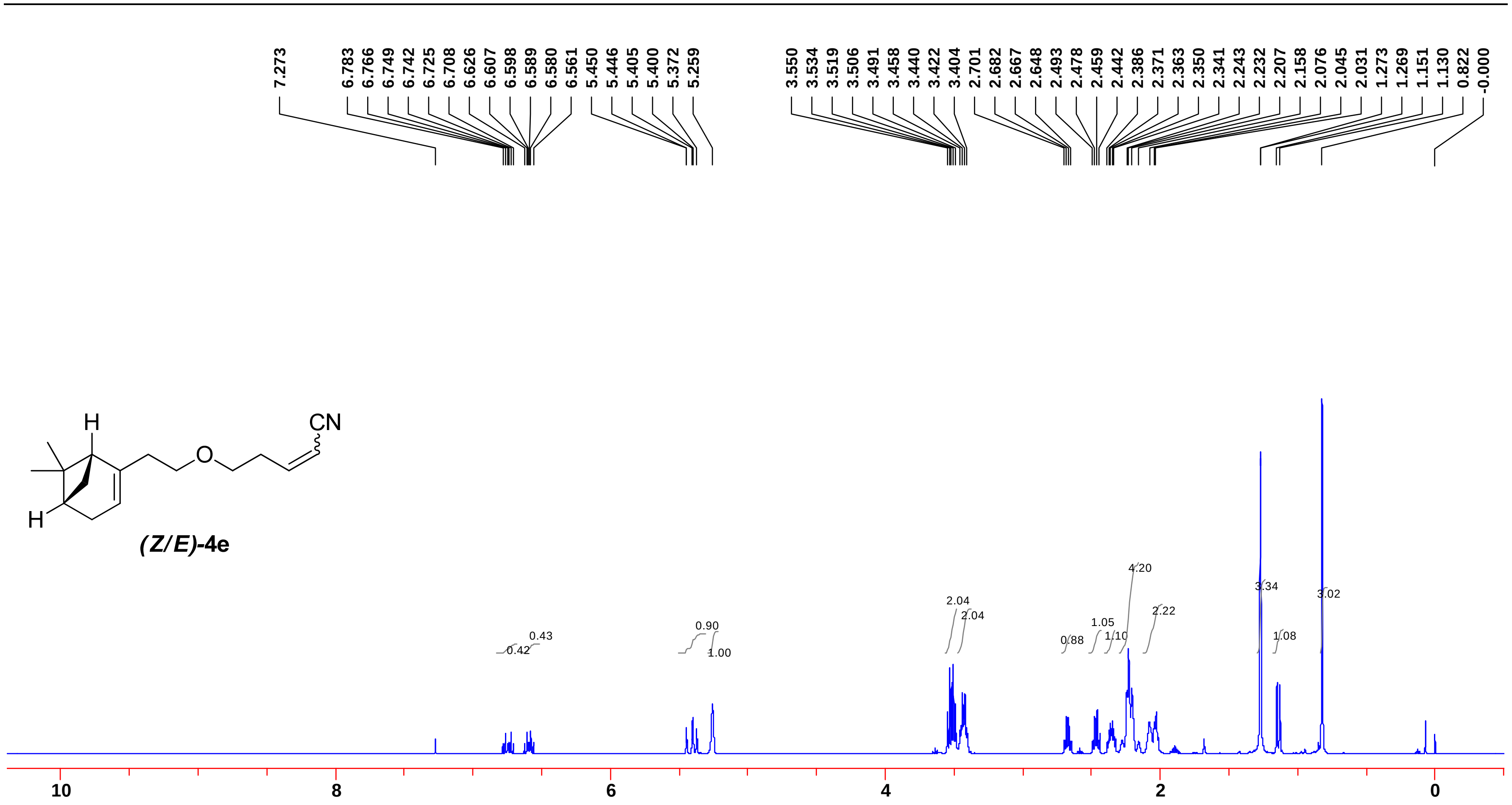
7.273
6.783
6.766
6.749
6.742
6.725
6.708
6.626
6.607
6.598
6.589
6.580
6.561
5.450
5.446
5.405
5.400
5.372
5.259
3.550
3.534
3.519
3.506
3.491
3.458
3.440
3.422
3.404
2.701
2.682
2.667
2.648
2.493
2.478
2.459
2.442
2.386
2.371
2.363
2.350
2.341
2.243
2.232
2.207
2.158
2.076
2.045
2.031
1.273
1.269
1.151
1.130
0.822
-0.000
H
H
O
CN
0.42
0.43
0.90
1.00
2.04
2.04
0.88
1.05
1.10
4.20
2.22
3.34
1.08
3.02
10
8
6
4
2
0

(Z/E)-4e

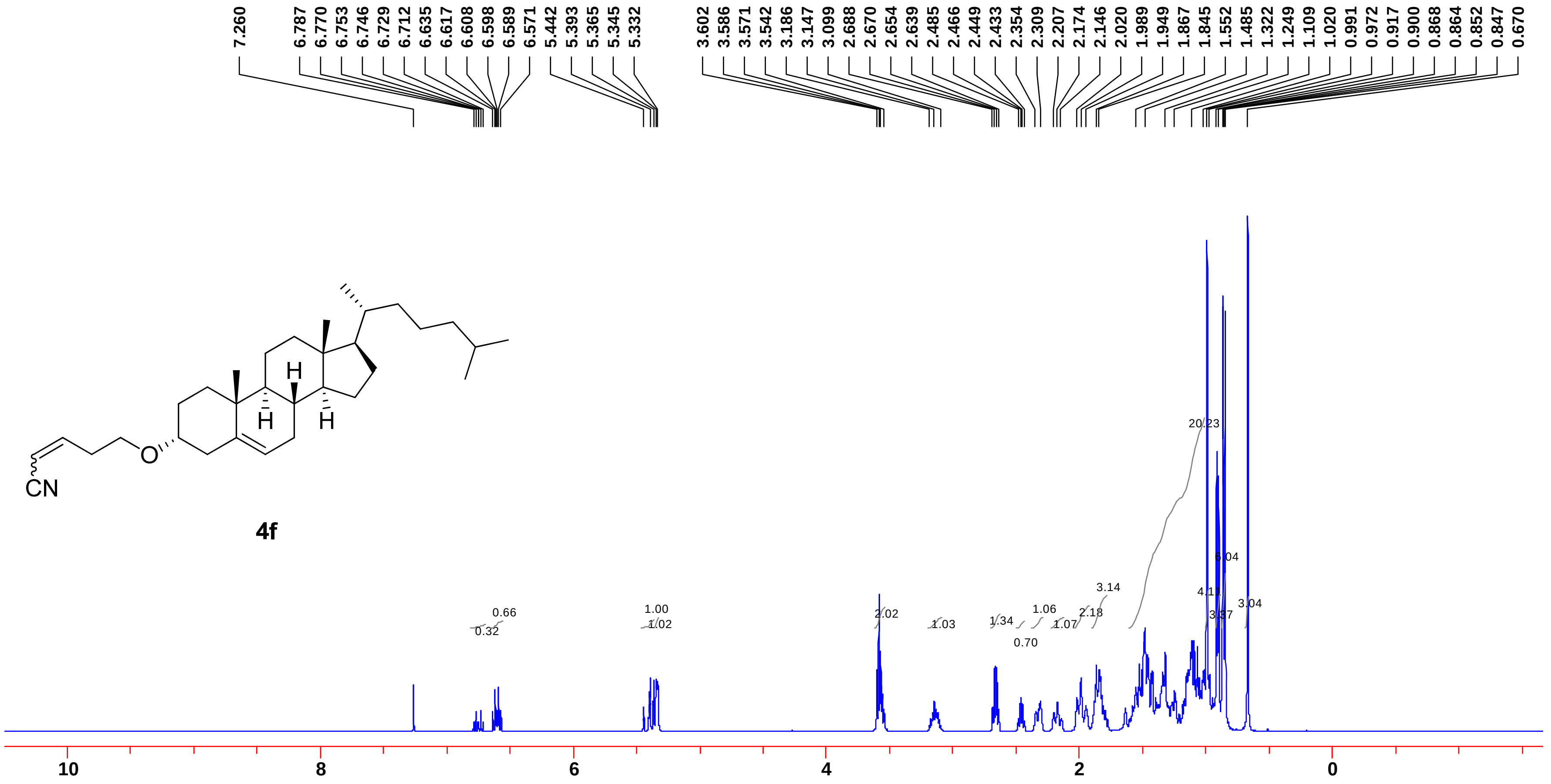
4f
CN
O
H
10
8
6
4
2
0

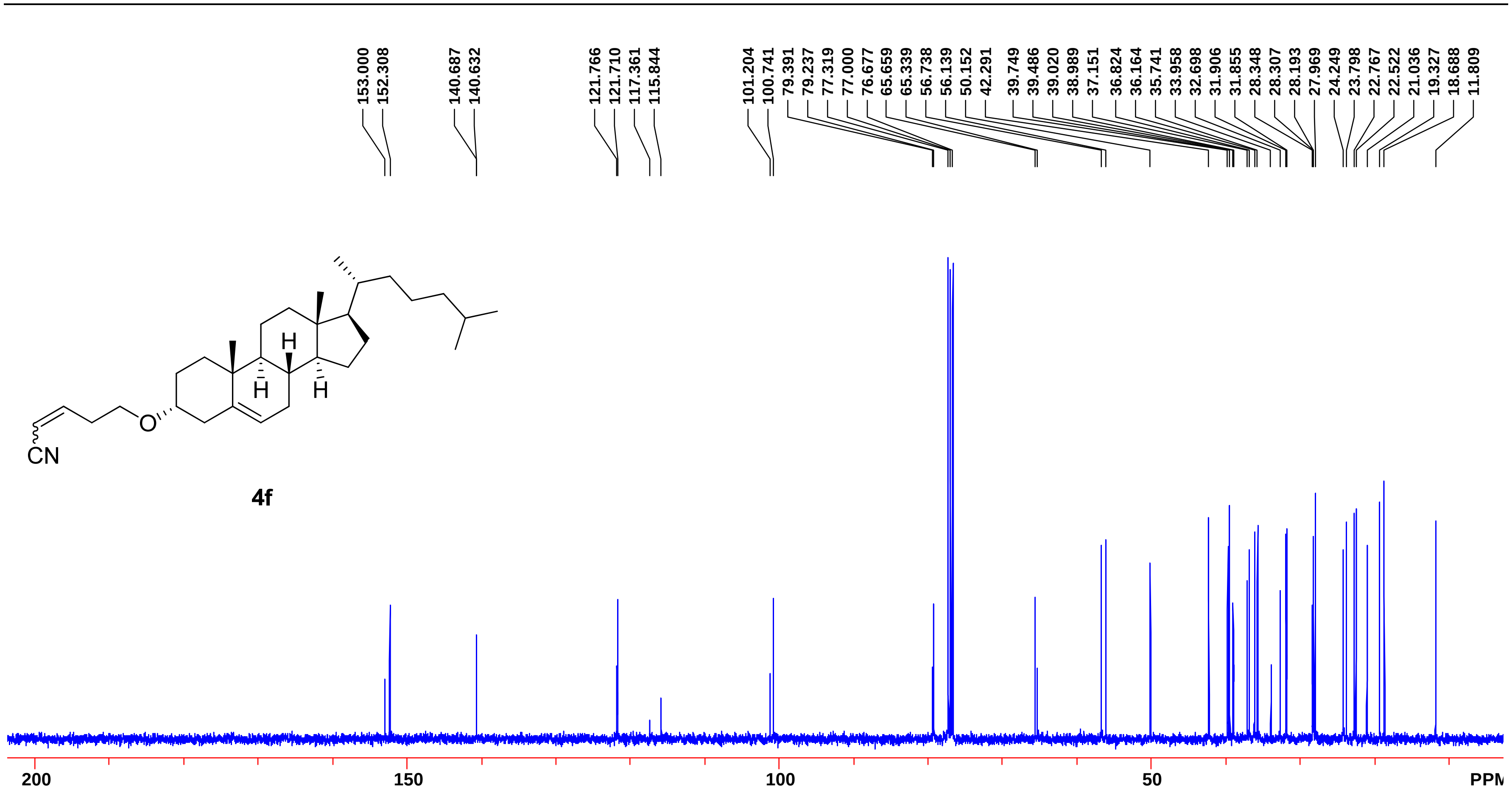
153.000
152.308
140.687
140.632
121.766
121.710
117.361
115.844
101.204
100.741
79.391
79.237
77.319
77.000
76.677
65.659
65.339
56.738
56.139
50.152
42.291
39.749
39.486
39.020
38.989
37.151
36.824
36.164
35.741
33.958
32.698
31.906
31.855
28.348
28.307
28.193
27.969
24.249
23.798
22.767
22.522
21.036
19.327
18.688
11.809
H
H
H
O
CN
4f
200
150
100
50
